# A low-dimensional structure of neurological impairment in stroke

**DOI:** 10.1093/braincomms/fcab119

**Published:** 2021-06-03

**Authors:** Antonio Luigi Bisogno, Chiara Favaretto, Andrea Zangrossi, Elena Monai, Silvia Facchini, Serena De Pellegrin, Lorenzo Pini, Marco Castellaro, Anna Maria Basile, Claudio Baracchini, Maurizio Corbetta

**Affiliations:** Department of Neuroscience, University of Padova, Padova 35100, Italy; Padova Neuroscience Center (PNC), University of Padova, Padova 35100, Italy; Department of Neuroscience, University of Padova, Padova 35100, Italy; Padova Neuroscience Center (PNC), University of Padova, Padova 35100, Italy; Department of Neuroscience, University of Padova, Padova 35100, Italy; Padova Neuroscience Center (PNC), University of Padova, Padova 35100, Italy; Department of Neuroscience, University of Padova, Padova 35100, Italy; Padova Neuroscience Center (PNC), University of Padova, Padova 35100, Italy; Azienda Ospedaliera Università di Padova, Padova 35100, Italy; Department of Neuroscience, University of Padova, Padova 35100, Italy; Padova Neuroscience Center (PNC), University of Padova, Padova 35100, Italy; Azienda Ospedaliera Università di Padova, Padova 35100, Italy; Padova Neuroscience Center (PNC), University of Padova, Padova 35100, Italy; Department of Information Engineering, University of Padova, Padova 35100, Italy; Azienda Ospedaliera Università di Padova, Padova 35100, Italy; Department of Neuroscience, University of Padova, Padova 35100, Italy; Azienda Ospedaliera Università di Padova, Padova 35100, Italy; Department of Neuroscience, University of Padova, Padova 35100, Italy; Padova Neuroscience Center (PNC), University of Padova, Padova 35100, Italy; Azienda Ospedaliera Università di Padova, Padova 35100, Italy; Venetian Institute of Molecular Medicine, Padova 35100, Italy

**Keywords:** stroke, biomarkers, behavioural, dimensionality

## Abstract

Neurological deficits following stroke are traditionally described as syndromes related to damage of a specific area or vascular territory. Recent studies indicate that, at the population level, post-stroke neurological impairments cluster in three sets of correlated deficits across different behavioural domains. To examine the reproducibility and specificity of this structure, we prospectively studied first-time stroke patients (*n* = 237) using a bedside, clinically applicable, neuropsychological assessment and compared the behavioural and anatomical results with those obtained from a different prospective cohort studied with an extensive neuropsychological battery. The behavioural assessment at 1-week post-stroke included the Oxford Cognitive Screen and the National Institutes of Health Stroke Scale. A principal component analysis was used to reduce variables and describe behavioural variance across patients. Lesions were manually segmented on structural scans. The relationship between anatomy and behaviour was analysed using multivariate regression models. Three principal components explained ≈50% of the behavioural variance across subjects. PC1 loaded on language, calculation, praxis, right side neglect and memory deficits; PC2 loaded on left motor, visual and spatial neglect deficits; PC3 loaded on right motor deficits. These components matched those obtained with a more extensive battery. The underlying lesion anatomy was also similar. Neurological deficits following stroke are correlated in a low-dimensional structure of impairment, related neither to the damage of a specific area or vascular territory. Rather they reflect widespread network impairment caused by focal lesions. These factors showed consistency across different populations, neurobehavioural batteries and, most importantly, can be described using a combination of clinically applicable batteries (National Institutes of Health Stroke Scale and Oxford Cognitive Screen). They represent robust behavioural biomarkers for future stroke population studies.

## Teaser

Neurological deficits following stroke are traditionally described as syndromes related to damage of a specific area or vascular territory. Bisogno et al. show that post-stroke neurological impairment can be described by robust, reproducible components of correlated deficits across functional domains with a bedside, clinically suitable, neurobehavioural assessment.

## Graphical Abstract

**Graphical Abstract:**
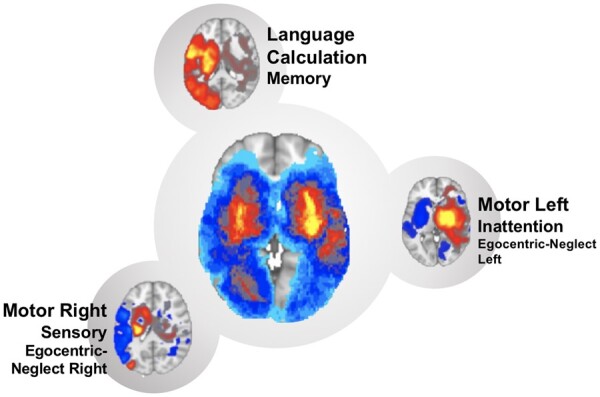

## Introduction

‘You learn neurology stroke by stroke’ C.M. Fisher (1961)

Neurologists traditionally classify behavioural syndromes based on the damage of specific brain regions (e.g. Broca aphasia) or the vascular distribution of stroke [e.g. middle cerebral artery (MCA)]. When behavioural deficits are correlated the explanation is that adjacent cortical regions suffer from the injury, be it ischaemia, as in right hemiplegia and Broca aphasia, or abnormal electrical activity, as in the Jacksonian march.^1–3^ Dr Fisher described more than 70 different syndromes caused by focal ischaemia in his work.^1^

However, recent work offers a different perspective showing that syndrome-based descriptions do not characterize behavioural deficits at the population level. For instance, the examination of samples of stroke patients with the National Institutes of Health Stroke Scale (NIHSS) identifies two factors: one for left and one for right hemisphere lesions, which split respectively in a cognitive and sensory-motor component, accounting for approximately 80% of behavioural variability across subjects.^4^^,^^5^

Since cognitive deficits are only cursorily measured by the NIHSS, this simplified model may reflect a lack of sensitivity for impairment in multiple cognitive domains. However, a more recent analysis in a prospective sample of stroke patients (*n* = 132), tested with an extensive neuropsychological battery (44 tests covering multiple domains: language, motor, vision, memory, attention) at Washington University (WU) in St. Louis, discovered that three deficit components account for the majority (65%) of variability in performance.^6^ These factors remained at three-twelve months post-stroke, tracked recovery, and could represent biomarkers of impairment.^7^ The first factor loaded on language, including deficits of language expression and comprehension, and memory, both verbal and spatial. The second and third factors loaded on the contralateral motor and visual attention deficits, i.e. left deficits for right lesions, and vice versa. Neither local damage nor vascular distributions could account for the observed correlation of deficits in different domains. Instead, a strong relationship was observed with functional network damage measured with fMRI.^8–11^

The first aim of this study was to validate through a short and clinically applicable assessment the previously identified structure of impairment in a different population: Veneto, Italy. We used the Oxford Cognitive Screening (OCS), specially developed by the late psychologist Glyn Humphreys and colleagues to study post-stroke cognitive impairment.^12^^,^^13^ This test covers language, memory, attention, calculation and praxis; it takes 10–15 min to be administered—against more than 2 h for the WU battery—and with the NIHSS may provide a clinically suitable neurobehavioural assessment applicable in busy stroke units. The data were analysed to find robust components of impairment that were correlated across patients. The results were then compared to those obtained by analysing the independent dataset from WU in the same manner. The second aim was to examine the neuroanatomy of these factors using a multivariate machine learning approach. We related spatial patterns of damage to behavioural scores to find a lesion model that best accounted for the individual variability of scores. To replicate the neuroanatomy, we ran the same approach on the WU cohort.

## Materials and methods

### Study sample

The recruitment covered 22 months, from December 2017 to October 2019, and occurred at the Stroke Unit and Clinica Neurologica of the Hospital of Padova and the Stroke Unit of the Ospedale S. Antonio Padova.

The inclusion criteria, same as in the WU cohort, included:

1. Age 18 or higher;
2. First symptomatic stroke, ischaemic or haemorrhagic in aetiology;
3. Up to two lacunes, clinically silent, less than 15 mm in size on CT scan;
4. Time of enrolment: <2 weeks from stroke onset;
5. Awake, alert, and capable of participating in research.

Exclusion criteria included: (i) Previous stroke based on clinical imaging; (ii) Multifocal strokes; (iii) Inability to maintain wakefulness in the course of testing; (iv) More than two asymptomatic lesions on CT scan; (v) Presence of central nervous system tumours; (vi) History of dementia; (vii) Previous central nervous system surgeries; (viii) Schizophrenia, bipolar disorder, major depression or other severe psychiatric conditions; (ix) Other medical conditions that preclude active participation in research and may alter the interpretation of the behavioural/imaging studies; and (x) Inability to provide consent; for severe aphasic patients informed consent next-of-kin gave informed consent.

### Procedures

We screened a total of *N* = 1080 charts. Subjects (*n* = 237) with a first symptomatic stroke, ischaemic or haemorrhagic, were prospectively recruited, with *n* = 180 meeting post-enrolment inclusion criteria. Supplementary Figure 1 describes the enrolment flowchart and shows reasons for lack of inclusion. Subjects were evaluated with a neurobehavioural battery at the acute phase (5 ± 3.3 days post-stroke). The behavioural battery included the OCS^12^ and the NIHSS.^14^ We collected structural imaging (130 MRI scans and 50 CT scans) that was routinely performed for each subject at 5 ± 4 days post-stroke. Supplementary Figure 2 illustrates the design of the study.

### Measures

Experienced neurologists examined all patients using the NIHSS,^14^ which was administered on admission, on discharge and at the time of testing (within a week). The NIHSS includes 15 subtests: level of consciousness subtests, gaze and visual field deficits, facial palsy, upper and lower motor deficits (right and left side), limb ataxia, sensory impairment, inattention, dysarthria and language deficits. The NIHSS scores at the time of testing were analysed. Also, we recorded: demographics data, stroke risk factors, other neurological, psychological, or psychiatric conditions, familiarity for stroke, stroke subtype (haemorrhagic or ischaemic), clinical presentation.

The OCS was administered the first week following stroke onset. The OCS is a brief tool—10–15 min long—developed to describe acute cognitive impairment post-stroke.^12^ It is structured around five cognitive domains: language, praxis, number processing, attention and memory, and consists of 10 individual subtests. In the language domain, picture naming, picture pointing and sentence reading subtests measure speech production, auditory comprehension and reading, respectively. In the memory domain, verbal and spatial memory are examined separately through the orientation, recall and recognition, and episodic memory subtests. Number writing, and calculation tasks evaluate number processing. An imitating meaningless gestures test measures praxis. Finally, the broken heart test includes several subtests each measuring a different aspect of attention. The overall accuracy rate (across the two fields) is a measure of sustained attention, which is necessary for a high overall performance. The number of misses either on the left or right visual field is a measure of egocentric neglect. Finally, missing gaps on the left or right side of the individual hearts is a measure of allocentric neglect. Visual fields are checked separately.

### MRI and CT lesions

MRI and/or CT scans were routinely performed on admission and follow up depending on clinical status. Lesions were manually segmented on structural MRI and CT scans using the ITK-snap imaging software system^15^ and individually checked by a neurology resident and a board certified neurologist.^16^^,^^17^ CT and MRI segmented lesions were mapped on the MNI152 atlas using the Advanced Normalization Tools.^18^ The FSL software was used to create the overlap of individual lesions on a standard brain atlas, producing an overlay map of all lesions.^19^ Finally, to precisely describe stroke topography, lesions were mapped on the Harvard-Oxford cortical and subcortical structural masks.^20^

### Behavioural analysis

The statistical analysis included the OCS subtests scores and the NIHSS individual scores. All subtests were normalized to their maximum values, and sign-inverted, such that the largest values corresponded to the most severe level of deficit. Only patients who participated to all task sets were included. After having *z*-scored the behavioural scores, we used a principal component analysis (PCA) to reduce the number of variables and describe the variability of behavioural deficits. Since many variables were expected to be correlated, an oblique rotation (PROMAX) was used (for completeness non-rotated PCAs and the corresponding anatomical maps were computed). As the oblique rotation is dependent on the number of selected components, we decided to be consistent with Corbetta et al.,^6^ and selected the first three Principal Components (PCs).

Moreover, a correlation matrix was computed to graphically visualize the strength of correlation between tests. Many subtests were at ceiling, with most subjects reaching maximum scores. Matlab R2018b was used for all statistical analysis.

### Lesion-behaviour analysis

The analysis was run on our sample and a subset of patients of the WU cohort (*n* = 67 had completed all tests of the battery). To relate behavioural deficits to lesions, we employed a ridge regression algorithm (RR).^21^ RR is a multivariate method based on machine learning. Multivariate methods control for hidden biases, such as the vascular distribution of damage, that consistently distort lesion-deficit maps computed using voxel-wise univariate methods.^22^^,^^23^ These biases can displace inferred critical regions from their true locations in a manner opaque to replication. RR models allow us to predict behavioural variance based on structural features including volume and location (for additional detail of the procedure see Supplementary methods).

### Vascular territory control analysis

To test whether different vascular territories of the MCA were strongly associated with different PC scores, we clustered patients based on their lesion location. We computed the percentage overlap of each lesion with a mask of three vascular territories (deep branches, anterior-superior branch and posterior-inferior branch).^24^ We clustered these groups of lesions based on their location within the vascular territories and selected the optimal number of clusters through Silhouette and Davies Bouldin indexes. We used mixed measures ANOVA, with PC (PC1–PC3) as within-subjects factor and clusters as between-subjects factor, to test the interaction effect of PC scores and anatomical clusters. We performed the same analysis on the subset of patients of the Washington University dataset (*n* = 67).

### Data availability

All data reported in the present study are available from the authors and all the software and algorithms used in the present study are cited in the Material and methods.

## Results

### Participants

The study sample had a mean age of 69 years old. All patients were Caucasian. Most patients were male (53%). The majority had completed middle or high school in the Italian educational system (mean level of education: 10 years). The most commonly identified stroke risk factors were hypertension (64% of patients) followed by smoking, diabetes mellitus, atrial fibrillation and coronary artery disease (Supplementary Table 1, Demographics and Clinical Characteristics).

In terms of stroke-related variables, the study sample presented a mean NIH score of 7.1 ± 5.6 on admission, while the NIH score at the time of testing was 3.2 ± 2.9. The NIH score used for the analysis was the one collected at the time of neuropsychological testing. Motor impairment was the most common deficit (90% of patients), followed by aphasia (34%), and neglect (20%). The aetiology of most strokes (89%) was ischaemic while 11% were haemorrhagic. Slightly less than half of the ischaemic patients underwent acute stroke treatment (42%) (Supplementary Table 2. Acute reperfusion therapy details). Finally, 44% of patients presented left hemisphere damage, 40% right hemisphere damage, 7.5% infratentorial lesions and 9% had clinical deficits without lesions on neuroimaging scans (Supplementary Table 3).

### Anatomy

**Figure 1:**
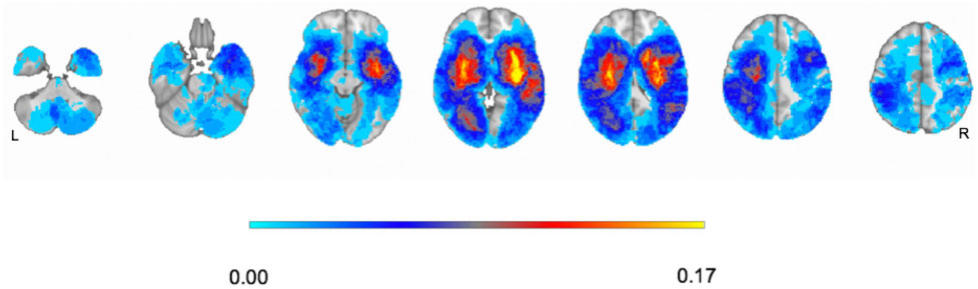
**Lesion topography.** Overlay of damage in atlas space (*n* = 164). The colour bar represents the percentage of lesions affecting each voxel (anatomical view).

To generate a precise description of stroke topography, we implemented a voxel-wise analysis of lesions. Figure 1 shows an overlay map of all segmented lesions normalized to a standardized brain atlas.^25^ The segmented lesions included: 79 subjects with left hemisphere lesions, 71 subjects with right hemisphere lesions and 14 subjects with cerebellum or brainstem lesions. Sixteen subjects presented negative MRI/CT scans for acute events. Stroke topography was predominantly subcortical and concentrated in the basal ganglia, central white matter and thalamus. Cortical lesions predominantly occurred in the MCA territory. Specifically, 10% of lesions exclusively affected the cerebral cortex, 22% damaged subcortical structures, while 65% were cortico-subcortical lesions (Supplementary Tables 3 and 4). The structural damage in our study was similar to the topography of recent studies on prospective clinical samples.^6^^,^^26^^,^^27^

### Behavioural PCA

A PCA was run on the OCS and NIHSS subtest scores to reduce the number of variables and identify hidden factors that capture behavioural variability. Most scores showed a long tail distribution with most patients having a peak near zero with a long positive tail consistent with varying degree of deficit. While the identification of many components would be consistent with the existence of many distinct behavioural syndromes, the discovery of a small number of components is consistent with correlated deficits across functional domains. The PCA was run on 158 subjects with a complete dataset including all NIHSS and OCS scores (88% of the enrolled patients; 22 patients were not able to complete the assessment due to fatigue or underlying comorbities).

**Figure 2:**
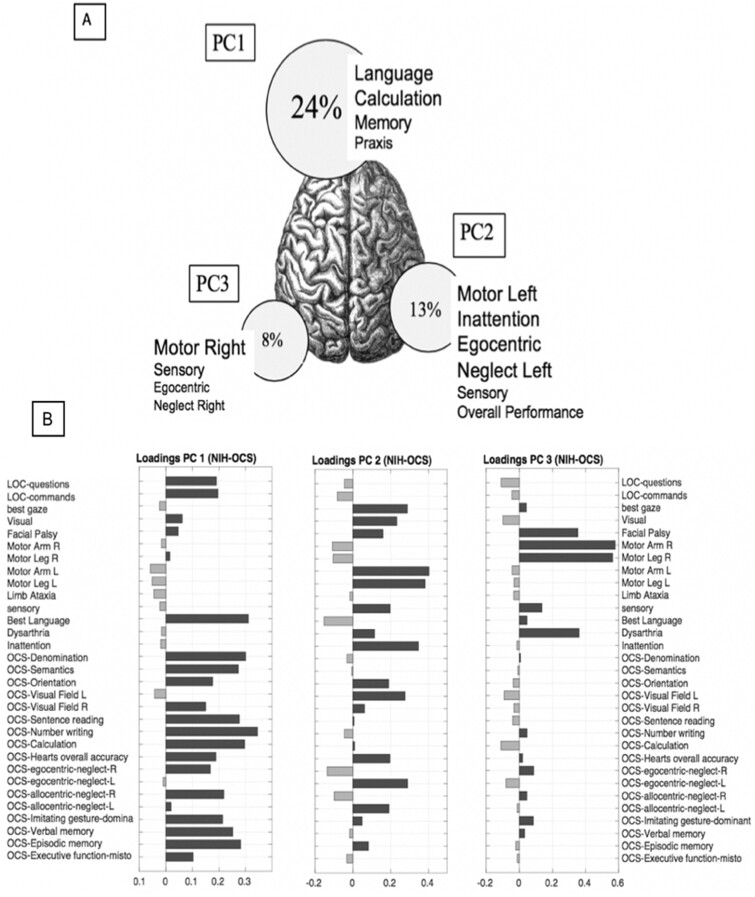
**Behaviour factor analysis.** (**A**) The percentage of variance explained by each Principal Component is proportional to the diameter of each circle. The position of each circle on the brain atlas represents the Principal Component’s lateralization. Finally, circles are labelled with the main subtests underlying each PC, the font size reflects the relative role of each loading. (**B**) Each table graphically shows single loadings of OCS and NIHSS subtests (on the right) for each PC. Black bars represent positive correlation, while grey bars represent negative correlation.

Three PCs accounted for nearly 50% of the behavioural variance (Fig. 2). PC1 accounted for 23.5% of the variance, PC2 for 14% of the variance and PC3 for 7.5% of the variance. This structure is represented in Fig. 2A where the size of each circle is proportional to the percentage of variance explained by each factor across subjects. Figure 2B shows the loadings for each score (see Supplementary Fig. 3 for non-rotated PCA loadings results). Positive loadings indicate lower performance, while negative loadings indicate higher performance. PC1 loaded on language, memory, calculation, apraxia and allocentric neglect. PC2 loaded on left side motor, visual, left egocentric neglect and overall performance deficits. PC3 loaded on right side motor deficits.

**Figure 3:**
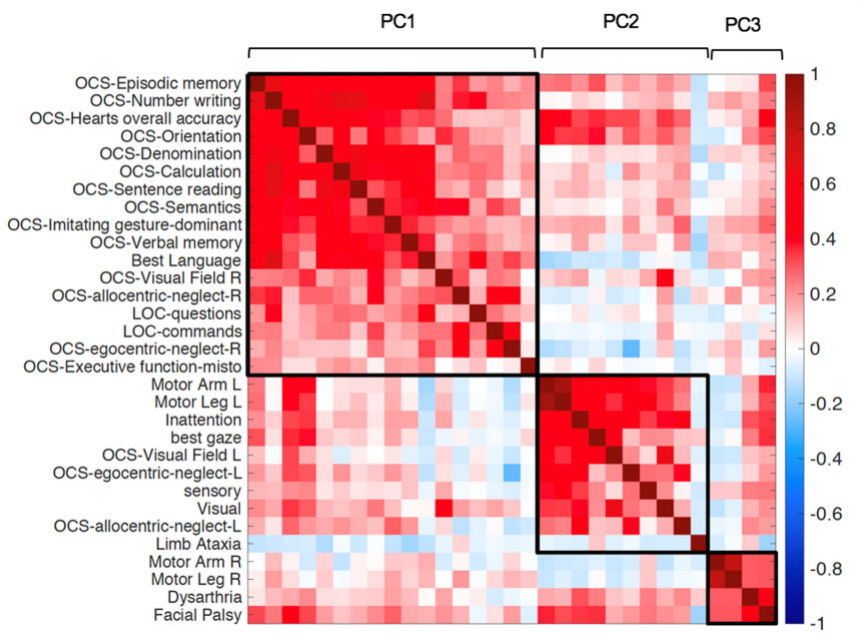
**Correlation matrix of behavioural subtests.** The colour bar represents Pearson r-values. Each square corresponds to the variables identified through the PCA analysis (i.e. PC1, PC2, PC3).

The correlation among behavioural scores was also examined through a correlation matrix (Fig. 3). A ‘block’ structure along the diagonal indicates correlation among different tests. Consistently with PC1, there was a robust correlation between language, calculation, praxis, verbal and spatial memory tasks, and right allocentric neglect. Left motor deficits correlated with left visual field and left egocentric and allocentric neglect (PC2), while right motor deficits formed a separate cluster (PC3). Interestingly, some tests show positive correlation across two components. For instance, the OCS Heart overall accuracy, a test of general performance, and the OCS orientation were common to PC1 and PC2; dysarthria and face palsy, which were not computed separately for left versus right body/field, loaded on both left and right hemisphere-specific components.

In summary, this analysis identified three main sets of correlated behavioural deficits: one cognitive related to language, calculation, praxis, and memory deficits, and two contra-lesional motor-attention components. General performance influenced both the cognitive and left motor-attention component.

### RR behaviour to anatomy

**Figure 4:**
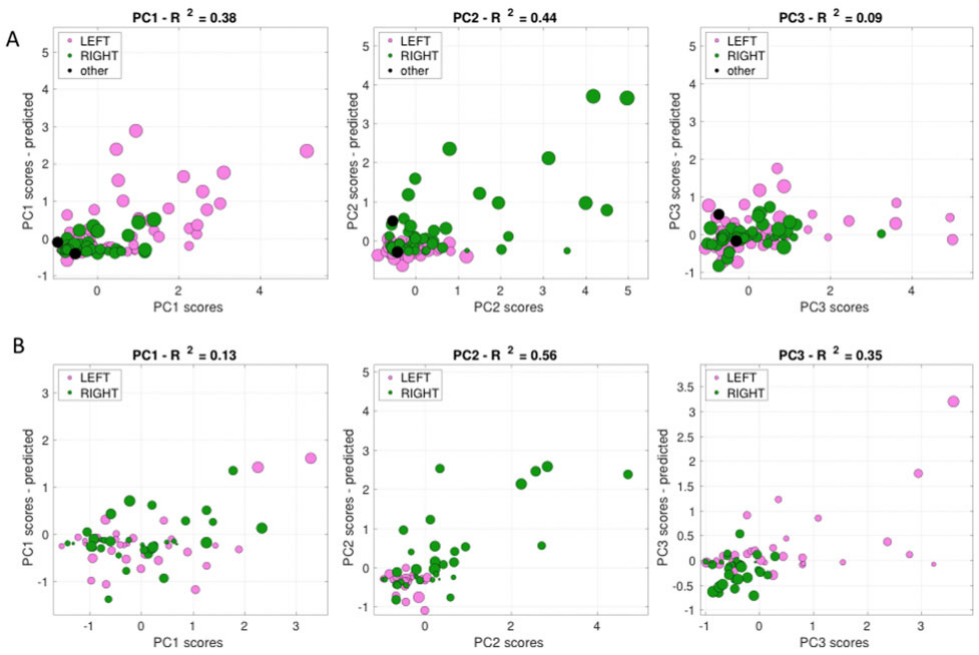
**Ridge regression scatter plots.** Factor scores for right (in green) and left (in red) lesions (in black for midline lesions). The diameter of each coloured circle is proportional to the lesions volume. Each lesion is associated to three Principal Component values: on the *X*-axis empirically measured behavioural scores, on the *Y*-axis the estimated behavioural scores of our model. If the lesion location is a good predictor then the relationship between empirical and model scores are linearly related. (**A**) are calculated on the University of Padua data, while (**B**) are calculated on the Washington University data.

To study the relationship between structural damage and behavioural impairment, we applied a RR model. The analysis was conducted on subjects (*n* = 148) that included both behavioural and neuroimaging data. Figure 4A shows the scatter plots of the empirically measured behavioural scores versus the estimated behavioural scores from the RR model based on the lesion anatomy. Essentially, the model predicts the best fitting behavioural scores from the distribution of lesioned voxels across patients. Each dot represents a subject, and the size of each dot is scaled by the lesion volume. The model explained different levels of variance for each factor score: PC1: 38%; PC2: 44%; and, PC3: 9%, respectively.

**Figure 5:**
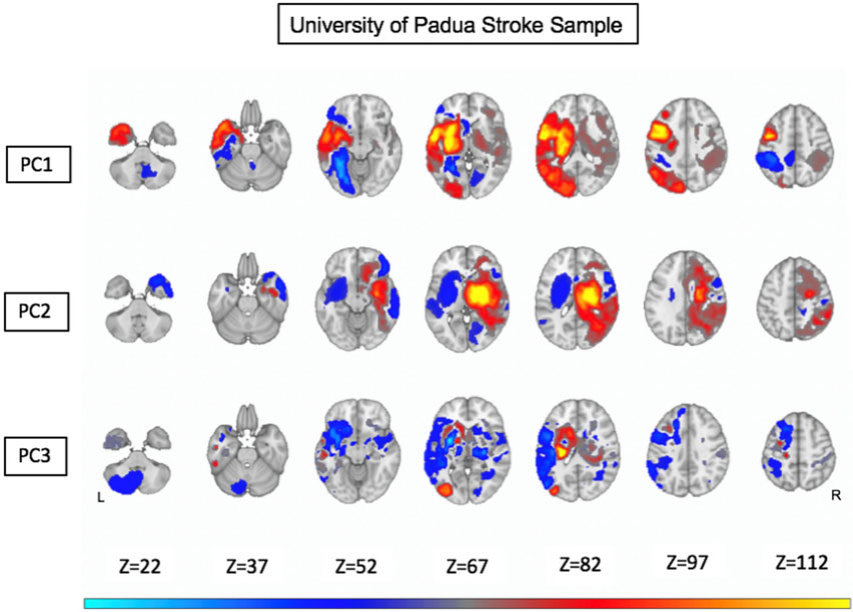
**Ridge regression maps from the University of Padova sample.** Warm colours represent positive correlation between anatomical voxels and high PC values (i.e. high level of impairment in the corresponding domains). Cold colours represent negative correlation between anatomical voxels and high PC values. Anatomical overlay maps are shown for PC1, PC2 and PC3 scores, respectively [after Gaussian smoothing (variance = 1) and scaling within $\left[-1,+1\right]$; weights lower than 0.05 in absolute values are not shown].

Figure 5 shows the maps of the most predictive anatomical structures (weights of the RR) associated with each PC scores. The anatomical description goes from the dorsal to the ventral slices, and from the anterior to the posterior direction. The orange/yellow colour scale indicates damaged voxels associated with low performance, whereas the blue/teal colour scale indicates damaged voxels associated with high performance. Here, we focus on anatomical regions associated with low performance. Low performance on language, memory, calculation and praxis (PC1) correlated with damage of the left superior and middle frontal gyrus, left inferior parietal and underlying white matter, left occipital dorsal, left inferior frontal gyrus/insula and underlying white matter, left putamen and caudate, left thalamus, and left anterior middle and inferior temporal gyrus. Left motor and attention deficits (PC2) correlated with damage of the right superior, middle, and precentral gyrus, right superior and inferior parietal regions, right corona radiata and internal capsule, right caudate, putamen, and thalamus, and right superior and middle temporal gyrus, right orbitofrontal gyrus. Finally, low scores on right motor and attention deficits (PC3) localized to damage of the left caudate, putamen, and internal capsule, left thalamus, and left lateral occipital cortex.

### Validation: WU cohort

To test the external validity of our predictions, we applied the same analysis to the behavioural scores of the WU cohort.^6^ The St. Louis WU cohort includes *n* = 132 first-time stroke patients prospectively enrolled with the same criteria as this study; the behavioural battery takes 2.5 h, and includes 44 scores in 7 domains (motor, visual, language, spatial attention, general performance, verbal and spatial memory). A PCA on the behavioural scores also yielded three components (PC1–3) that explained 49% of the variance, which loaded on similar functional domains. PC1 (22.5%) loaded on language and verbal/spatial memory; PC2 (15%) on left motor, left visuospatial neglect, general performance and spatial memory; PC3 (11.4%) on right motor and right spatial neglect^6^ (Supplementary Fig. 4). A RR model explained different levels of variance for each factor (PC1: 13%, PC2: 56% and PC3: 35%, respectively) (Fig. 4B).

**Figure 6:**
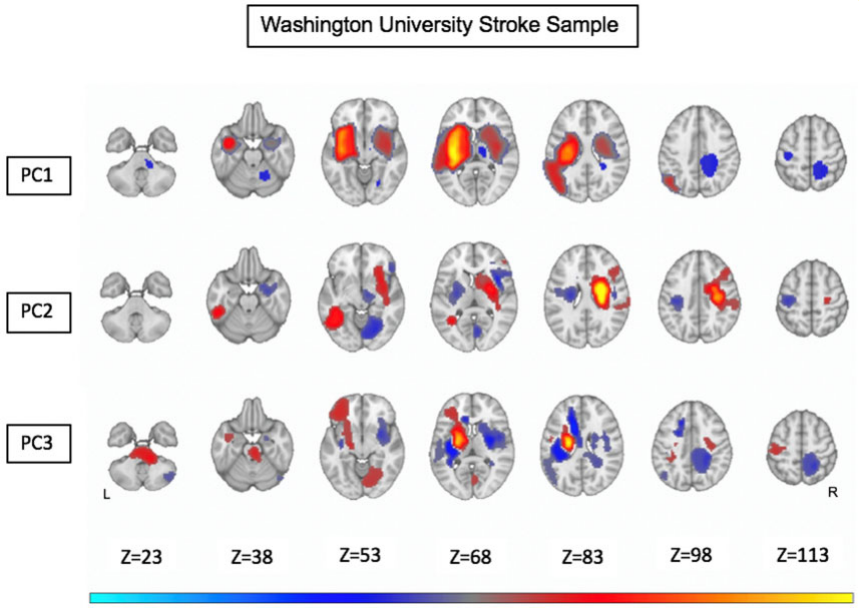
**Ridge regression maps from the Washington University sample.** Warm colours represent positive correlation between anatomical voxels and high PC values (i.e. high level of impairment in the corresponding domains). Cold colours represent negative correlation between anatomical voxels and high PC values. Anatomical overlay maps are shown for PC1, PC2 and PC3 scores, respectively [after Gaussian smoothing (variance = 1) and scaling within $\left[-1,+1\right]$; weights lower than 0.05 in absolute values are not shown].

The weights of the RR identified regions of the brain whose damage mostly contributed to the different PC scores. Low performance on language, memory, calculation and praxis (PC1) correlated with damage of several left hemisphere regions: left precentral white matter, left inferior parietal and underlying white matter, left insula and inferior frontal gyrus, left caudate, putamen, and thalamus, left anterior and middle temporal gyrus. This map contained also right hemisphere regions including right precentral white matter, right caudate, putamen, and thalamus, right insula, right anterior temporal gyrus. Left motor and attention deficits (PC2) localized to the right precentral cortex and underlying corona radiata, right caudate, putamen and internal capsule. A significant region was also in the left middle temporal gyrus. Finally, low scores on right motor and attention deficits (PC3) scores correlated with damage to the left precentral gyrus and underlying corona radiata, left internal capsule, putamen, and thalamus, left anterior inferior frontal gyrus (Fig. 6).

For each PC, we evaluated the spatial correlation between the maps obtained with our data and the maps obtained with the WU dataset, after having resampled both maps in the same space of the WU data. For PC1 we obtained a correlation *r* = 0.66 ($P<10^{-5}$); for PC2 *r* = 0.35 ($P<10^{-5}$); for PC3 *r* = 0.11 ($P<10^{-5}$). In general, the topography of damage related to the main axes of behavioural impairment was consistent between both samples of stroke patients.

### Control for vascular distribution

We tested whether lesions in different vascular territories [e.g. (deep vs. superficial branches of MCA] cause different profiles of deficits based on the PC behavioural scores. We clustered patients based on their lesion location. Cluster 1 included patients with lesions in deep, antero-superior and postero-inferior MCA branches. Cluster 2 included patients with lesions in the deep branches of MCA. Cluster 3 included patients with lesions in the postero-inferior branch of MCA and cluster 4 lesions in the antero-superior branch of MCA (Supplementary Fig. 5A). The mixed measures ANOVA, with PCs as within-subjects factor and clusters as between-subjects factor found a significant global interaction effect (*F* = 2.48, *P* = 0.024), but no significant main effects. The interaction effect was driven by different patterns of PC scores between cluster 2 and 3 (*F* = 5.008, *P* = 0.04, Bonferroni corrected for 6 multiple comparisons). This interaction shows that PC1 scores are stronger in cluster 3 given the cortical/perisylvian distribution, while PC3 scores are higher in cluster 2 given the subcortical location (Supplementary Fig. 5B). The same analysis on the subset of patients of the Washington University dataset (*n* = 67) found no interaction effect between PC scores and vascular clusters (Supplementary Fig. 5C).

## Discussion

This study investigated whether previously described^6^ groups of correlated deficits describing post-stroke behavioural variability could be validated using a different population and a different neuropsychological battery. Furthermore, we studied whether a simplified neurological and psychological assessment could be used as a sensitive measure of these axes of impairment. Finally, the topography of stroke lesions was analysed to map the relationship between structural damage and behavioural biomarkers.

The behaviour factor analysis showed a strong correlation between deficits across domains. Three factors explained ≈50% of the variance with Factor 1 loading on functions that are traditionally associated with the left hemisphere: language, verbal memory, calculation and praxis. However, on Factor 1, we also found visual episodic memory and general performance, functions that are typically associated with the right hemisphere.

In the language domain, subtests evaluated the level of speech production, auditory comprehension, reading capacities and general performance. All language tasks loaded under PC1 showing correlation that accounted for ≅24% of the whole variability of scores across subjects with no clear separation in the traditional aphasia syndromes, e.g. (Broca, Wernicke). This correlation among language deficits/syndromes is comparable to the St. Louis WU cohort: their PC1 accounted for 22.5% of variance.^6^ Interestingly, the Padova PC1 also includes tasks for number processing abilities (Number Writing and Calculation). Number processing is traditionally associated with lesions of the parietal lobe, especially the left parietal (even though an association with right parietal cortex was recently described by Semenza et al).^28^ PC1 also loaded on praxis, a left fronto-parietal function.^29^ Finally, PC1 also loaded on verbal and visual memory, similarly to what we find in the St. Louis battery. Interestingly, the Padova PC1 also includes correlation with right visual neglect and general performance. Overall, then, both Padova and St. Louis PC1 capture correlated deficits in many traditional left hemisphere functions (language, calculation, praxis, verbal memory), but also right hemisphere functions (general performance and visual memory).

PC2 and PC3 capture in both batteries, respectively, left and right motor deficits. The ranking in variance explained is also similar, first left (PC2) then right (PC3) motor deficits. Interestingly, in the motor domain, we do not see the traditional vascular syndromes (e.g. middle vs. anterior cerebral vs. subcortical), but correlated deficits of both upper and lower extremity motor function. This is consistent with prior PCA studies on the NIHSS^4^^,^^5^ and Corbetta et al.^6^ While traditional neuropsychological and neurophysiological investigations differentiate between sensory versus memory driven movements, and reaching versus grasping,^30–32^ more recent studies emphasize the correlation among different kinds of ecological movements, and the low dimensionality of movements in terms of kinematic analysis, EMG activation, and even responses in motor cortex. A reaching movement for instance will require coordinated movements of shoulder, arm, elbow, wrist and fingers that occur together in patterns of neural activation (synergies).^33–36^

The OCS does a good job in separating deficits of attention. General performance captured by the overall detection score on the Heart task loads on both PC1 and PC2. PC2 also captures left visual neglect, both egocentric, i.e. cantered on the body midline, and allocentric, i.e. cantered on the midline of objects, consistently with the syndrome of hemi-spatial neglect.^37^ Interestingly, right allocentric neglect loads on PC1 consistent with the observation that this form of neglect is better conceptualized as a left hemisphere object agnosia.^37^^,^^38^ Both Padova PC2 and PC3 are highly similar in structure to St. Louis, despite differences in the neuropsychological tests used.

Overall, then our study essentially replicates Corbetta et al. demonstrating that at the population level this low-dimensional structure of behavioural impairment is specific to stroke irrespective of population, time of testing (5 days Padova, 2 weeks, 3–12 months St. Louis) and other non-specific factors (i.e. variability in performance, low motivation, anxiety or depression) potentially present at the acute phase.

While the St. Louis battery takes between 1 and ½ and 2 h being structured in 44 different scores covering multiple domains: motor, language, memory, attention,^6^ the neurobehavioural battery in this study was shorter to administer. The OCS, a validated tool for cognitive assessment in stroke,^12^ can be readily administered in approximately 10 min. It has shown high levels of inclusivity, reliability, convergent and divergent validity between subtests and other cognitive tests, such as MOCA, BDAE, Wechsler.^12^ It has been validated in several countries^12^^,^^39–44^ stratified for age, gender and education level. Recent studies have demonstrated high levels of sensitivity in detecting stroke-specific cognitive impairments even in mild stroke.^45^ The NIH stroke scale was designed to be standardized, repeatable, and usable in large multi-centre clinical trials.^46^ Clinical researchers have widely accepted this scale due to high levels of inter-examiner and test-retest score consistency.^47^ In addition, it has been repeatedly validated as an excellent predictor for patient outcome.^14^ While previous studies on the factor structure of the sole NIH stroke scale^4^^,^^5^ have identified two factors, one for each hemisphere, this study combining the NIHSS and OCS replicates the 3-factor structure identified in Corbetta et al.^6^ This implies that to capture cognitive impairment the NIHSS should be integrated with a more sensitive cognitive screen.

Importantly, we found that the combination of NIHSS and OCS had an excellent level of compliance. We were able to administer all subtests at 5 days to 88% of enrolled patients, against 51% of enrolled patients at 2 weeks on the St. Louis battery. It remains to be seen if the NIHSS/OCS battery will be sensitive to recovery similarly to the St. Louis battery.^7^

It should be underscored, however, that in both datasets a significant amount of behavioural variance (∼50%) was not described by our data reduction approach and the effect size of PC3 was in general quite small. Where does the rest of the behavioural variance in stroke go? One possibility would be to add more patients hoping that as more lesions sample-specific locations in the brain, more specific patterns of behaviour will emerge. This is possible, even though we currently feel this is unlikely. In Padova, we carried out a preliminary analysis with *n* = 100 individuals (as compared to *n* = 180 in the final analysis), and we obtained the same three factors explaining about the same amount of variance. In St. Louis, we more than doubled the subjects by running PCA on domain-specific components obtained on the maximum number of patients, and the variance accounted increased only by 15%.

So how can we improve our post-stroke behavioural description? It is possible that the percentage will increase as some other important cognitive domains are included (i.e. emotion, decision making, social cognition, theory of mind). In particular, the identified axes of behavioural impairment are similar to the main behavioural axes described in healthy subjects when considering the brain’s functional lateralization through fMRI meta-analytic data. Karolis et al. showed that four axes (i.e. symbolic communication, perception/action, emotion and decision-making) could summarize the entire architecture of the brain’s lateralization of function.^48^ Karolis results could provide a physiological counterpart to the identified post-stroke behavioural biomarkers, but it suggests that at least two additional cognitive domains (emotion and decision-making) shall be added to our short battery to provide a comprehensive behavioural profile of stroke patients.

When considering structural damage, our study demonstrated stroke topography was predominantly subcortical, with a paucity of cortical lesions. Lesions were extremely heterogeneous in volume, including both lacunar and hemispheric strokes. All vascular territories were involved, with the MCA predominantly affected in accordance with well-established stroke literature.^20^^,^^49^ Cortical areas were exclusively affected in only 10% of patients, in agreement with data from other prospective clinical sample studies on acute stroke patients.^6^^,^^26^^,^^27^ A significant portion of the sample (42%) underwent acute reperfusion therapy. Demographic factors and differences in clinical characteristics did not likely bias topography, especially as strong factors associated with subcortical damage (such as hypertension, diabetes type II and hemorrhagic strokes) were actually slightly less represented in our sample in comparison with other consecutive sample studies.^50^ Once again, these results emphasize how stroke (both in topography and symptoms) should be better conceptualized as a subcortical disease with secondary impact on white matter pathways and cortico-cortical and cortico-subcortical functional interactions.^51^

Finally, we investigated the relationship between behavioural biomarkers and stroke topography through a multivariate machine learning method. While previous studies have performed factor analysis to identify neural structures of pre-conceived and distinct functional domains (i.e. language, motor, memory, attention),^6^^,^^52^ the PC scores of our subjects derive from statistical correlation alone and bypass the need of, often overlapping, behavioural classifications of deficits to map biomarkers. Most importantly, this completely data-driven approach provides topographical correlates regarding post-stroke multi-domain impairment. High PC1 (language, memory, calculation, praxis) scores mainly correlated with damage of left cortico-subcortical regions; high PC2 scores (left motor and visual attention) with damage of right cortico-subcortical regions; high PC3 scores (right motor) with damage of left subcortical regions. The resulting maps are consistent with those obtained by running the same analysis on the St. Louis data set.^6^ These results show that behavioural impairment following a stroke can be reliably related to lesion location and raise several interesting considerations.

Firstly, our anatomical correlates do not correspond to precise vascular territories. In fact, we tested if different vascular territories of the MCA were associated with different PC scores and found no clear association in either dataset (Supplementary Fig. 5).

Secondly, our structural models were able to explain only low-medium levels of variance for our components (PC1 = 38%, PC2 = 44% and PC3 = 9%, respectively). These results agree with recent studies that show both lesion location and functional network impairment account for behavioural variance.^53^ Moreover, while sensorimotor deficits are more precisely predicted by structural variables, cognitive deficits depend more on multi-network functional connectivity alterations.^8^^,^^54^ As our components derive from statistical correlation alone, we believe models that include pathophysiological information (such as white matter disconnection and f-MRI analysis), could provide better results in predicting our behavioural biomarkers.^53^^,^^55^^,^^56^

In conclusion, this study demonstrated a low-dimensional structure of neurological deficits following stroke using a combination of clinically applicable batteries. Neurological deficits post-focal lesions are more accurately described by correlated deficit components rather than the collection of individual syndromes as in traditional neurological teaching. We identified a few factors that showed consistency across different populations and different neurobehavioural batteries. The associated lesion topography of the identified components was also robust.

The identified biomarkers are therefore sensitive measures of behavioural impairment when investigating the epidemiology, genetics, or pathophysiology of stroke. They should also be employed to assess the efficacy at the population level of novel acute or chronic interventions.

## Supplementary material

Supplementary material is available at *Brain Communications* online.

## Supplementary Material

fcab119_Supplementary_Data

## Abbreviations

MCA =: middle cerebral artery
NIHSS =: National Institutes of Health Stroke Scale
OCS =: Oxford Cognitive Screen
PC =: principal component
PCA =: principal component analysis
RR =: ridge regression
WU =: Washington University St. Louis
