# Supplementary material for: A low-dimensional structure of neurological impairment in stroke
Source: Brain Commun. 2021 Jun 3;3(2):fcab119. doi: 10.1093/braincomms/fcab119 (PMC8204367; doi:10.1093/braincomms/fcab119)
Supplement: fcab119_Supplementary_Data [file fcab119_supplementary_data.zip › Original Submission.pdf]

OXFORD UNIVERSITY PRESS | Brain Communications

A low dimensional structure of neurological impairment in stroke

Journal:	*Brain Communications*
Manuscript ID	BRAINCOM-2020-342
Manuscript Type:	Original Article
Date Submitted by the Author:	09-Nov-2020
Complete List of Authors:	Bisogno, Antonio Luigi; University of Padova Faculty of Medicine and Surgery, Department of Neuroscience<br>Favaretto, Chiara; University of Padua, Padova Neuroscience Center<br>Zangrossi, Andrea; University of Padua, Padova Neuroscience Center<br>Monai, Elena; University of Padova Faculty of Medicine and Surgery, Department of Neuroscience<br>Facchini, Silvia; University of Padua, Deparment of Neuroscience<br>De Pellegrin, Serena; Azienda Ospedaliera di Padova, Department of Neuroscience<br>Pini, Lorenzo; Università degli Studi di Padova, Padova Neuroscience Center<br>Castellaro, Marco; Department of Information Engineering (DEI), University of Padova,<br>Basile, Anna Maria; Azienda Ospedale Universita Padova, St. Antonio Hospital, Neurology Department<br>Baracchini, Claudio; Azienda Ospedaliera di Padova, Department of Neuroscience<br>Corbetta, Maurizio; University of Padua, Deparment of Neuroscience; Washington University in Saint Louis School of Medicine, ; Venetian Institute of Molecular Medicine; VIMM, Padova Neuroscience Center
Keywords:	

SCHOLARONE™
Manuscripts

A low dimensional structure of neurological impairment in stroke

Running head: Correlated behavioral deficits post-stroke

Antonio L. Bisogno[1,2], Chiara Favaretto[1,2], Andrea Zangrossi[1,2], Elena Monai[1,2,3], Silvia Facchini[1,2], Serena De Pellegrin[3], Lorenzo Pini[2], Marco Castellaro[2,4], Anna Maria Basile[3], Claudio Baracchini[3], Maurizio Corbetta[1,2,3,5,‡]

[1] Department of Neuroscience, University of Padova, Padova, Italy; [2] Padova Neuroscience Center (PNC), University of Padova, Padova, Italy; [3]Azienda Ospedaliera Università di Padova; [4] Department of Information Engineering, University of Padova, Italy; [5] Department of Neurology, Radiology, Neuroscience Washington University School of Medicine, Saint Louis, MO, USA

‡ Corresponding Author: Clinica Neurologica, Dipartimento di Neuroscienze, Universita' di Padova, via Giustiniani 2, 37128 Padova, Italy.

For Review Only

ABSTRACT

Neurological deficits following stroke are traditionally described as syndromes related to damage of a specific area or vascular territory. Recent studies indicate that post-stroke neurological impairment is best described by a few groups of correlated deficits across different domains that relate to the dysfunction of distributed brain networks. To examine the reproducibility and specificity of this structure, we prospectively studied first-time stroke patients (n=237) using a bedside, clinically applicable, neuropsychological assessment and compared the behavioral and anatomical results with those obtained from a different prospective cohort studied with an extensive neuropsychological battery.

The behavioral assessment at one-week post stroke included the Oxford Cognitive Screen (OCS) and the National Institutes of Health Stroke Scale (NIHSS). A principal component analysis was used to reduce variables and describe behavioral variance across patients. Lesions were manually segmented on structural scans. The relationship between anatomy and behavior was analyzed using multivariate regression models.

Three principal components (PC) explained ≈50% of the behavioral variance across subjects. PC1 loaded on language, calculation, praxis, right side neglect, and memory deficits; PC2 loaded on left motor, visual, and spatial neglect deficits; PC3 loaded on right motor deficits. These components matched those obtained with a more extensive battery. The underlying lesion anatomy was also similar.

Neurological deficits following stroke are correlated in a low dimensional structure of impairment, related neither to the damage of a specific area or vascular territory. Rather they reflect widespread network impairment caused by focal lesions. These factors showed consistency across different populations, neurobehavioral batteries and, most importantly, can be described using a combination of clinically applicable batteries (NIHSS & OCS). They represent robust behavioral biomarkers for future stroke population studies.

For Review Only

Introduction

"You learn neurology stroke by stroke" C.M. Fisher (1961)

Neurologists traditionally classify behavioral syndromes based on damage of specific brain regions (e.g., Broca aphasia) or the vascular distribution of stroke (e.g., middle cerebral artery). When behavioral deficits are correlated the explanation is that adjacent cortical regions suffer from the injury, be it ischemia, as in right hemiplegia and Broca aphasia, or abnormal electrical activity, as in the Jacksonian march. (Broca, 1861; Jackson, 1863; CM, 1961) Dr. Fisher described more than 70 different syndromes caused by focal ischemia in his work .(CM, 1961)

However, recent work offers a different perspective showing that syndrome-based descriptions do not characterize behavioral deficits at the population level. For instance, the examination of samples of stroke patients with the National Institutes of Health Stroke Scale (NIHSS) identifies two factors: one for left and one for right hemisphere lesions, which split respectively in a cognitive and sensory-motor component, accounting for approximately 80% of behavioral variability across subjects. (Lyden *et al.*, 2004; Zandieh *et al.*, 2012)

Since cognitive deficits are only cursorily measured by the NIHSS, this simplified model may reflect a lack of sensitivity for impairment in multiple cognitive domains. However, a more recent analysis in a prospective sample of stroke patients (n=132), tested with an extensive neuropsychological battery (44 tests covering multiple domains: language, motor, vision, memory, attention) at Washington University (WU) in St. Louis, discovered that three deficit components account for the majority (65%) of variability in performance.(Corbetta *et al.*, 2015) These factors remained at three-twelve months post-stroke, tracked recovery, and could represent biomarkers of impairment. (Ramsey *et al.*, 2017) The first factor loaded on language, including deficits of language expression and comprehension, and memory, both verbal and spatial. The second and third factors loaded on the contralateral motor and visual attention deficits, i.e., left deficits for right lesions, and vice versa. Neither local damage or vascular distributions could account for the observed correlation of deficits in different domains. Instead, a strong relationship was observed with functional network damage measured with fMRI. (He *et al.*, 2007; Carter *et al.*, 2010; Baldassarre *et al.*, 2016; Siegel *et al.*, 2016)

The first aim of this study was to validate through a short and clinically applicable assessment the previously identified structure of impairment in a different population: Veneto, Italy. We used the Oxford Cognitive Screening (OCS), specially developed by the late psychologist Glyn Humphreys and colleagues to study post-stroke cognitive impairment.(Demeyere *et al.*, 2015, 2016) This test covers language, memory, attention, calculation, and praxis; it takes 10-15 minutes to be administered --against more than 2 hours for the WU battery-- and with the NIHSS may provide a

For Review Only

clinically suitable neurobehavioral assessment applicable in busy stroke units. The data were analyzed to find robust components of impairment that were correlated across patients. The results were then compared to those obtained by analyzing the independent dataset from WU in the same manner. The second aim was to examine the neuroanatomy of these factors using a multivariate machine learning approach. We related spatial patterns of damage to behavioral scores to find a lesion model that best accounted for the individual variability of scores. To replicate the neuroanatomy, we ran the same approach on the WU cohort.

Materials and methods

Study Sample

The recruitment covered 22 months, from December 2017 to October 2019, and occurred at the Stroke Unit and Clinica Neurologica of the Hospital of Padova (AOPD) and the Stroke Unit of the Ospedale S. Antonio Padova (OSA).

The inclusion criteria, same as in the WU cohort, included:

1. Age 18 or higher;
2. First symptomatic stroke, ischemic or hemorrhagic in etiology;
3. Up to two lacunes, clinically silent, less than 15 mm in size on CT scan;
4. Time of enrollment: < 2 weeks from stroke onset;
5. Awake, alert, and capable of participating in research.

Exclusion criteria included: (1) Previous stroke based on clinical imaging; (2) Multifocal strokes; (3) Inability to maintain wakefulness in the course of testing; (4) More than two asymptomatic lesions on CT scan; (5) Presence of central nervous system tumors; (6) History of dementia; (7) Previous central nervous system surgeries; (8) Schizophrenia, bipolar disorder, major depression, or other severe psychiatric conditions; (9) Other medical conditions that preclude active participation in research and may alter the interpretation of the behavioral/imaging studies; (10) Inability to provide consent; for severe aphasic patients informed consent next-of-kin gave informed consent. We screened a total of N=1080 charts, of which 324 met inclusion criteria and recruited N=237. **Supplementary Figure 1** describes the enrollment flowchart and shows reasons for lack of inclusion. **Supplementary Figure 2** shows the design of the study.

Clinical Data

Experienced neurologists examined all patients using the NIHSS(Muir *et al.*, 1996), which was administered on admission, on discharge and at the time of testing (within a week). The NIHSS includes 15 subtests: level of consciousness subtests, gaze and visual field deficits, facial palsy,

For Review Only

upper and lower motor deficits (right and left side), limb ataxia, sensory impairment, inattention, dysarthria and language deficits. The NIHSS scores at the time of testing were analyzed (**Supplementary Table 1** shows the subtests of the NIHSS). Also we recorded: demographics data, stroke risk factors, other neurological, psychological, or psychiatric conditions, familiarity for stroke, stroke subtype (hemorrhagic or ischemic), clinical presentation (See **Supplementary Table 2** for Demographics & Clinical sample characteristics).

Neuropsychological Battery

The OCS was administered the first week following stroke onset. The OCS is a brief tool -- 10-15 minutes long-- developed to describe acute cognitive impairment post-stroke.(Demeyere *et al.*, 2015) It is structured around five cognitive domains: Language, Praxis, Number Processing, Attention, and Memory, and consists of 10 individual subtests. In the language domain, Picture naming, Picture pointing, and Sentence reading subtests measure speech production, auditory comprehension, and reading, respectively. In the memory domain, verbal and spatial memory are examined separately through the Orientation, Recall and Recognition, and Episodic memory subtests. Number writing and calculation tasks evaluate number processing. An Imitating Meaningless Gestures test measures praxis. Finally, in the attention domain, the Broken Heart and Trail tests measure sustained attention, visuospatial lateralized attention (egocentric and allocentric), and executive functions. Visual fields are checked separately (**Supplementary Table 1** shows the subtests of the OCS)

MRI and CT lesions

MRI and/or CT scans were routinely performed on admission and follow up depending on clinical status. Lesions were manually segmented on structural MRI and CT scans using the ITK-snap imaging software system(Yushkevich *et al.*, 2006) and individually checked by a neurology resident and a board certified neurologist.(W.T. Longstreth, Teri A. Manolio, Alice Arnold, Gregory L. Burke, Nick Bryan, Charles A. Jungreis, Paul L. Enright, Daniel O'Leary, 1996; Wahlund *et al.*, 2001) CT and MRI segmented lesions were mapped on the MNI152 atlas using the Advanced Normalization Tools (ANTs).(Avants *et al.*, 2011) The FSL software was used to create the overlap of individual lesions on a standard brain atlas, producing an overlay map of all lesions.(Jenkinson *et al.*, 2012) Finally, to precisely describe stroke topography, lesions were mapped on the Harvard-Oxford cortical and subcortical structural masks.('Harvard-Oxford cortical and subcortical structural atlases included in FSL', n.d.) **Supplementary Table 4** provides a list of the anatomical regions

For Review Only

damaged by each lesion with its volume in voxels (2x2x2 mm), and ml. **Supplementary Table 5** shows the mean, std, and maximum % damage of each parcel affected.

Behavioral Analysis

The statistical analysis included the OCS subtests scores and the NIHSS individual scores**.** All subtests were normalized to their maximum values, and sign-inverted, such that the largest values corresponded to the most severe level of deficit. Only patients who participated to all task sets were included. After having z-scored the behavioral scores, we used a principal component analysis (PCA) to reduce the number of variables and describe the variability of behavioral deficits. Since many variables were expected to be correlated, an oblique rotation (PROMAX) was used (for completeness non-rotated PCAs and the corresponding anatomical maps were computed; see **Supplementary Figure 3).** As the oblique rotation is dependent on the number of selected components, we decided to be consistent with Corbetta et al.,(Corbetta *et al.*, 2015) and selected the first three Principal Components.

Moreover, a correlation matrix was computed to graphically visualize the strength of correlation between tests. Many subtests were at ceiling, with most subjects reaching maximum scores. Matlab R2018b was used for all statistical analysis.

For Review Only

Lesion-behavior Analysis

The analysis was run on our sample and a subset of patients of the WU cohort (n=67 had completed all tests of the battery). To relate behavioral deficits to lesions, we employed a ridge regression algorithm (RR)(Problems *et al.*, 1970). Ridge regression is a multivariate method based on machine learning. Multivariate methods control for hidden biases, such as the vascular distribution of damage, that consistently distort lesion-deficit maps computed using voxel-wise univariate methods(Phan TG, Chen J, Donnan G, Srikanth V, Wood A, 2010; Mah *et al.*, 2014). These biases can displace inferred critical regions from their true locations in a manner opaque to replication. Ridge regression models allow us to predict behavioral variance based on structural features including volume and location.

RR adds a L2-normalization term to the ordinary linear regression, in order to assign small coefficients to unimportant predictors, thus preventing data overfitting, and improving generalization for new data.

We used the binary matrix of voxels damage as predictor (for each subject and for each voxel, the entry of the matrix is set to 1 if the voxel is lesioned and 0 otherwise). Due to computational issues, instead of considering all 902,629 2-mm^3 voxels, we first applied a spatial PCA, and we used as regressors only the first N_p PCs, which explained at least the 95% of the variance. Besides resolving the dimensionality problem, the PCA step had also the purpose to transform the original binary matrix into a set of continuous predictors.

Thus, for each of the behavioral PCs, we estimated the model weights vector $\boldsymbol{\beta}$ as:

$$\boldsymbol{\beta} = \left(\boldsymbol{X}^{\mathrm{T}}\boldsymbol{X} + \lambda\boldsymbol{I}\right)^{-1}\boldsymbol{X}^{\mathrm{T}}\boldsymbol{y}\,,$$

where $\boldsymbol{X} \in \mathbb{R}^{N_s \times N_p}$ is the predictors matrix (N_s is the number of subjects and N_p is the number of selected spatial PCs), after z-scoring w.r.t. the whole matrix; $\boldsymbol{X}^{\mathrm{T}} \in \mathbb{R}^{N_p \times N_s}$ is the transpose of $\boldsymbol{X}$, $\boldsymbol{y} \in \mathbb{R}^{N_s}$ is the vector of the outcome variable to be predicted (i.e. the selected behavioral PC score, after z-scoring), $\boldsymbol{I} \in \mathbb{R}^{N_p \times N_p}$ is the identity matrix of dimension N_p, and $\lambda \in \mathbb{R}$ is the regularization parameter, optimized as follows.

For each of the three RR models, the regularization parameter λ was optimized by identifying a value within $[10^{-5},\ 10^5]$, with 200 logarithmic steps. For each of these 200 values of λ, each RR model was trained and tested using a leave-one-out cross validation loop (LOOCV), which uses $N_s - 1$ training data to estimate the model weights and applies them to the left-out patient to predict his behavioral score. The optimal λ (λ_{opt}) value was the one that minimized the prediction error over the training set, and the predictions obtained with λ_{opt} were considered as the model predictors $\hat{\boldsymbol{y}}$.

Model accuracy was assessed through R^2:

$$R^2 = 1 - \frac{\sum_{i=1}^{N_s}(y_i - \hat{y}_i)^2}{\sum_{i=1}^{N_s}(y_i - \bar{y})^2},\ \text{where}\ \bar{y} = \frac{1}{N_s}\sum_{i=1}^{N_s} y_i,$$

where y_i represent the i-th element of vector $\boldsymbol{y}$.

The statistical significance was estimated through a permutation test, with $N = 10{,}000$ iterations. For each iteration, behavioral scores were randomly permuted across subjects, and the LOOCV with λ optimization was used to fit the RR model to the randomized scored. The p-value for the observed R^2 was defined as the probability of the R^2 of the randomized dataset to be larger than the observed R^2. Only models with p-values less than 0.05 were considered statistically able to predict the behavioral scores.

To obtain the optimal set of RR model weights $\boldsymbol{\beta}$, the weights obtained for each LOOCV loop at λ_{opt} were averaged across the N_s loops. The distribution of weights obtained with the permutation test was used as null distribution to select statistically significant weights. Only the β_i's that fall at the left or right ends (2.5%) of the tails of the distribution were considered significant. These selected weights

were projected to the brain to display a map of the most predictive lesioned voxels. Finally, Gaussian smoothing (variance = 1) and scaling within $[-1, +1]$ was applied on the maps. Weights lower than 0.05 in absolute values were not shown.

Data availability

All data reported in the present study are available to the authors and all the software and algorithms used in the present study are cited in the material and methods.

Results

Participants

Subjects (n=237) with a first symptomatic stroke, ischemic or hemorrhagic, were prospectively recruited, with n=180 meeting post-enrollment inclusion criteria (**Supplementary Figure 1**). Subjects were evaluated with a neurobehavioral battery at the acute phase (5±3.3 days post stroke). The behavioral battery included the Oxford Cognitive Screen (OCS)19 and the National Institute of Health Stroke Scale (NIHSS)18. We collected structural imaging (MRI and/or CT scan) that is routinely performed for each subject at 5±4 days post-stroke. **Supplementary Figure 2** illustrates the design of the study.

The study sample had a mean age of 69 years old. All patients were Caucasian. Most patients were male (53%). The majority had completed middle or high school in the Italian educational system (mean level of education: 10 years). The most commonly identified stroke risk factors were hypertension (64% of patients) followed by smoking, diabetes mellitus, atrial fibrillation, and coronary artery disease (**Supplementary Table 2** Demographics & Clinical Characteristics).

In terms of stroke-related variables, the study sample presented a mean NIH score of 7.1±5.6 on admission, while the NIH score at the time of testing was 3.2±2.9. The NIH score used for the analysis was the one collected at the time of neuropsychological testing. Motor impairment was the most common deficit (90% of patients), followed by aphasia (34%), and neglect (20%). The etiology of most strokes (89%) was ischemic while 11% were hemorrhagic. Slightly less than half of the ischemic patients underwent acute stroke treatment (42%) (see **Supplementary Table 3** Acute reperfusion therapy details). Finally, 44% of patients presented left hemisphere damage, 40% right hemisphere damage, 7.5% infratentorial lesions, and 9% had clinical deficits without lesions on neuroimaging scans (**Supplementary Table 2**).

Anatomy

For Review Only

To generate a precise description of stroke topography, we implemented a voxel-wise analysis of lesions. **Figure 1** shows an overlay map of all segmented lesions normalized to a standardized brain atlas.(Rorden *et al.*, 2012) The segmented lesions included: 79 subjects with left hemisphere lesions, 71 subjects with right hemisphere lesions, and 14 subjects with cerebellum or brainstem lesions. Sixteen subjects presented negative MRI/CT scans for acute events (**Supplementary Table 4** for neuroimaging details). Stroke topography was predominantly subcortical and concentrated in the basal ganglia, central white matter, and thalamus. Cortical lesions predominantly occurred in the middle cerebral artery territory. Specifically, 10% of lesions exclusively affected the cerebral cortex, 22% damaged subcortical structures, while 65% were cortico-subcortical lesions (**Supplementary Table 4 & Supplementary Table 5** for Neuroimaging details). The structural damage in our study was similar to the topography of recent studies on prospective clinical samples.(Kang *et al.*, 2003; Wessels *et al.*, 2006; Corbetta *et al.*, 2015)

Behavioral Principal Component Analysis

A principle component analysis (PCA) was run on the OCS and NIHSS subtest scores to reduce the number of variables and identify hidden factors that capture behavioral variability. Most scores showed a long tail distribution with most patients having a peak near zero with a long positive tail consistent with varying degree of deficit. While the identification of many components would be consistent with the existence of many distinct behavioral syndromes, the discovery of a small number of components is consistent with correlated deficits across functional domains. The PCA was run on 158 subjects with a complete dataset including all NIHSS and OCS scores (88% of the enrolled patients; 22 patients were not able to complete the assessment due to fatigue or underlying comorbities).

Three principal components (PC) accounted for nearly 50% of the behavioral variance (**Figure 2**). Positive loadings indicate lower performance, while negative loadings indicate higher performance. PC1 loaded on language, memory, calculation, apraxia, and allocentric neglect [OCS_Denomination, OCS_semantics, OCS_Orientation OCS_sentence reading, OCS_Number Writing, OCS_Calculation, OCS_Imitating Gesture, OCS_Episodic Memory, OCS_Verbal Memory, OCS_allocentric neglect, NIH Best Language]. PC2 loaded on left side motor, visual, left egocentric neglect, and overall performance deficits [OCS_Visual Field L, NIH Visual, NIH Facial Palsy, OCS_Hearts OverallAccuracy, OCS_egocentric neglect left, NIH Motor Arm left, NIH Motor Leg left, NIH Sensory, NIH Dysarthria, NIH Inattention]. PC3 loaded on right side motor deficits [NIH

Motor Arm right, NIH Motor Leg right, NIH Facial Palsy, NIH Dysarthria, OCS egocentric neglect Right].

PC1 accounted for 23,5% of the variance, PC2 for 14% of the variance and PC3 for 7.5% of the variance. This structure is represented in **Figure 2A** were the size of each circle is proportional to the percentage of variance explained by each factor across subjects. **Figure 2B** shows the loadings for each score (see **Supplementary Figure 3** for non-rotated PCA loadings results).

The correlation among behavioral scores was also examined through a correlation matrix (**Figure 3**). A 'block' structure along the diagonal indicates correlation among different tests. Consistently with PC1, there was a robust correlation between language, calculation, praxis, verbal and spatial memory tasks, and right allocentric neglect. Left motor deficits correlated with left visual field and left egocentric and allocentric neglect (PC2), while right motor deficits formed a separate cluster (PC3). Interestingly, some tests show positive correlation across two components. For instance, the OCS Heart overall accuracy, a test of general performance, and the NIHSS orientation were common to PC1 and PC2; dysarthria and face palsy, which were not separated in left or right deficit, were common to PC2 and PC3.

In summary, this analysis identified three main sets of correlated behavioral deficits: one cognitive related to language, calculation, praxis, and memory deficits, and two contra-lesional motor-attention components. General performance influenced both the cognitive and left motor-attention component.

Ridge regression Behavior to Anatomy

To study the relationship between structural damage and behavioral impairment we applied a ridge regression model. The analysis was conducted on subjects (n=148) that included both behavioral and neuroimaging data. **Figure 4A** shows the scatter plots of real vs. model predicted scores for each component. Each dot represents a subject, and the size of each dot is scaled by the lesion volume. The model explained different levels of variance for each factor score: PC1: 38%; PC2: 44%; and, PC3: 9%, respectively. As shown by the scatter plots, the model did not provide an accurate prediction of scores for patients with small lesions.

Figure 5 shows the maps of the most predictive anatomical structures (weights of the ridge regression) associated with each PC scores. The anatomical description goes from the dorsal to the ventral slices, and from the anterior to the posterior direction. The orange/yellow color scale indicates damaged voxels associated with low performance, whereas the blue/teal color scale indicates damaged voxels associated with high performance. Here, we focus on anatomical regions

For Review Only

associated with low performance. Low performance on language, memory, calculation, and praxis (PC1) correlated with damage of the left superior and middle frontal gyrus, left inferior parietal and underlying white matter, left occipital dorsal, left inferior frontal gyrus/insula and underlying white matter, left putamen and caudate, left thalamus, and left anterior middle and inferior temporal gyrus. Left motor and attention deficits (PC2) correlated with damage of the right superior, middle, and precentral gyrus, right superior and inferior parietal regions, right corona radiata and internal capsule, right caudate, putamen, and thalamus, and right superior and middle temporal gyrus, right orbitofrontal gyrus. Finally, low scores on right motor and attention deficits (PC3) localized to damage of the left caudate, putamen, and internal capsule, left thalamus, and left lateral occipital cortex.

For Review Only

Validation: WU cohort

To test the external validity of our predictions, we applied the same analysis to the behavioral scores of the WU cohort.(Corbetta *et al.*, 2015) The St. Louis WU cohort includes n=132 first-time stroke patients prospectively enrolled with the same criteria as this study; the behavioral battery takes two and half hours, and includes 44 scores in 7 domains (motor, visual, language, spatial attention, general performance, verbal, and spatial memory). A PCA on the behavioral scores also yielded three components (PC1-3) that explained 49% of the variance, which loaded on similar functional domains (**Supplementary Figure 4**). PC1 (22.5%) loaded on language and verbal/spatial memory; PC2 (15%) on left motor, left visuospatial neglect, general performance, and spatial memory; PC3 (11.4%) on right motor and right spatial neglect.(Corbetta *et al.*, 2015) A ridge regression model explained different levels of variance for each factor (PC1: 13%, PC2: 56% and PC3: 35% respectively), while still providing poor predictions for smaller lesions (**Figure 4B**).

The weights of the ridge regression identified regions of the brain whose damage mostly contributed to the different PC scores. High PC1 scores correlated with damage of several left hemisphere regions: left precentral white matter, left inferior parietal and underlying white matter, left insula and inferior frontal gyrus, left caudate, putamen, and thalamus, left anterior and middle temporal gyrus. This map contained also right hemisphere regions including right precentral white matter, right caudate, putamen, and thalamus, right insula, right anterior temporal gyrus. High PC2 scores localized to the right precentral cortex and underlying corona radiata, right caudate, putamen and internal capsule. A significant region was also in the left middle temporal gyrus. Finally, high PC3 scores correlated with damage to the left precentral gyrus and underlying corona radiata, left internal capsule, putamen, and thalamus, left anterior inferior frontal gyrus (**Figure 6**).

For each PC we evaluated the spatial correlation between the maps obtained with our data and the maps obtained with the WU dataset, after having resampled both maps in the same space of the WU data. For PC1 we obtained a correlation r=0.66 ($p < 10^{-5}$); for PC2 r=0.35 ($p < 10^{-5}$); for PC3 r=0.11 ($p < 10^{-5}$). In general, the topography of damage related to the main axes of behavioral impairment was consistent between both samples of stroke patients.

For Review Only

Discussion

This study investigated whether previously described(Corbetta *et al.*, 2015) groups of correlated deficits describing post-stroke behavioral variability could be validated using a different population and a different neuropsychological battery. Furthermore, we studied whether a simplified neurological and psychological assessment could be used as a sensitive measure of these axes of impairment. Finally, the topography of stroke lesions was analyzed to map the relationship between structural damage and behavioral biomarkers.

The behavior factor analysis showed strong correlation between deficits across domains. Three factors explained ≈ 50% of the variance with Factor 1 loading on functions that are traditionally associated with the left hemisphere: language, verbal memory, calculation, and praxis. However, on Factor 1, we also found visual episodic memory and general performance, functions that are typically associated with the right hemisphere.

In the language domain, subtests evaluated the level of speech production, auditory comprehension, reading capacities, and general performance. All language tasks loaded under PC1 showing correlation that accounted for ≅ 24 % of the whole variability of scores across subjects with no clear separation in the traditional aphasia syndromes, e.g. (Broca, Wernicke). This correlation among language deficits/syndromes is comparable to the St. Louis WU cohort: their PC1 accounted for 22.5% of variance (Corbetta *et al.*, 2015). Interestingly, the Padova PC1 also includes tasks for number processing abilities (Number Writing and Calculation). Number processing is traditionally associated with lesions of the parietal lobe, especially the left parietal (even though an association with right parietal cortex was recently described by Semenza et al.(Montefinese *et al.*, 2017)). PC1 also loaded on praxis, a left fronto-parietal function.(Heilman KM, Watson RT, 2007) Finally, PC1 also loaded on verbal and visual memory, similarly to what we find in the St. Louis battery. Interestingly, the Padova PC1 also includes correlation with right visual neglect and general performance. Overall, then, both Padova and St. Louis PC1 capture correlated deficits in many traditional left hemisphere functions (language, calculation, praxis, verbal memory), but also right hemisphere functions (general performance and visual memory).

PC2 and PC3 capture in both batteries, respectively, left and right motor deficits. The ranking in variance explained is also similar, first left (PC2) then right (PC3) motor deficits. Interestingly, in the motor domain, we do not see the traditional vascular syndromes (e.g. middle vs. anterior cerebral vs. subcortical), but correlated deficits of both upper and lower extremity motor function. This is consistent with prior PCA studies on the NIHSS(Lyden *et al.*, 2004; Zandieh *et al.*, 2012) and Corbetta et al(Corbetta *et al.*, 2015). While traditional neuropsychological and neurophysiological investigations differentiate between sensory versus memory driven movements,

and reaching versus grasping(Kalaska *et al.*, 1997; Rizzolatti *et al.*, 1997; Wise *et al.*, 1997), more recent studies emphasize the correlation among different kinds of ecological movements, and the low dimensionality of movements in terms of kinematic analysis, EMG activation, and even responses in motor cortex. A reaching movement for instance will require coordinated movements of shoulder, arm, elbow, wrist, and fingers that occur together in patterns of neural activation (synergies).(Ingram *et al.*, 2008; Cheung *et al.*, 2009, 2012; Howard *et al.*, 2009)

The OCS does a good job in separating deficits of attention. General performance captured by the overall detection score on the Heart task loads on both PC1 and PC2. PC2 also captures left visual neglect, both egocentric, i.e. centered on the body midline, and allocentric, i.e. centered on the midline of objects, consistently with the syndrome of hemi-spatial neglect(Corbetta and Shulman, 2011). Interestingly, right allocentric neglect loads on PC1 consistent with the observation that this form of neglect is better conceptualized as a left hemisphere object agnosia.(Hillis *et al.*, 2005; Corbetta and Shulman, 2011) Both Padova PC2 and PC3 are highly similar in structure to St. Louis, despite differences in the neuropsychological tests used.

Overall, then our study essentially replicates Corbetta et al. demonstrating that at the population level this low dimensional structure of behavioral impairment is specific to stroke irrespective of population, time of testing (5 days Padova, 2 weeks, 3-12 months St. Louis), and other non-specific factors (i.e. variability in performance, low motivation, anxiety or depression) potentially present at the acute phase.

While the St. Louis battery takes between 1 and ½ and 2 hours being structured in 44 different scores covering multiple domains: motor, language, memory, attention(Corbetta *et al.*, 2015), the neurobehavioral battery in this study was shorter to administer. The Oxford Cognitive Screen (OCS), a validated tool for cognitive assessment in stroke,(Demeyere *et al.*, 2015) can be readily administered in approximately 10 minutes. It has shown high levels of inclusivity, reliability, convergent and divergent validity between subtests and other cognitive tests, such as MOCA, BDAE, Wechsler.(Demeyere *et al.*, 2015) It has been validated in several countries (Demeyere *et al.*, 2015, 2019; Mancuso *et al.*, 2016; Humphreys *et al.*, 2017; Ramos *et al.*, 2018; Huygelier *et al.*, 2019; Robotham *et al.*, 2019) stratified for age, gender and education level. Recent studies have demonstrated high levels of sensitivity in detecting stroke-specific cognitive impairments even in mild stroke.(Mancuso *et al.*, 2018) The NIH stroke scale was designed to be standardized, repeatable, and usable in large multi-center clinical trials.(Brott *et al.*, 1989) Clinical researchers have widely accepted this scale due to high levels of inter-examiner and test-retest score consistency.(Goldstein *et al.*, 1989) In addition, it has been repeatedly validated as an excellent predictor for patient outcome.(Muir *et al.*, 1996) While previous studies on the factor structure of

For Review Only

the sole NIH stroke scale(Lyden *et al.*, 2004; Zandieh *et al.*, 2012) have identified two factors, one for each hemisphere, this study combining the NIHSS and OCS replicates the 3-factor structure identified in Corbetta et al. (Corbetta *et al.*, 2015) This implies that to capture cognitive impairment the NIHSS should be integrated with a more sensitive cognitive screen.

Importantly, we found that the combination of NIHSS and OCS had an excellent level of compliance. We were able to administer all subtests at 5 days to 88% of enrolled patients, against 51% of enrolled patients at 2 weeks on the St. Louis battery. It remains to be seen if the NIHSS/OCS battery will be sensitive to recovery similarly to the St. Louis battery(Ramsey *et al.*, 2017).

It should be underscored, however, that in both datasets a significant amount of behavioral variance (~50%) was not described by our data reduction approach. Where does the rest of the behavioral variance in stroke go? One possibility would be to add more patients hoping that as more lesions sample specific locations in the brain, more specific patterns of behavior will emerge. This is possible, even though we currently feel this is unlikely. In Padova, we carried out a preliminary analysis with n=100 individuals (as compared to n=180 in the final analysis), and we obtained the same three factors explaining about the same amount of variance. In St. Louis, we more than doubled the subjects by running PCA on domain specific components obtained on the maximum number of patients, and the variance accounted increased only by 20%.

So how can we improve our post-stroke behavioral description? It is possible that the percentage will increase as some other important cognitive domains are included (i.e. emotion, decision making, social cognition, theory of mind). In particular, the identified axes of behavioral impairment are similar to the main behavioral axes described in healthy subjects when considering the brain's functional lateralization through fMRI meta-analytic data. Karolis et al., showed that four axes (i.e. symbolic communication, perception/action, emotion and decision-making) could summarize the entire architecture of the brain's lateralization of function.(Karolis *et al.*, 2019) Karolis results could provide a physiological counterpart to the identified post-stroke behavioral biomarkers, but it suggests that at least two additional cognitive domains (emotion and decision-making) shall be added to our short battery to provide a comprehensive behavioral profile of stroke patients.

When considering structural damage, our study demonstrated stroke topography was predominantly subcortical, with a paucity of cortical lesions. Lesions were extremely heterogeneous in volume, including both lacunar and hemispheric strokes. All vascular territories were involved, with the middle cerebral artery predominantly affected in accordance with well-established stroke literature.('Harvard-Oxford cortical and subcortical structural atlases included in FSL', n.d.; Navarro-

Orozco and Sánchez-Manso, 2019) Cortical areas were exclusively affected in only 10% of patients, in agreement with data from other prospective clinical sample studies on acute stroke patients.(Kang *et al.*, 2003; Wessels *et al.*, 2006; Corbetta *et al.*, 2015) A significant portion of the sample (42%) underwent acute reperfusion therapy. Demographic factors and differences in clinical characteristics did not likely bias topography, especially as strong factors associated with subcortical damage (such as hypertension, diabetes type II and hemorrhagic strokes) were actually slightly less represented in our sample in comparison with other consecutive sample studies (see Results).(Bogousslavsky *et al.*, 1988) Once again, these results emphasize how stroke (both in topography and symptoms) should be better conceptualized as a subcortical disease with secondary impact on white matter pathways and cortico-cortical and cortico-subcortical functional interactions.(Corbetta *et al.*, 2018)

Finally, we investigated the relationship between behavioral biomarkers and stroke topography through a multivariate machine learning method. While previous studies have performed factor analysis to identify neural structures of pre-conceived and distinct functional domains (i.e. language, motor, memory, attention),(Butler *et al.*, 2014; Corbetta *et al.*, 2015) the PC scores of our subjects derive from statistical correlation alone and bypass the need of, often overlapping, classifications of deficits to map behavioral markers. Most importantly, this completely data driven approach provides topographical correlates regarding post-stroke multi-domain impairment. High PC1 (language, memory, calculation, praxis) scores mainly correlated with damage of left cortico-subcortical regions; high PC2 scores (left motor and visual attention) with damage of right cortico-subcortical regions; high PC3 scores (right motor) with damage of left subcortical regions. Our structural model was able to explain only low-medium levels of variance for our components (PC1= 38%, PC2= 44% and PC3=9% respectively). While the linear analysis we used was probably too simple to account for data with extremely heterogenous lesion volume, the resulting maps are consistent with those obtained by running the same analysis on the St. Louis data set.(Corbetta *et al.*, 2015) It will be interesting to test whether models that include pathophysiological information (such as white matter disconnection and functional disconnection analysis) will provide better results in different behavioral domains.(Boes *et al.*, 2015; Foulon *et al.*, 2018; Salvalaggio *et al.*, 2020)

In conclusion, this study demonstrated a low dimensional structure of neurological deficits following stroke using a combination of clinically applicable batteries. We identified a few components that showed consistency across different populations and different neurobehavioral batteries. The associated lesion topography of the identified components was also robust.

The identified biomarkers are therefore sensitive measures of behavioral impairment when investigating the epidemiology, genetics, or pathophysiology of stroke. They should also be employed to assess the efficacy at the population level of novel acute or chronic interventions.

For Review Only

Acknowledgments

The principal investigators wish to thank the participants who participated in the study for their time and effort.

Fundings

MC was supported by Progetto Strategico (2016-2020) University of Padova;

NIH NS095741 (2015-2020); FLAG-ERA JTC Brain-Synch Hit (2017-2021); BIAL Foundation Grant (2019-2021); Department of Excellence Italy Ministry of Research (MIUR)(2018-2022); CARIPARO Foundation (2020-2023); Neuro-Connectome, Ministry of Health, Italy (2020-31/12/2023). ALB is supported by the Residency Neurology Program of the University of Padova.

Author Contributions

Study conception by M.C., study design by M.C., C.B., A.M.B., Data acquisition by A.L.B., E.M., S.D., S.F., C.B, A.M.B. Data analysis was done by A.L.B., C.F, A. Z., L.P., M. C.[2]. Manuscript was written by A.L.B., C.F., M.C.

Conflicts of Interest

The authors declare no conflicts of interests.

For Review Only

Supplementary Figure 1. Enrollment Flowchart

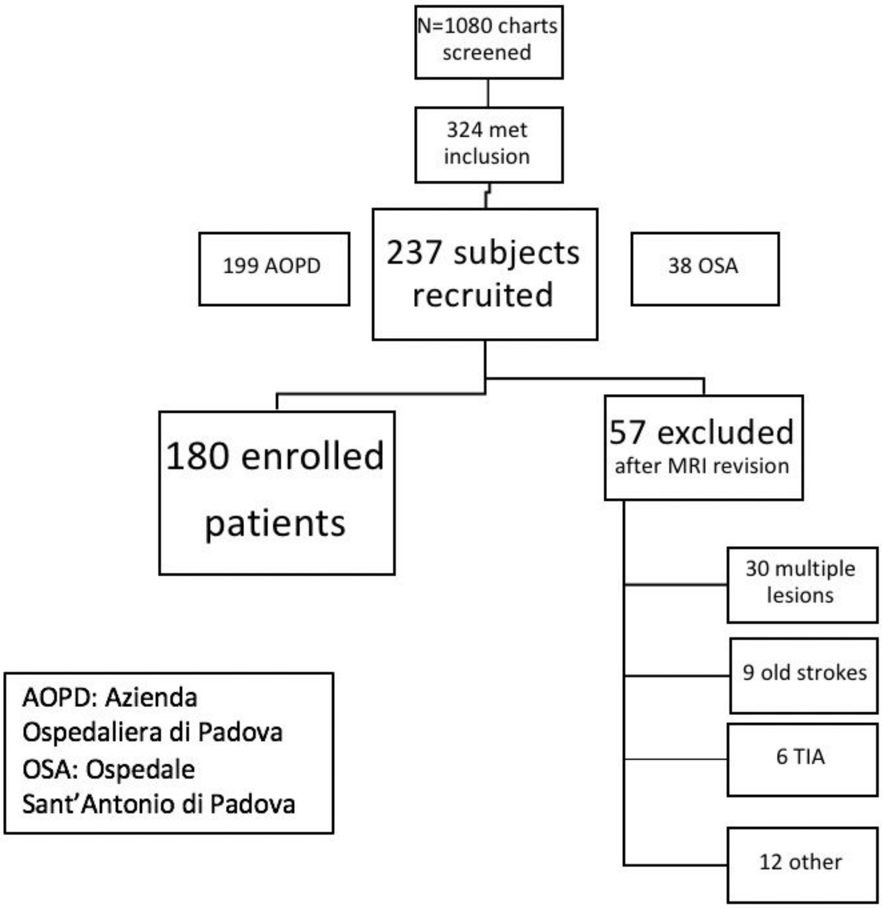

Supplementary Figure 2. Design of the study

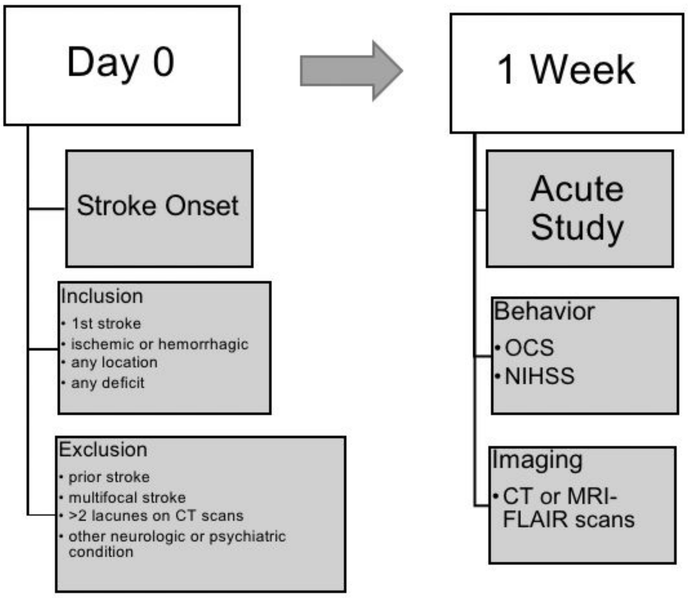

Study Timeline

Only

Supplementary Table 1. OCS and NIHSS individual subtests considered for the behavioral analysis

**OCS**	**Language**	Denomination
		Semantics
		Sentence Reading
	**Number**	Number Writing
		Calculation
	**Memory**	Episodic Memory
		Verbal Memory
		Orientation
	**Praxis**	Imitating Gesture
	**Attention**	Allocentric Neglect
		Egocentric Neglect
		Hearts Overall Accuracy
		Total Executive Function
		Mixed Executive Function
		Visual Field
**NIHSS**		LOC-Vigilance
		LOC-Questions
		LOC-Commands
		Best Gaze
		Visual
		Facial Palsy
		Motor Arm Right
		Motor Leg Right
		Motor Arm Left
		Motor Leg Left
		Limb Ataxia
		Sensory
		Best Language
		Dysarthria
		Inattention

Supplementary Table 2. Demographics & Clinical Characteristics of the Sample

	Study sample
**Age**	
18-30	1,5%
31-50	10,5%
51-70	29,5%
71 or more	58,5%
**Gender**	
Female	47%
Male	53%
**Education**	
Middle School or less	55%
High school	26%
Incomplete college	8%
College or post-graduate	11%
**Risk Factors**	
Hypertension	64%
Diabetes mellitus	24%
Coronary artery Disease	12%
Atrial Fibrillation	15%
Smoking	23%
**Stroke Severity (NIHSS)**	
Mild 0-6	58%
Moderate 7-15	28%
Severe 16-42	14%
**Stroke Symptoms**	
Neglect	20%
Aphasia	35%
Motor Impairment	90%
**Lesion Side**	
Right Cerebral	40%
Left Cerebral	44%
Cerebellum/Midbrain	7.5%
Negative scan	9%
**Stroke Subtype**	
Ischemic	89%
[illegible]	[illegible]

Supplementary Table 3. Acute Reperfusion Therapy details. rtPA= recombinant tissue Plasminogen Activator; Tot. OCS= number of impaired OCS subtests (according to normative cut-offs).

Stroke Sample (n=180)	N. of patients	Mean NIHSS score Admiss.	Mean NIHSS score at NPSY	Mean Tot. OCS score
**No acute treatment**	104 (58%)	4.3	2.7	4.2
**Acute treatment**	76 (42%)	10.8	4.1	5.5
• **rtPA**	37 (20.4%)	7.7	2.8	4.2
• **Thrombectomy**	18 (10%)	12.8	6.4	7
• **rTPA+Thrombectomy**	21 (11.6%)	14.6	4.4	6.5

For Review Only

Supplementary Table 4. Neuroimaging lesion details

Stroke ID	Stroke Etiology	Lesion Description	Lesion Volume (in voxels of 2x2x2 mm)	Lesion Volume (in ml)
**1**	1	right basal ganglia	1060	8,48
**2**	2	left occipital	5232	41,856
**4**	1	right occipital	2911	23,288
**5**	2	left temporal	9444	75,552
**6**	1	right frontal	1625	13
**7**	1	left basal ganglia	6001	48,008
**8**	1	left superior temporal	6585	52,68
**10**	1	right posterior temporal	228	1,824
**11**	1	right corona radiata	300	2,4
**13**	1	right frontal	46	0,368
**14**	1	left frontal	2345	18,76
**15**	1	right basal ganglia	2256	18,048
**19**	2	left basal ganglia	98	0,784
**21**	1	left pons	168	1,344
**22**	1	right basal ganglia	343	2,744
**23**	1	right basal ganglia	401	3,208
**24**	1	left frontal	2015	16,12
**25**	1	right frontal	171	1,368
**27**	1	left frontal	5478	43,824
**28**	2	left temporo-parietal	1865	14,92
**29**	1	right basal ganglia	152	1,216
**30**	1	left frontal	22	0,176
**31**	1	left fronto-temporo-parietal	11428	91,424
**32**	1	left cerebellar	142	1,136
**33**	1	right parietal	11725	93,8
**34**	1	right superior pons	339	2,712
**35**	1	left parietal	22	0,176
**36**	1	right internal capsule	95	0,76

For Review Only

**37**	1	left basal ganglia	166	1,328
**38**	2	right basal ganglia	5348	42,784
**39**	1	left temporal and basal ganglia	11772	94,176
**41**	1	left basal ganglia	1019	8,152
**42**	1	left basal ganglia	22	0,176
**43**	1	right parietal	167	1,336
**44**	2	right basal ganglia	1188	9,504
**45**	1	left fronto-parietal	1698	13,584
**46**	2	left basal ganglia	2243	17,944
**48**	1	temporal	5529	44,232
**49**	1	left frontal	122	0,976
**50**	1	left internal capsule	39	0,312
**51**	1	left basal ganglia and temporal	3914	31,312
**52**	1	left frontotemporoparietal	7797	62,376
**53**	1	left frontal	4121	32,968
**54**	1	right parietal	3393	27,144
**55**	2	right basal ganglia	4049	32,392
**57**	1	left fronto parietal	3368	26,944
**58**	1	left basal ganglia	230	1,84
**59**	1	right frontal	22	0,176
**60**	1	right frontoparietal	1422	11,376
**61**	2	right occipital	6349	50,792
**62**	1	left basal ganglia	2232	17,856
**63**	1	left corona radiata	165	1,32
**64**	1	left cortico-subortical temporo-parietal	2980	23,84
**67**	1	right hippocampus	29	0,232
**68**	1	left frontal	452	3,616
**69**	1	left frontal	1188	9,504
**71**	1	left cerebellar	6149	49,192
**72**	1	right hippocampus	402	3,216
**74**	1	right frontal	121	0,968
**75**	1	left frontal	687	5,496

For Review Only

**76**	1	right occipital	2877	23,016
**78**	1	right pons	33	0,264
**79**	1	left frontal	27	0,216
**81**	1	right subcortical	14	0,112
**85**	1	right frontal	546	4,368
**87**	1	right frontal	158	1,264
**90**	1	right cerebellar	45	0,36
**91**	1	left parieto temporal	1447	11,576
**92**	1	right thalamus	34	0,272
**93**	1	right subcortical	2479	19,832
**94**	2	right putamen	17723	141,784
**95**	1	right cerebellar	336	2,688
**96**	1	left temporal	5200	41,6
**98**	1	right internal capsule	220	1,76
**99**	1	left subcortical	774	6,192
**101**	1	left occipital	2920	23,36
**102**	1	right corona radiata	130	1,04
**104**	1	right frontal	129	1,032
**106**	1	right parietal	37344	298,752
**107**	1	left temporo-hyppocampal	3760	30,08
**108**	1	right frontal	18	0,144
**109**	2	right fronto-parietal	29679	237,432
**110**	1	left frontal	44	0,352
**115**	1	left frontal	529	4,232
**117**	1	left frontal	11424	91,392
**118**	1	left frontal	2528	20,224
**120**	1	right parietal	10316	82,528
**121**	1	left tempor-parieto-occipital	1734	13,872
**122**	1	left frontal	6851	54,808
**124**	1	left fronto-parietal	25	0,2
**125**	1	right subcortical	57	0,456
**126**	1	right thalamus	169	1,352
**127**	1	left temporoparietal	757	6,056

For Review Only

**129**	1	right occipital	3125	25
**130**	1	left basal ganglia	957	7,656
**132**	1	left subcortical	1695	13,56
**133**	1	right frontal	623	4,984
**135**	2	left basal ganglia	19443	155,544
**136**	1	left frontal	852	6,816
**137**	1	left fronto-temporal	1911	15,288
**138**	1	right parietal	1853	14,824
**139**	1	right fronto-temporal	20114	160,912
**140**	1	left frontal	228	1,824
**141**	1	left corona radiata	442	3,536
**142**	1	left pons	334	2,672
**143**	1	right temporal	1234	9,872
**144**	1	right thalamus	270	2,16
**145**	1	rght temporal	19782	158,256
**146**	1	left thalamus	28	0,224
**148**	1	left thalamus	19	0,152
**149**	1	left thalamus	979	7,832
**150**	2	right basal ganglia	3160	25,28
**151**	1	right frontal	729	5,832
**152**	1	left front-parietal	186	1,488
**154**	1	right basal ganglia	752	6,016
**156**	1	left cerebellar	168	1,344
**157**	1	right basal ganglia	6321	50,568
**159**	1	right subcortical and basal ganglia	5848	46,784
**161**	1	right temporo-occipital	1329	10.632
**163**	1	Left cerebellar hemisphere	6981	55.848
**165**	1	Left caudate and internal capsule	499	3.992
**166**	1	Left caudate and anterior internal capsule	316	2.528
**167**	1	Left precentral gyrus	3011	24.088
**168**	1	Left temporo-parietal	3253	26.016

For Review Only

**169**	1	Left capsular	737	5.896
**171**	1	Left frontal cortico-subcortical	647	5.176
**172**	1	Left capsular	346	2.768
**173**	1	Left fronto-temporal	21699	173.592
**175**	2	Right occipital	7061	56.488
**178**	2	Left nucleo capsular	2440	19.52
**181**	1	Left parietal and internal capsule	730	5.84
**182**	1	Right occipito-mesial	3333	26.664
**184**	2	Right thalamus	331	2.648
**185**	1	Left fronto-parietal	652	5.216
**186**	1	Right corona radiata	1437	11.496
**187**	2	Right parieto-occipital	4471	35.768
**188**	1	Left tempo-parietal	2486	19.888
**190**	1	Right parietal	1122	8.976
**194**	1	Left fronto-parietal	758	6.064
**195**	1	Left prefrontal gyrus	23	0.184
**196**	1	left temporo-parietal	20544	164.352
**198**	1	Left fronto-parietal	62	0.496
**199**	1	Left frontal	534	4.272
**200**	1	Left corona radiata	113	0.904
**202**	1	Left frontal	7650	61.2
**203**	1	Left fronto-opercular	1107	8.856
**205**	1	Left temporal	3835	30.68
**208**	1	Left caudate and putamen	2747	21,976
**212**	1	Left frontal and callosal body	279	2.232
**215**	2	Left cerebellar	3618	28.944
**216**	1	Right occipital	2538	20.304
**221**	1	Left fronto-parietal	10325	82.6
**223**	1	Left frontal	4188	33.504
**224**	1	Left temporo-parietal	3097	24.776
**225**	1	Left insular	3035	24.28
**226**	1	Left posterior insular	633	5.064
**227**	1	Right nucleo-capsular	8627	69.016

For Review Only

**233**	1	Right fronto-parietal	17483	139.864
**235**	1	Medial thalamus	2731	21.848
**236**	1	Right precentral gyrus	799	6.398
**237**	1	Right cerebellar	742	5.936
**Average**			3357	26.0

For Review Only

Supplementary Table 5. Harvard-Oxford Cortico-Subcortical Parcellation: We computed the mean, standard deviation and maximum values (expressed as percentage) of parcels affected by the lesions of our sample from the Harvard-Oxford atlas.

ROI (Subcortical Mask)	Mean % of parcel affected	Std	Max
**Left Cerebral White Matter**	2.00	2.09	13.69
**Left Cerebral Cortex**	0.93	1.59	13.10
**Left Lateral Ventricle**	2.27	1.30	7.14
**Left Thalamus**	1.78	1.31	5.95
**Left Caudate**	5.50	1.88	9.52
**Left Putamen**	6.95	2.63	13.10
**Left Pallidum**	5.11	1.42	8.93
**Brain-Stem**	0.23	0.41	2.38
**Left Hippocampus**	1.46	1.11	4.17
**Left Amygdala**	2.76	1.40	5.95
**Left Accumbens**	1.36	1.07	3.57
**Right Cerebral White Matter**	2.73	1.97	11.90
**Right Cerebral Cortex**	0.78	1.31	10.12
**Right Lateral Ventricle**	2.84	0.99	6.55
**Right Thalamus**	2.65	1.63	7.74
**Right Caudate**	4.14	1.22	7.14
**Right Putamen**	6.24	1.91	10.71
**Right Pallidum**	5.31	1.38	7.74
**Right Hippocampus**	1.33	1.20	4.76
**Right Amygdala**	1.31	1.34	6.55
**Right Accumbens**	1.55	0.86	3.57

ROI (Cortical Mask)	Mean % of parcel affected	Std	Max
**Frontal Pole**	0.18	0.44	5.36
**Insular Cortex**	7.00	1.97	13.10
**Superior Fronmtal Gyrus**	0.31	0.44	2.98
**Middle Frontal Gyrus**	1.02	1.11	6.55
**Inferior Frontal Gyrus pars triangularis**	1.56	1.21	4.76

For Review Only

**Inferior Frontal Gyrus. pars opercularis**	2.62	1.72	7.14
**Precentral Gyrus**	1.15	1.39	7.74
**Temporal Pole**	1.12	1.26	8.33
**Superior Temporal Gyrus. anterior division**	2.66	2.04	8.33
**Superior Temporal Gyrus. posterior division**	3.07	2.12	8.33
**Middle Temporal Gyrus. anterior division**	1.12	1.14	5.95
**Middle Temporal Gyrus. posterior division**	1.83	1.78	7.74
**Middle Temporal Gyrus. temporooccipital part**	2.37	1.74	7.74
**Inferior Temporal Gyrus. anterior division**	0.66	0.81	3.57
**Inferior Temporal Gyrus. posterior division**	0.48	0.68	4.17
**Inferior Temporal Gyrus. temporooccipital part**	0.52	0.83	4.17
**Postcentral Gyrus**	0.70	1.02	6.55
**Superior Parietal Lobule**	0.91	0.82	5.36
**Supramarginal Gyrus. anterior division**	1.78	1.42	5.36
**Supramarginal Gyrus. posterior division**	2.43	1.64	7.74
**Angular Gyrus**	2.30	1.59	7.14
**Lateral Occipital Cortex. superior division**	1.08	1.18	5.95
**Lateral Occipital Cortex. inferior division**	1.17	1.44	6.55
**Intracalcarine Cortex**	2.25	1.68	6.55
**Frontal Medial Cortex**	0.10	0.31	2.38
**Juxtapositional Lobule cortex (formerly Supplementary motor cortex)**	0.20	0.33	2.38
**Subcallosal Cortex**	0.44	0.65	4.17
**Paracingulate Gyrus**	0.14	0.28	2.38
**Cingulate Gyrus. anterior division**	0.46	0.62	4.76
**Cingulate Gyrus. posterior division**	0.49	0.75	4.76
**Precuneous Cortex**	0.65	0.94	5.36
**Cuneal Cortex**	1.30	1.03	5.36
**Frontal Orbital Cortex**	1.54	1.51	7.14
**Parahippocampal Gyrus. anterior division**	0.82	1.14	6.55
**Parahippocampal Gyrus. posterior division**	0.86	1.07	4.76

For Review Only

**Lingual Gyrus**	1.12	1.36	6.55
**Temporal Fusiform Cortex. anterior division**	0.52	0.81	3.57
**Temporal Fusiform Cortex. posterior division**	0.74	0.91	4.76
**Temporal Occipital Fusiform Cortex**	0.68	0.86	4.76
**Occipital Fusiform Gyrus**	0.95	1.17	7.14
**Frontal Operculum Cortex**	4.08	1.29	7.14
**Central Opercular Cortex**	4.35	1.48	9.52
**Parietal Operculum Cortex**	3.68	1.38	8.33
**Planum Polare**	4.86	1.56	11.31
**Heschl's Gyrus (inculdes H1 and H2)**	4.91	1.52	8.93
**Planum Temporale**	4.09	1.30	9.52
**Supracalcarine Cortex**	1.69	1.51	5.36
**Occipital Pole**	0.67	0.87	5.95

For Review Only

Supplementary Figure 3. Non-rotated Principal Component Analysis results and ridge regression maps on the Padova University stroke sample (total variance explained=45%; ridge regression maps p values: PC1>10*-5, PC2< 10*-5, PC3 0.0044).

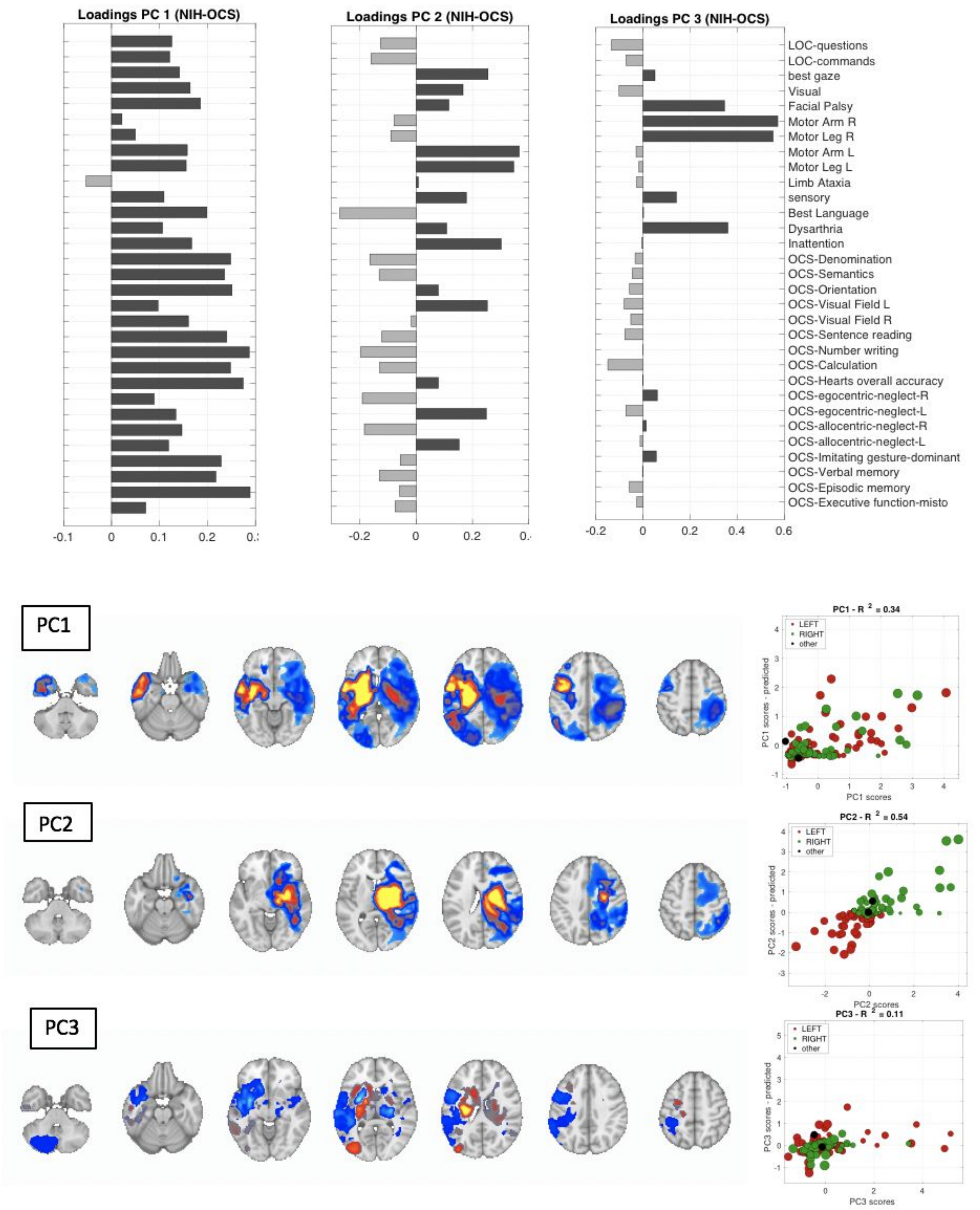

Supplementary Figure 4. Washington University cohort PCA scores**:** after z-scoring of behavioral scores, a principal component analysis (PCA) reduced the number of variables to describe the across subject variability of behavioral deficits (See Corbetta et al. Neuron 2015 for full list of tests).

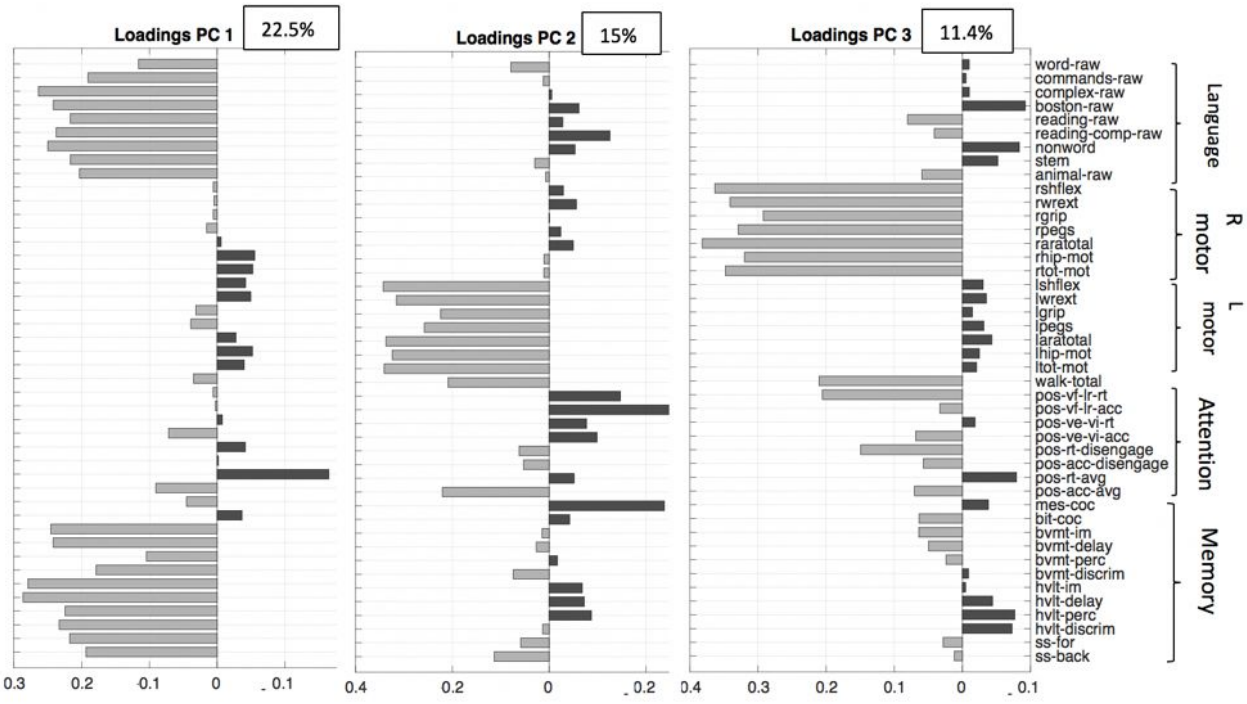

FIGURES

Figure 1. Lesion Topography. Overlay of damage in atlas space (n=164). The color bar represents the percentage of lesions affecting each voxel (anatomical view).

Figure 2. Behavior Factor Analysis. A) The percentage of variance explained by each Principal Component is proportional to the diameter of each circle. The position of each circle on the brain atlas represents the Principal Component's lateralization. Finally, circles are labeled with the main subtests underlying each PC, the font size reflects the relative role of each loading. B) Each table graphically shows single loadings of OCS and NIHSS subtests (on the right) for each PC. Black bars represent positive correlation, while grey bars represent negative correlation.

Figure 3. Correlation matrix of behavioral subtests. The color bar represents Pearson r-values. Each square corresponds to the variables identified through the PCA analysis (i.e. PC1, PC2, PC3).

Figure 4. Ridge regression scatter plots. Factor scores for right (in green) and left (in red) lesions (in black for midline lesions). The diameter of each colored circle is proportional to the lesions volume. Each lesion is associated to three Principal Component values (X axis) and the corresponding predictions of our model (Y axis). **4A)** are calculated on the University of Padua data, while **4B)** are calculated on the Washington University data.

Figure 5. Ridge regression maps from the University of Padova sample. Warm colors represent positive correlation between anatomical voxels and high PC values (i.e. high level of impairment in the corresponding domains). Cold colors represent negative correlation between anatomical voxels and high PC values. Anatomical overlay maps are shown for PC1, PC2 and PC3 scores, respectively (after Gaussian smoothing (variance = 1) and scaling within $[-1, +1]$; weights lower than 0.05 in absolute values are not shown).

Figure 6. Ridge regression maps from the Washington University sample. Warm colors represent positive correlation between anatomical voxels and high PC values (i.e. high level of impairment in the corresponding domains). Cold colors represent negative correlation between anatomical voxels and high PC values. Anatomical overlay maps are shown for PC1, PC2 and PC3 scores, respectively (after Gaussian smoothing (variance = 1) and scaling within $[-1, +1]$; weights lower than 0.05 in absolute values are not shown).

For Review Only

REFERENCES

Avants BB, Tustison NJ, Song G, Cook PA, Klein A, Gee JC. A reproducible evaluation of ANTs similarity metric performance in brain image registration. Neuroimage 2011; 54: 2033–44.

Baldassarre A, Ramsey LE, Siegel JS, Shulman GL, Corbetta M. Brain connectivity and neurological disorders after stroke. Curr Opin Neurol 2016; 29: 706–13.

Boes AD, Prasad S, Liu H, Liu Q, Pascual-Leone A, Caviness VS, et al. Network localization of neurological symptoms from focal brain lesions. Brain 2015; 138: 3061–75.

Bogousslavsky J, Van Melle G, Regli F. The Lausanne Stroke Registry: analysis of 1,000 consecutive patients with first stroke. Stroke 1988; 19: 1083–92.

Broca P. Remarks on the seat of the faculty of articulated language, following an observation of aphemia (loss of speech). Bull Soc Anat 1861; 6: 330–57.

Brott T, Adams HP, Olinger CP, Marler JR, Barsan WG, Biller J, et al. Measurements of acute cerebral infarction: a clinical examination scale. Stroke 1989; 20: 864–70.

Butler RA, Lambon Ralph MA, Woollams AM. Capturing multidimensionality in stroke aphasia: mapping principal behavioural components to neural structures. Brain 2014; 137: 3248–66.

Carter AR, Astafiev S V, Lang CE, Connor LT, Rengachary J, Strube MJ, et al. Resting interhemispheric functional magnetic resonance imaging connectivity predicts performance after stroke. Ann Neurol 2010; 67: 365–75.

Cheung VCK, Piron L, Agostini M, Silvoni S, Turolla A, Bizzi E. Stability of muscle synergies for voluntary actions after cortical stroke in humans. Proc Natl Acad Sci 2009; 106: 19563–8.

Cheung VCK, Turolla A, Agostini M, Silvoni S, Bennis C, Kasi P, et al. Muscle synergy patterns as physiological markers of motor cortical damage. Proc Natl Acad Sci 2012; 109: 14652–6.

CM F. Clinical syndromes in cerebral artery occlusion. In: Fields WS Pathogenesis and treatment of cerebrovascular disease. Charles C. Thomas. 1961

Corbetta M, Ramsey L, Callejas A, Baldassarre A, Hacker CD, Siegel JS, et al. Common Behavioral Clusters and Subcortical Anatomy in Stroke. Neuron 2015; 85: 927–41.

Corbetta M, Shulman GL. Spatial neglect and attention networks. Annu Rev Neurosci 2011; 34: 569–99.

Corbetta M, Siegel JS, Shulman GL. On the low dimensionality of behavioral deficits and alterations of brain network connectivity after focal injury HHS Public Access. Cortex 2018; 107: 229–37.

Demeyere N, Riddoch MJ, Slavkova ED, Bickerton W-L, Humphreys GW. The Oxford Cognitive Screen (OCS): Validation of a stroke-specific short cognitive screening tool. Psychol Assess 2015;

For Review Only

27: 883–94.

Demeyere N, Riddoch MJ, Slavkova ED, Jones K, Reckless I, Mathieson P, et al. Domain-specific versus generalized cognitive screening in acute stroke. J Neurol 2016; 263: 306–15.

Demeyere N, Sun S, Milosevich E, Vancleef K. Post-stroke cognition with the Oxford Cognitive Screen vs Montreal Cognitive Assessment: a multi-site randomized controlled study (OCS-CARE). AMRC Open Res 2019; 1: 12.

Foulon C, Cerliani L, Kinkingnéhun S, Levy R, Rosso C, Urbanski M, et al. Advanced lesion symptom mapping analyses and implementation as BCBtoolkit. Gigascience 2018; 7: 1–17.

Goldstein LB, Bertels C, Davis JN. Interrater Reliability of the NIH Stroke Scale. Arch Neurol 1989; 46: 660–2.

He BJ, Snyder AZ, Vincent JL, Epstein A, Shulman GL, Corbetta M. Breakdown of Functional Connectivity in Frontoparietal Networks Underlies Behavioral Deficits in Spatial Neglect. Neuron 2007; 53: 905–18.

Heilman KM, Watson RT G-RL. Praxis. 2007

Hillis A, Newhart M, Heidler J, Marsh EB, Barker P, Degaonkar M. The neglected role of the right hemisphere in spatial representation of words for reading. Aphasiology 2005; 19: 225–38.

Howard IS, Ingram JN, Körding KP, Wolpert DM. Statistics of natural movements are reflected in motor errors. J Neurophysiol 2009; 102: 1902–10.

Humphreys GW, Duta MD, Montana L, Demeyere N, McCrory C, Rohr J, et al. Cognitive Function in Low-Income and Low-Literacy Settings: Validation of the Tablet-Based Oxford Cognitive Screen in the Health and Aging in Africa: A Longitudinal Study of an INDEPTH Community in South Africa (HAALSI). Journals Gerontol Ser B Psychol Sci Soc Sci 2017; 72: 38–50.

Huygelier H, Schraepen B, Demeyere N, Gillebert CR. The Dutch version of the Oxford Cognitive Screen (OCS-NL): normative data and their association with age and socio-economic status. Aging, Neuropsychol Cogn 2019

Ingram JN, Körding KP, Howard IS, Wolpert DM. The statistics of natural hand movements. Exp brain Res 2008; 188: 223–36.

Jackson. Suggestions on studying diseases of the central nervous system on Professor Owen's Vertebral Theory. 1863

Jenkinson M, Beckmann CF, Behrens TEJ, Woolrich MW, Smith SM. FSL. Neuroimage 2012; 62: 782–90.

Kalaska JF, Scott SH, Cisek P, Sergio LE. Cortical control of reaching movements. Curr Opin Neurobiol 1997; 7: 849–59.

Kang D-W, Chalela JA, Ezzeddine MA, Warach S. Association of Ischemic Lesion Patterns on

For Review Only

Early Diffusion-Weighted Imaging With TOAST Stroke Subtypes. Arch Neurol 2003; 60: 1730.
Karolis VR, Corbetta M, Thiebaut de Schotten M. The architecture of functional lateralisation and its relationship to callosal connectivity in the human brain. Nat Commun 2019; 10: 1417.
Lyden P, Claesson L, Havstad S, Ashwood T, Lu M. Factor Analysis of the National Institutes of Health Stroke Scale in Patients With Large Strokes. Arch Neurol 2004; 61: 1677.
Mah Y-H, Husain M, Rees G, Nachev P. Human brain lesion-deficit inference remapped. Brain 2014; 137: 2522–31.
Mancuso M, Demeyere N, Abbruzzese L, Damora A, Varalta V, Pirrotta F, et al. Using the Oxford Cognitive Screen to Detect Cognitive Impairment in Stroke Patients: A Comparison with the Mini-Mental State Examination. Front Neurol 2018; 9: 101.
Mancuso M, Varalta V, Sardella L, Capitani D, Zoccolotti P, Antonucci G, et al. Italian normative data for a stroke specific cognitive screening tool: the Oxford Cognitive Screen (OCS). Neurol Sci 2016; 37: 1713–21.
Montefinese M, Turco C, Piccione F, Semenza C. Causal role of the posterior parietal cortex for two-digit mental subtraction and addition: A repetitive TMS study. Neuroimage 2017; 155: 72–81.
Muir KW, Weir CJ, Murray GD, Povey C, Lees KR. Comparison of neurological scales and scoring systems for acute stroke prognosis. Stroke 1996; 27: 1817–20.
Navarro-Orozco D, Sánchez-Manso JC. Neuroanatomy, Middle Cerebral Artery [Internet]. 2019[cited 2019 Sep 13] Available from: https://www.ncbi.nlm.nih.gov/books/NBK526002/
Phan TG, Chen J, Donnan G, Srikanth V, Wood A RD. Development of a new tool to correlate stroke outcome with infarct topography: a proof-of-concept study. Neuroimage 2010: 127–33.
Problems N, Hoerl AE, Kennard RW. American Society for Quality Ridge Regression: Biased Estimation for [Internet]. 1970[cited 2020 Jul 31] Available from: https://www.math.arizona.edu/~hzhang/math574m/Read/RidgeRegressionBiasedEstimationForNonorthogonalProblems.pdf
Ramos CCF, Amado DK, Borges CR, Bergamaschi E, Nitrini R, Brucki SMD. Oxford cognitive screen - Brazilian portuguese version (OCS-Br): A pilot study. Dement e Neuropsychol 2018; 12: 427–31.
Ramsey LE, Siegel JS, Lang CE, Strube M, Shulman GL, Corbetta M. Behavioural clusters and predictors of performance during recovery from stroke. Nat Hum Behav 2017; 1: 0038.
Rizzolatti G, Fogassi L, Gallese V. Parietal cortex: from sight to action. Curr Opin Neurobiol 1997; 7: 562–7.
Robotham RJ, Riis JO, Demeyere N. A Danish version of the Oxford cognitive screen: a stroke-specific screening test as an alternative to the MoCA. Aging, Neuropsychol Cogn 2019: 1–14.

For Review Only

Rorden C, Bonilha L, Fridriksson J, Bender B, Karnath H-O. Age-specific CT and MRI templates for spatial normalization. Neuroimage 2012; 61: 957–65.

Salvalaggio A, De Filippo De Grazia M, Zorzi M, Thiebaut de Schotten M, Corbetta M. Post-stroke deficit prediction from lesion and indirect structural and functional disconnection. Brain 2020; 143: 2173–88.

Siegel JS, Ramsey LE, Snyder AZ, Metcalf N V, Chacko R V, Weinberger K, et al. Disruptions of network connectivity predict impairment in multiple behavioral domains after stroke. Proc Natl Acad Sci U S A 2016; 113: E4367-76.

W.T. Longstreth, Teri A. Manolio, Alice Arnold, Gregory L. Burke, Nick Bryan, Charles A. Jungreis, Paul L. Enright, Daniel O'Leary LF. Clinical Correlates of White Matter Findings on Cranial Magnetic Resonance Imaging of 3301 Elderly People The Cardiovascular Health Study. Stroke 1996

Wahlund LO, Barkhof ; F, Fazekas ; F, Bronge ; L, Augustin ; M, Sjögren ; M, et al. A New Rating Scale for Age-Related White Matter Changes Applicable to MRI and CT [Internet]. 2001[cited 2019 Mar 31] Available from: http://www.strokeaha.org

Wessels T, Wessels C, Ellsiepen A, Reuter I, Trittmacher S, Stolz E, et al. Contribution of diffusion-weighted imaging in determination of stroke etiology. AJNR Am J Neuroradiol 2006; 27: 35–9.

Wise SP, Boussaoud D, Johnson PB, Caminiti R. PREMOTOR AND PARIETAL CORTEX: Corticocortical Connectivity and Combinatorial Computations. Annu Rev Neurosci 1997; 20: 25–42.

Yushkevich PA, Piven J, Hazlett HC, Smith RG, Ho S, Gee JC, et al. User-guided 3D active contour segmentation of anatomical structures: Significantly improved efficiency and reliability. Neuroimage 2006; 31: 1116–28.

Zandieh A, Kahaki ZZ, Sadeghian H, Pourashraf M, Parviz S, Ghaffarpour M, et al. The Underlying Factor Structure of National Institutes of Health Stroke Scale: An Exploratory Factor Analysis. Int J Neurosci 2012; 122: 140–4.

Harvard-Oxford cortical and subcortical structural atlases included in FSL.

For Review Only

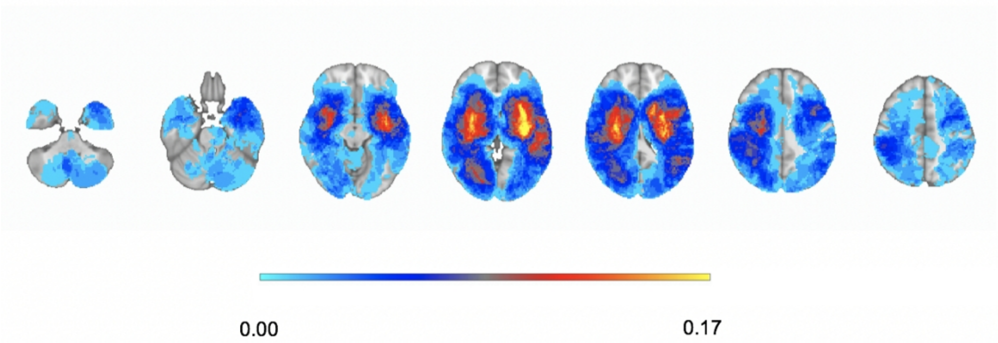

Figure 1 Lesion Topography. Overlay of damage in atlas space (n=164). The color bar represents the percentage of lesions affecting each voxel (anatomical view).

230x81mm (144 x 144 DPI)

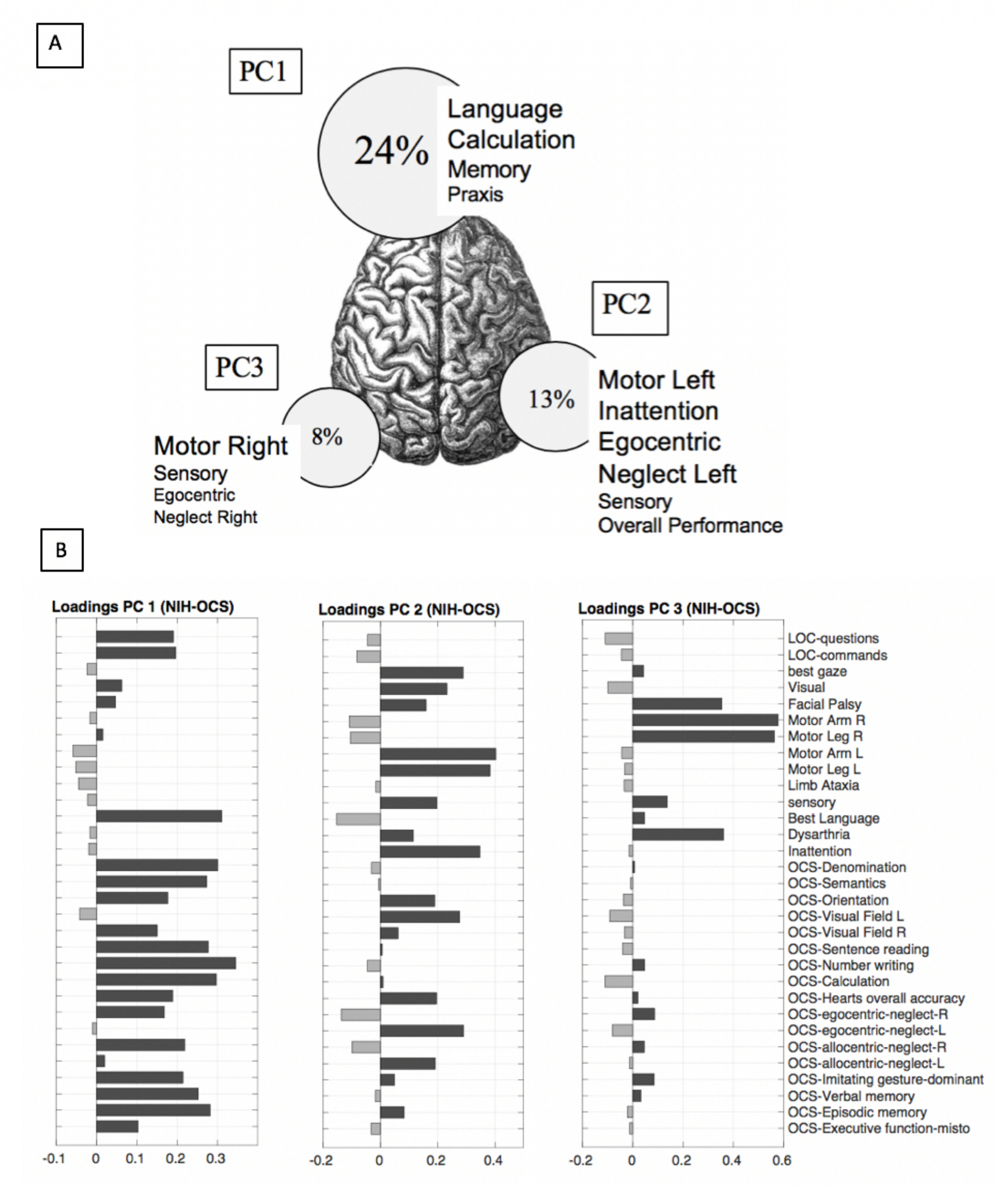

Figure 2- Behavior Factor Analysis. A) The percentage of variance explained by each Principal Component is proportional to the diameter of each circle. The position of each circle on the brain atlas represents the Principal Component's lateralization. Finally, circles are labeled with the main subtests underlying each PC, the font size reflects the relative role of each loading. B) Each table graphically shows single loadings of OCS and NIHSS subtests (on the right) for each PC. Black bars represent positive correlation, while grey bars represent negative correlation.

170x202mm (144 x 144 DPI)

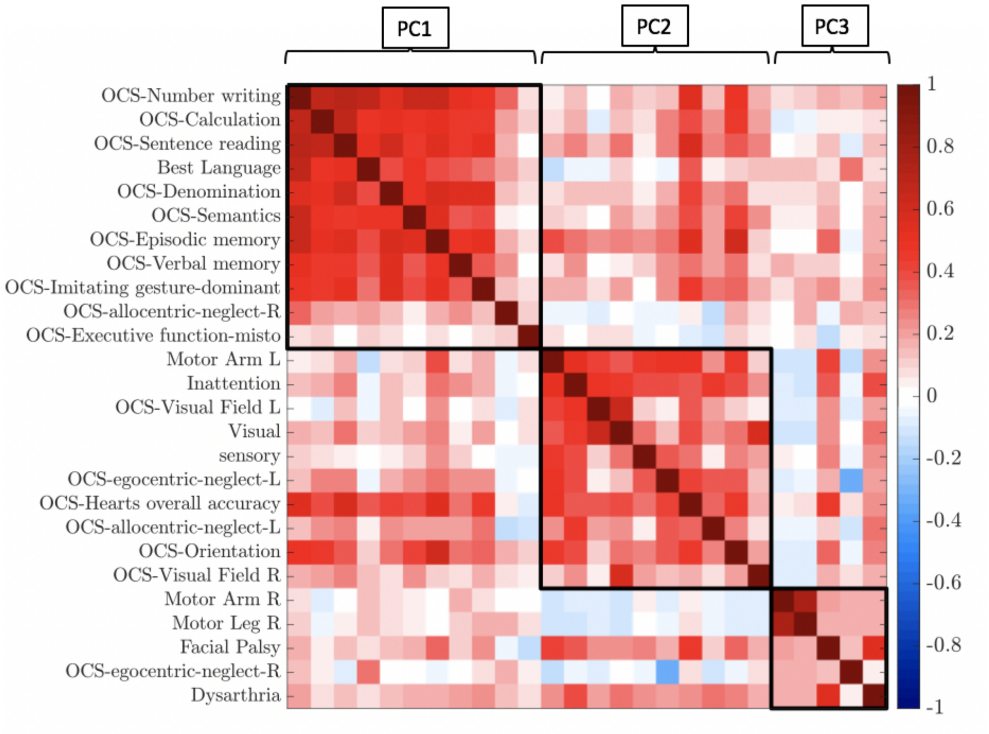

Figure 3- Correlation matrix of behavioral subtests. The color bar represents Pearson r-values. Each square corresponds to the variables identified through the PCA analysis (i.e. PC1, PC2, PC3).

166x122mm (144 x 144 DPI)

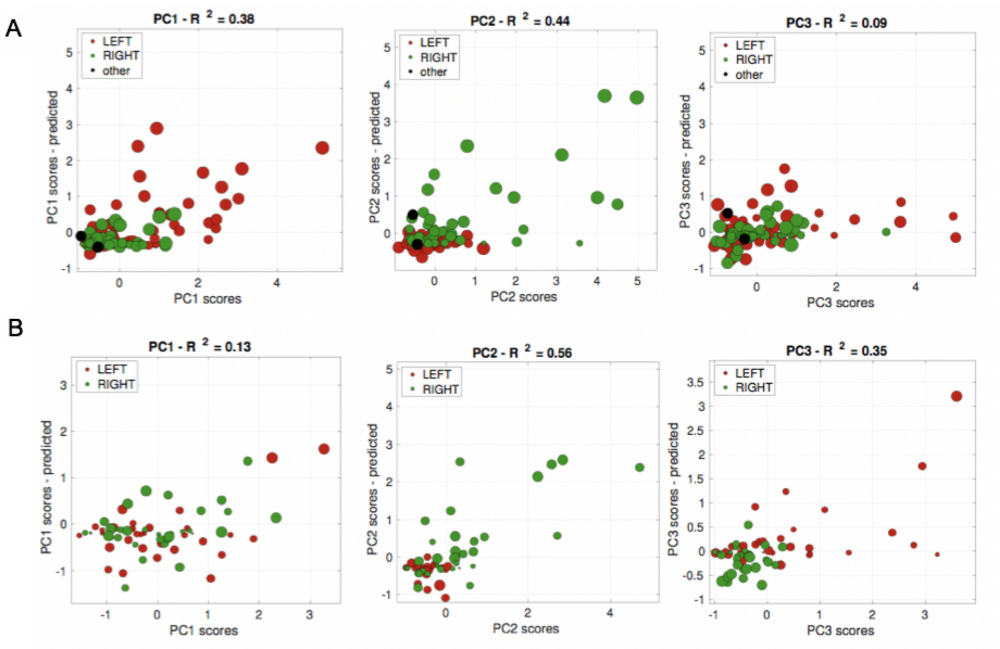

Figure 4- Ridge regression scatter plots. Factor scores for right (in green) and left (in red) lesions (in black for midline lesions). The diameter of each colored circle is proportional to the lesions volume. Each lesion is associated to three Principal Component values (X axis) and the corresponding predictions of our model (Y axis). 4A) are calculated on the University of Padua data, while 4B) are calculated on the Washington University data.

162x105mm (144 x 144 DPI)

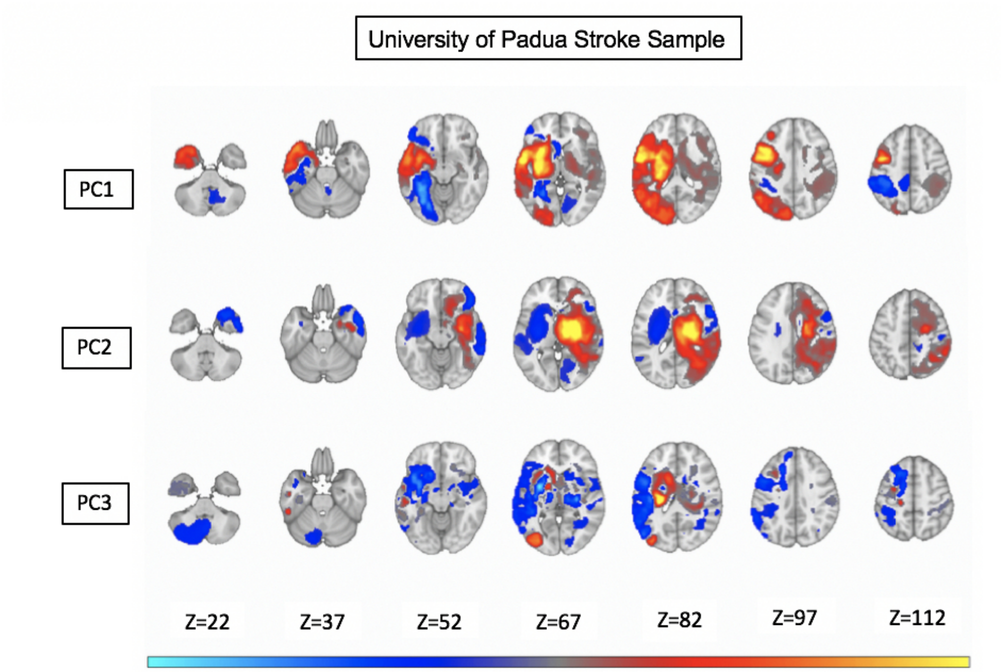

Figure 5-Ridge regression maps from the University of Padova sample. Warm colors represent positive correlation between anatomical voxels and high PC values (i.e. high level of impairment in the corresponding domains). Cold colors represent negative correlation between anatomical voxels and high PC values. Anatomical overlay maps are shown for PC1, PC2 and PC3 scores, respectively (after Gaussian smoothing (variance = 1) and scaling within [-1,+1]; weights lower than 0.05 in absolute values are not shown).

165x115mm (144 x 144 DPI)

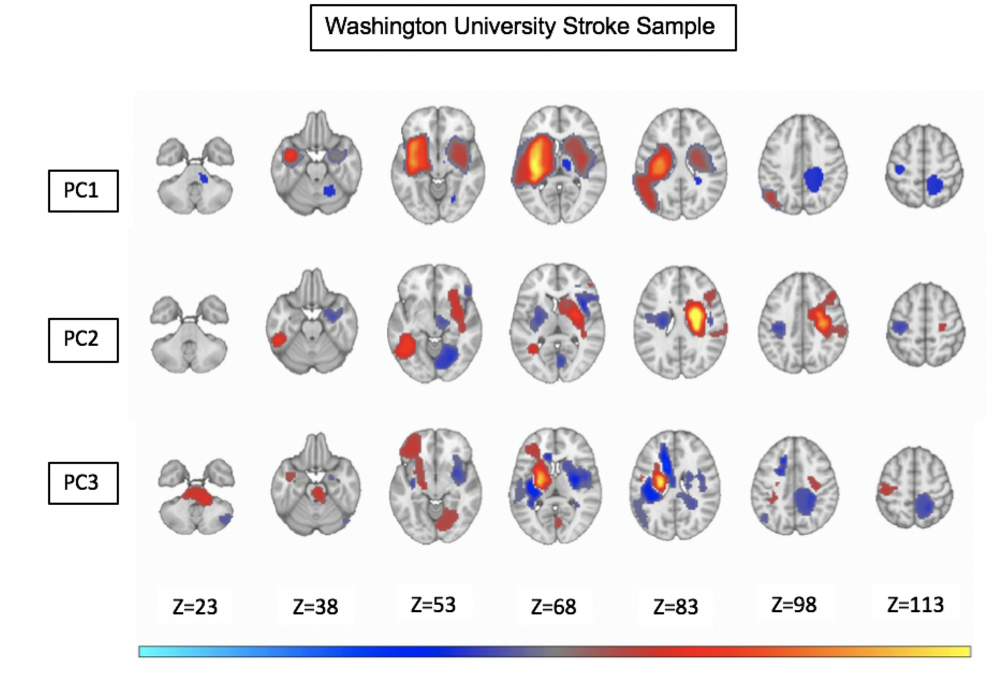

Figure 6- Ridge regression maps from the Washington University sample. Warm colors represent positive correlation between anatomical voxels and high PC values (i.e. high level of impairment in the corresponding domains). Cold colors represent negative correlation between anatomical voxels and high PC values. Anatomical overlay maps are shown for PC1, PC2 and PC3 scores, respectively (after Gaussian smoothing (variance = 1) and scaling within [-1,+1]; weights lower than 0.05 in absolute values are not shown).

164x115mm (144 x 144 DPI)

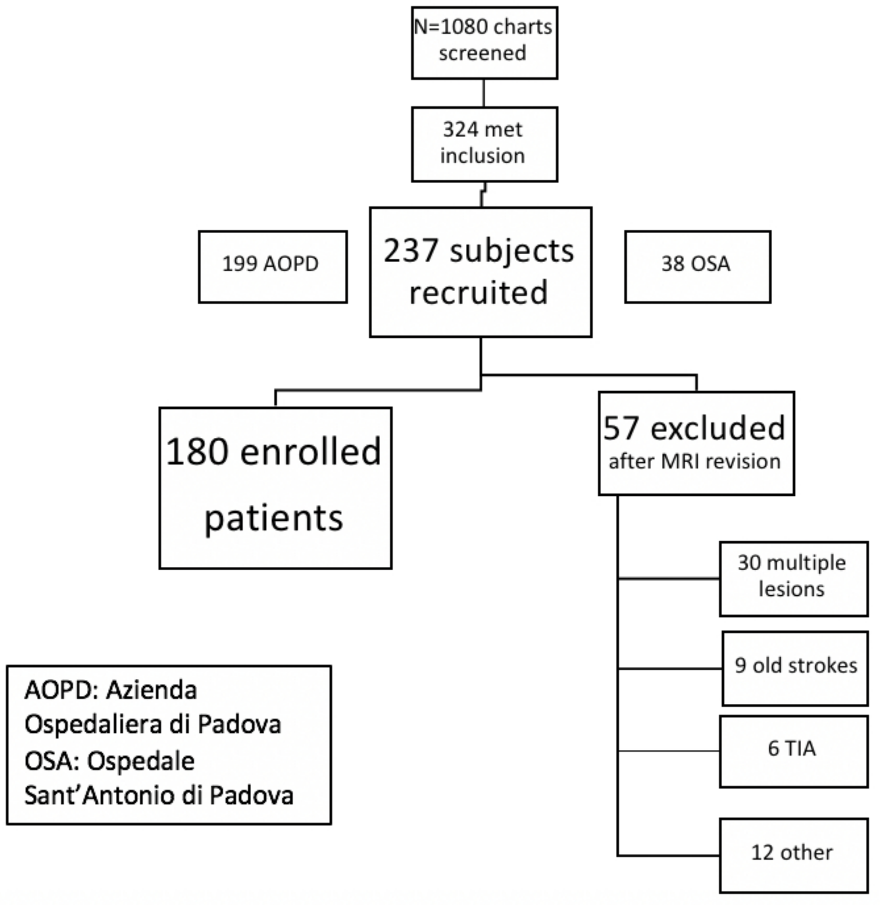

Figure S1

134x127mm (144 x 144 DPI)

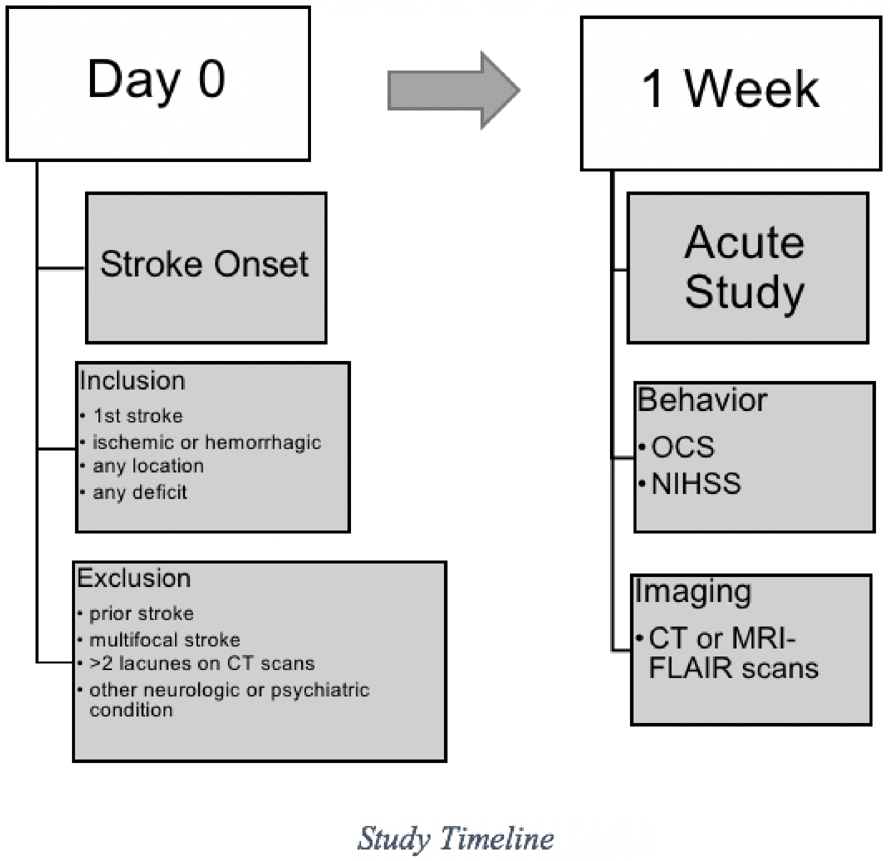

Figure S2

106x102mm (144 x 144 DPI)

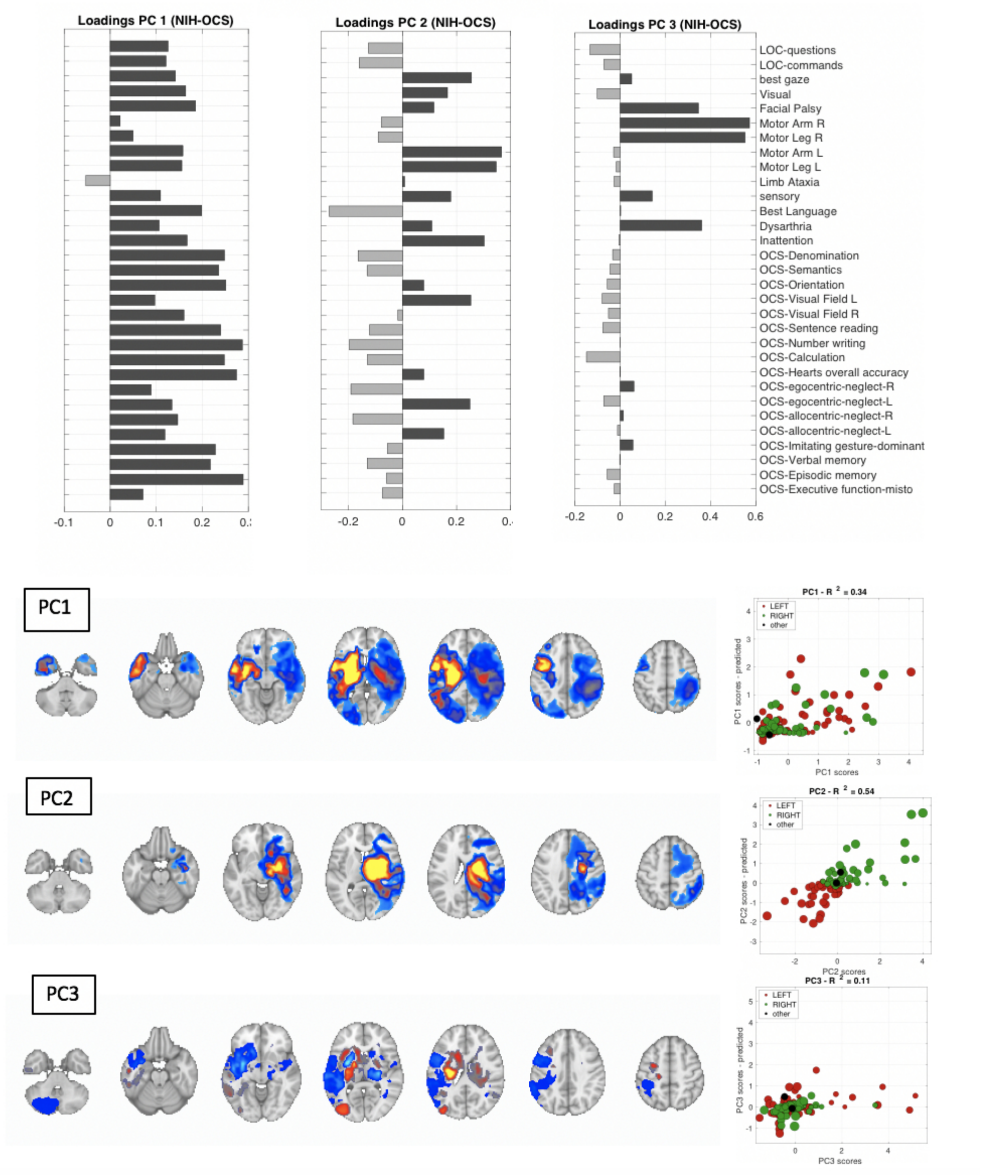

Figure S3

172x210mm (144 x 144 DPI)

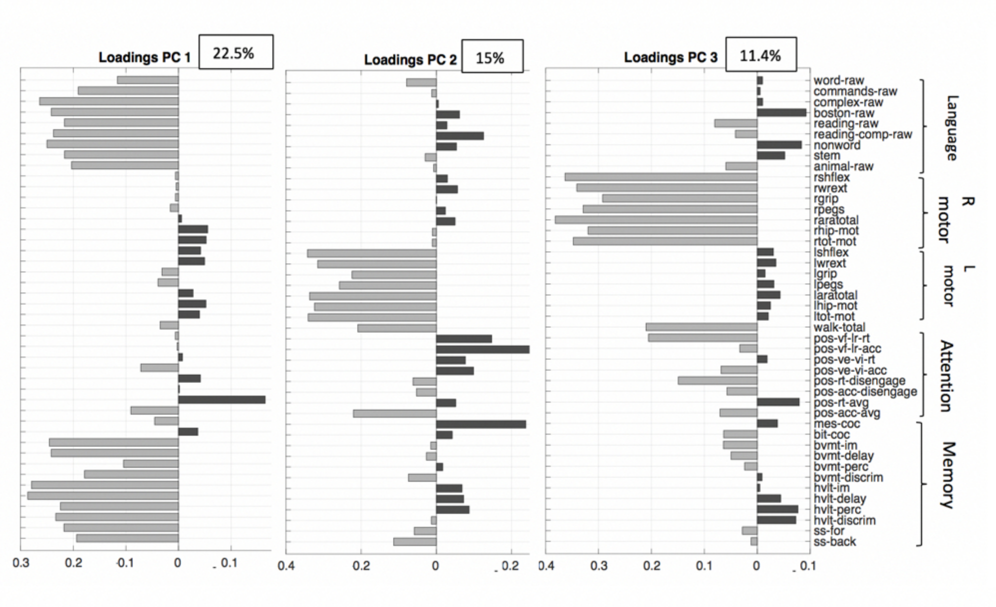

Figure S4

176x106mm (144 x 144 DPI)
