# Supplementary material for: A low-dimensional structure of neurological impairment in stroke
Source: Brain Commun. 2021 Jun 3;3(2):fcab119. doi: 10.1093/braincomms/fcab119 (PMC8204367; doi:10.1093/braincomms/fcab119)
Supplement: fcab119_Supplementary_Data [file fcab119_supplementary_data.zip › Supplementary_material.pdf]

Supplementary Figure 1. Enrollment Flowchart

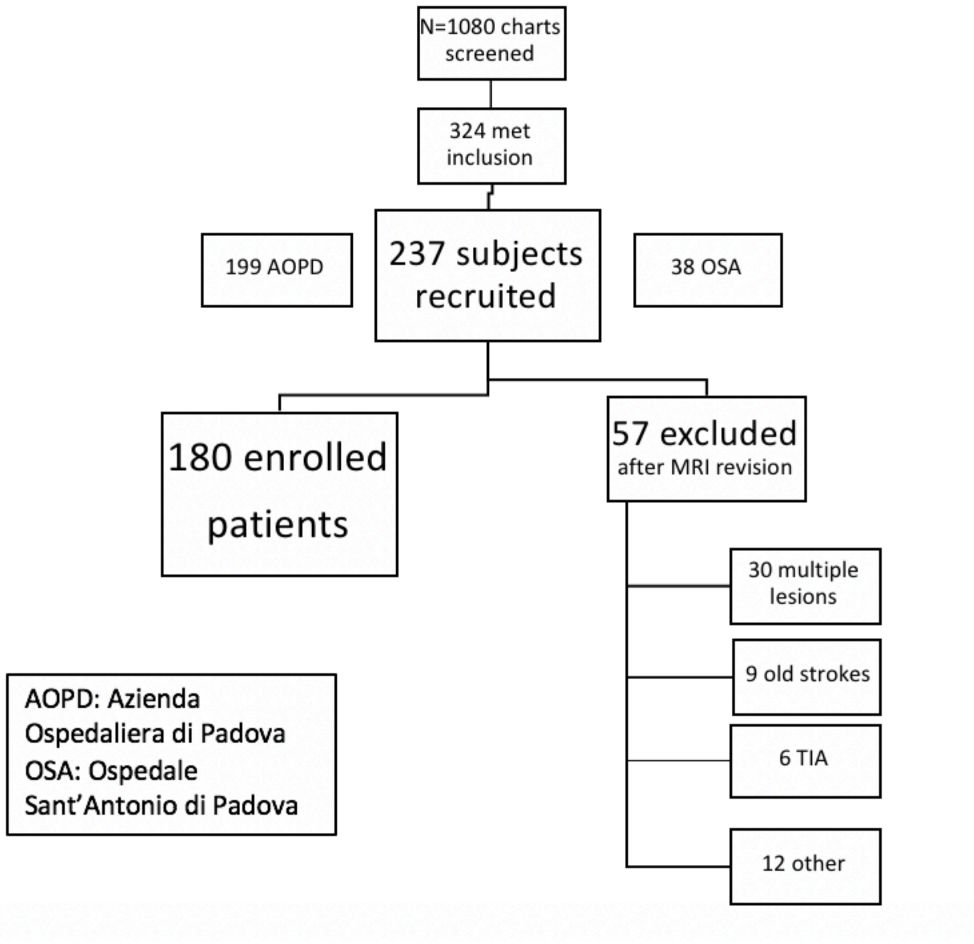

Supplementary Figure 2. Design of the study

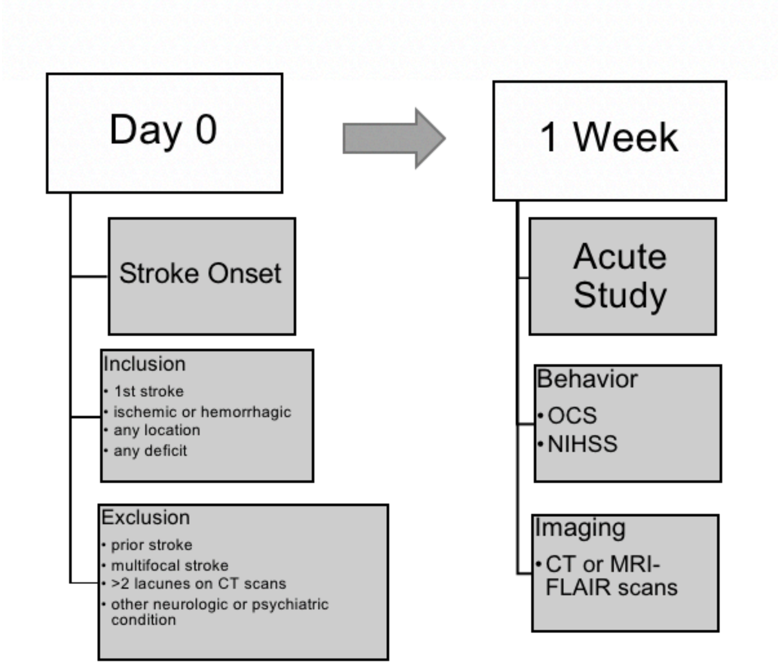

Supplementary Figure 3. Non-rotated Principal Component Analysis. Figure A) PCs (PC1-3) loading values for each subtest considered in our sample (total PCs variance explained=45%); Figure B) the most predictive anatomical structures (weights of the ridge regression) associated with each PC scores. The orange/yellow color scale indicates damaged voxels associated with low performance, whereas the blue/teal color scale indicates damaged voxels associated with high performance. Scatter plots on the right show the model explained different levels of variance for each factor (PC1: 34%, PC2: 54% and PC3: 11% respectively).

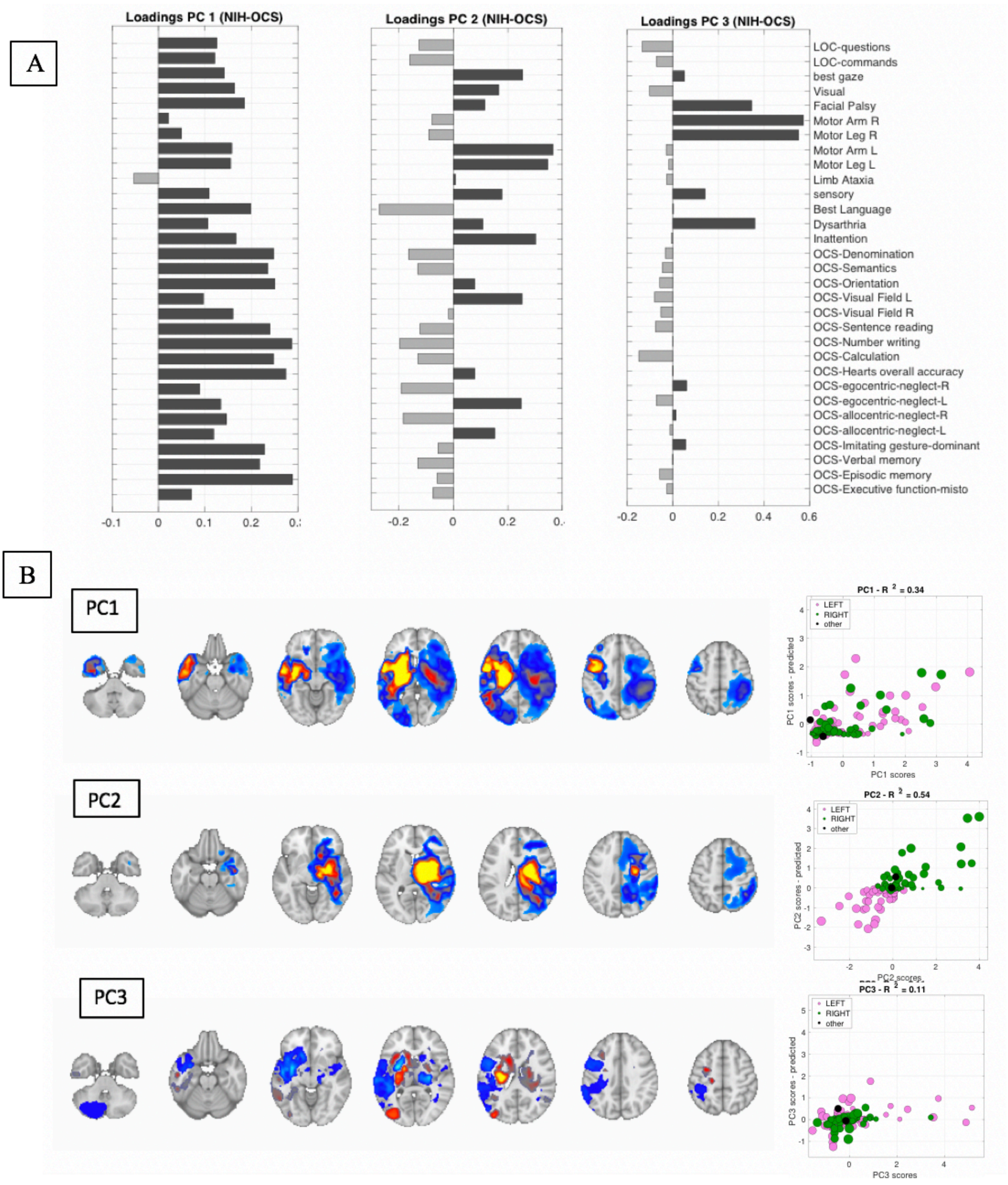

Supplementary Figure 4. Washington University cohort PCA scores. After z-scoring of behavioral scores, a principal component analysis (PCA) reduced the number of variables to describe the across subject variability of behavioral deficits (See Corbetta et al. Neuron 2015 for full list of tests).

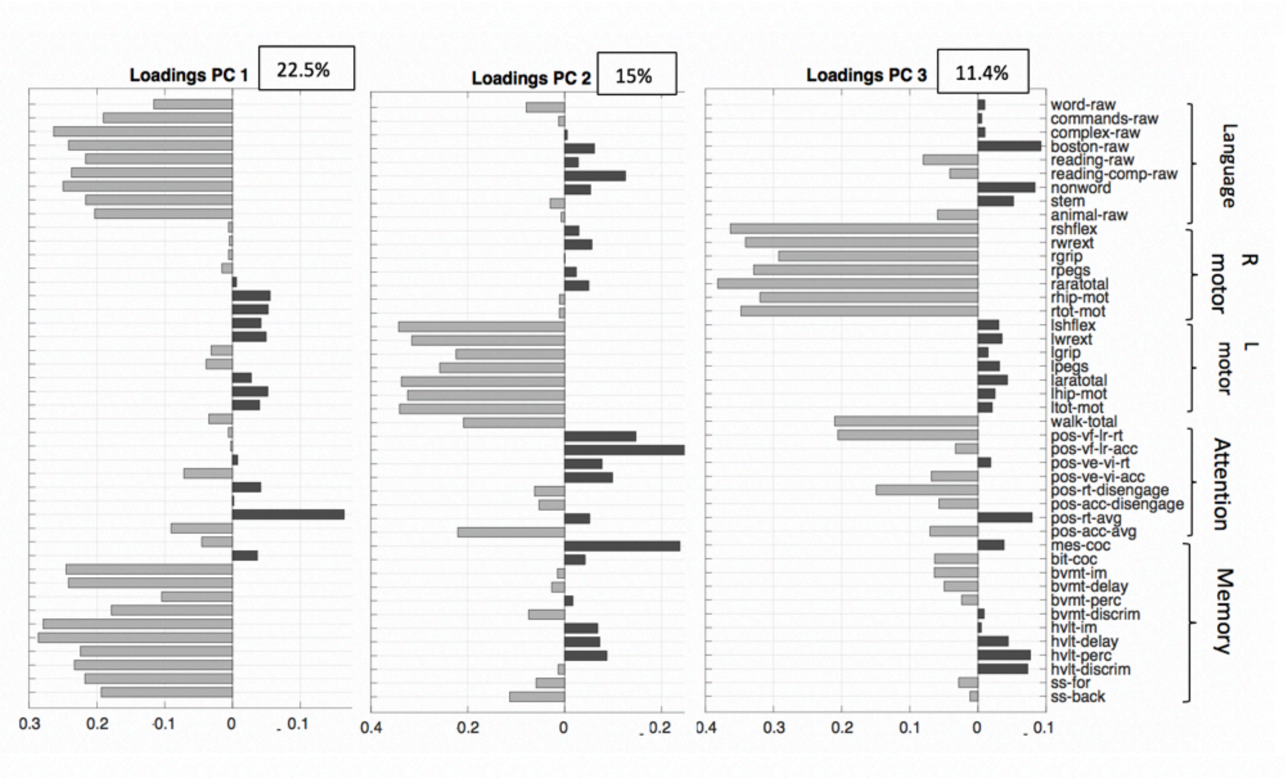

Supplementary Figure 5. Vascular territories and PC scores. A) We considered a mask of three MCA territories (deep branches, antero-superior branch, and postero-inferior branch) and we evaluated the percentage of each subject's lesion located in the three territories (on the left). We derived four clusters (Silhouette and Davies Bouldin indexes). Cluster 1: deep, antero-superior and postero-inferior branches. Cluster 2: deep branches. Cluster 3: postero-inferior branch. Cluster 4: antero-superior branch (on the right). B) Distribution of PC scores within each cluster from the Padova dataset. On the "y" axis PC scores (average ± confidence interval 95%) in each Cluster, on the "x" axis each Principal Component. Main effects between clusters for each PC assessed individually showed no significant difference. C) B) Distribution of PC scores within each cluster from the Washington University dataset. On the "y" axis PC scores (average ± confidence interval 95%) in each Cluster, on the "x" axis each Principal Component. Main effects between clusters for each PC assessed individually showed no significant difference.

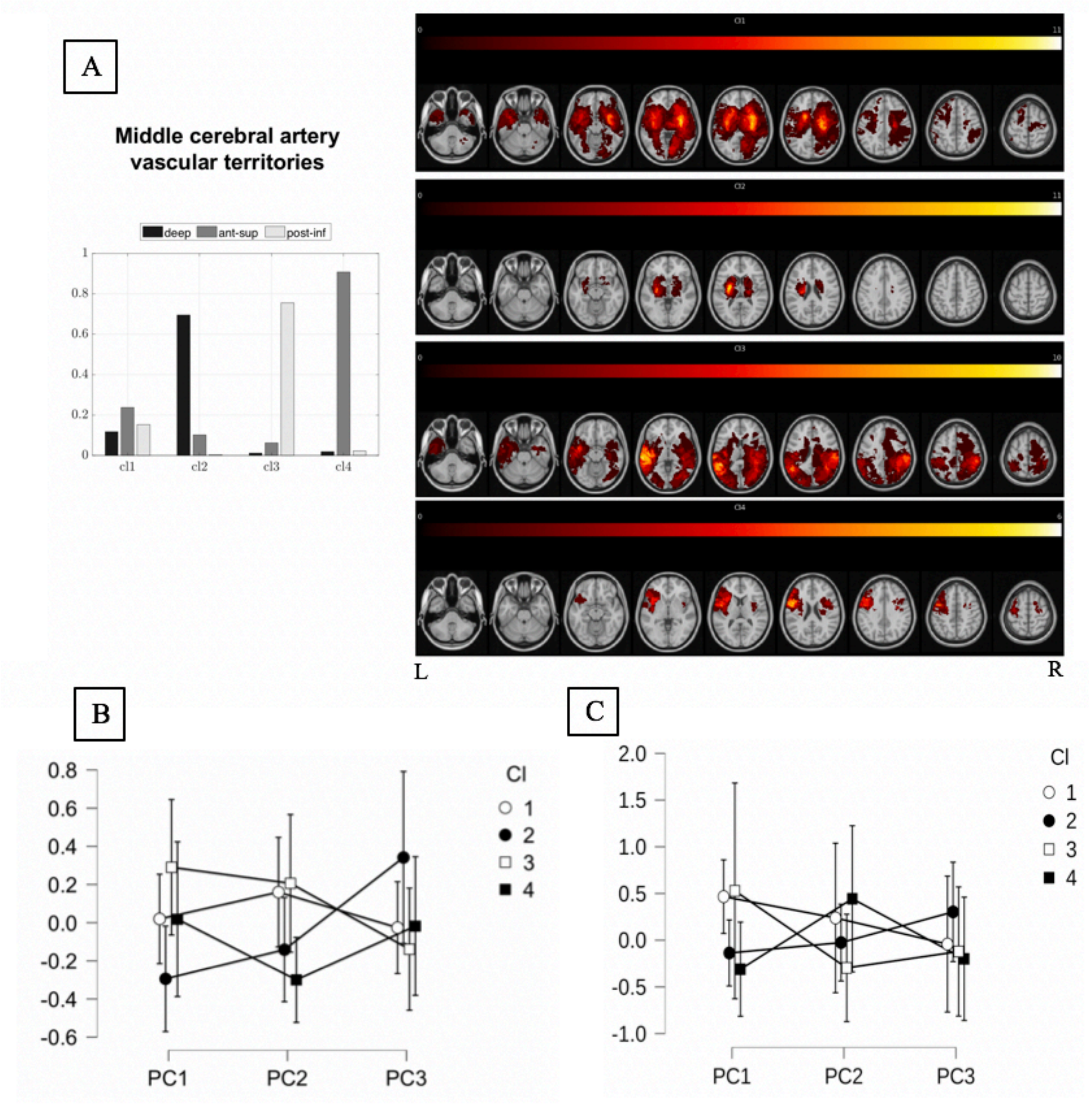

Supplementary Table 1. Demographics & Clinical Characteristics of the Sample.

Study sample	
**Age**	
18-30	1,5%
31-50	10,5%
51-70	29,5%
71 or more	58,5%
**Gender**	
Female	47%
Male	53%
**Education**	
Middle School or less	55%
High school	26%
Incomplete college	8%
College or post-graduate	11%
**Risk Factors**	
Hypertension	64%
Diabetes mellitus	24%
Coronary artery Disease	12%
Atrial Fibrillation	15%
Smoking	23%
**Stroke Severity (NIHSS)**	
Mild 0-6	58%
Moderate 7-15	28%
Severe 16-42	14%
**Stroke Symptoms**	
Neglect	20%
Aphasia	35%
Motor Impairment	90%
**Lesion Side**	
Right Cerebral	40%
Left Cerebral	44%
Cerebellum/Midbrain	7.5%
Negative scan	9%
**Stroke Subtype**	
Ischemic	89%
Hemorrhagic	11%

Supplementary Table 2. Acute Reperfusion Therapy details. rtPA= recombinant tissue Plasminogen Activator; Tot. OCS= number of impaired OCS subtests (according to normative cut-offs).

**Stroke Sample (n=180)**	**N. of patients**	**Mean NIHSS score on admission**	**Mean NIHSS score at time NPSY testing**	**Mean total OCS score**
**No acute treatment**	104 (58%)	4.3	2.7	4.2
**Acute treatment**	76 (42%)	10.8	4.1	5.5
• **rtPA**	37 (20.4%)	7.7	2.8	4.2
• **Thrombectomy**	18 (10%)	12.8	6.4	7
• **rTPA+Thrombectomy**	21 (11.6%)	14.6	4.4	6.5

Supplementary Table 3. Neuroimaging lesion details. We describe the patient stroke etiology (i.e. ischaemic or hemorrhagic), lesion location (expert localization) and lesion volume in voxels (2x2x2mm) or ml.

Stroke ID	Stroke Etiology (1=ischemic;2=hemorrhagic)	Lesion location	Volume (in voxels of 2x2x2 mm)	Volume (in ml)
**1**	1	right basal ganglia	1060	8,48
**2**	2	left occipital	5232	41,856
**4**	1	right occipital	2911	23,288
**5**	2	left temporal	9444	75,552
**6**	1	right frontal	1625	13
**7**	1	left basal ganglia	6001	48,008
**8**	1	left superior temporal	6585	52,68
**10**	1	right posterior temporal	228	1,824
**11**	1	right corona radiata	300	2,4
**13**	1	right frontal	46	0,368
**14**	1	left frontal	2345	18,76
**15**	1	right basal ganglia	2256	18,048
**19**	2	left basal ganglia	98	0,784
**21**	1	left pons	168	1,344
**22**	1	right basal ganglia	343	2,744
**23**	1	right basal ganglia	401	3,208
**24**	1	left frontal	2015	16,12
**25**	1	right frontal	171	1,368
**27**	1	left frontal	5478	43,824
**28**	2	left temporo-parietal	1865	14,92
**29**	1	right basal ganglia	152	1,216
**30**	1	left frontal	22	0,176
**31**	1	left fronto-temporo-parietal	11428	91,424
**32**	1	left cerebellar	142	1,136
**33**	1	right parietal	11725	93,8
**34**	1	right superior pons	339	2,712
**35**	1	left parietal	22	0,176

**36**	1	right internal capsule	95	0,76
**37**	1	left basal ganglia	166	1,328
**38**	2	right basal ganglia	5348	42,784
**39**	1	left temporal and basal ganglia	11772	94,176
**41**	1	left basal ganglia	1019	8,152
**42**	1	left basal ganglia	22	0,176
**43**	1	right parietal	167	1,336
**44**	2	right basal ganglia	1188	9,504
**45**	1	left fronto-parietal	1698	13,584
**46**	2	left basal ganglia	2243	17,944
**48**	1	temporal	5529	44,232
**49**	1	left frontal	122	0,976
**50**	1	left internal capsule	39	0,312
**51**	1	left basal ganglia and temporal	3914	31,312
**52**	1	left frontotemporoparietal	7797	62,376
**53**	1	left frontal	4121	32,968
**54**	1	right parietal	3393	27,144
**55**	2	right basal ganglia	4049	32,392
**57**	1	left fronto parietal	3368	26,944
**58**	1	left basal ganglia	230	1,84
**59**	1	right frontal	22	0,176
**60**	1	right frontoparietal	1422	11,376
**61**	2	right occipital	6349	50,792
**62**	1	left basal ganglia	2232	17,856
**63**	1	left corona radiata	165	1,32
**64**	1	left cortico-subortical temporo-parietal	2980	23,84
**67**	1	right hippocampus	29	0,232
**68**	1	left frontal	452	3,616
**69**	1	left frontal	1188	9,504

**71**	1	left cerebellar	6149	49,192
**72**	1	right hippocampus	402	3,216
**74**	1	right frontal	121	0,968
**75**	1	left frontal	687	5,496
**76**	1	right occipital	2877	23,016
**78**	1	right pons	33	0,264
**79**	1	left frontal	27	0,216
**81**	1	right subcortical	14	0,112
**85**	1	right frontal	546	4,368
**87**	1	right frontal	158	1,264
**90**	1	right cerebellar	45	0,36
**91**	1	left parieto temporal	1447	11,576
**92**	1	right thalamus	34	0,272
**93**	1	right subcortical	2479	19,832
**94**	2	right putamen	17723	141,784
**95**	1	right cerebellar	336	2,688
**96**	1	left temporal	5200	41,6
**98**	1	right internal capsule	220	1,76
**99**	1	left subcortical	774	6,192
**101**	1	left occipital	2920	23,36
**102**	1	right corona radiata	130	1,04
**104**	1	right frontal	129	1,032
**106**	1	right parietal	37344	298,752
**107**	1	left temporo-hyppocampal	3760	30,08
**108**	1	right frontal	18	0,144
**109**	2	right fronto-parietal	29679	237,432
**110**	1	left frontal	44	0,352
**115**	1	left frontal	529	4,232
**117**	1	left frontal	11424	91,392
**118**	1	left frontal	2528	20,224
**120**	1	right parietal	10316	82,528

**121**	1	left tempor-parieto-occipital	1734	13,872
**122**	1	left frontal	6851	54,808
**124**	1	left fronto-parietal	25	0,2
**125**	1	right subcortical	57	0,456
**126**	1	right thalamus	169	1,352
**127**	1	left temporoparietal	757	6,056
**129**	1	right occipital	3125	25
**130**	1	left basal ganglia	957	7,656
**132**	1	left subcortical	1695	13,56
**133**	1	right frontal	623	4,984
**135**	2	left basal ganglia	19443	155,544
**136**	1	left frontal	852	6,816
**137**	1	left fronto-temporal	1911	15,288
**138**	1	right parietal	1853	14,824
**139**	1	right fronto-temporal	20114	160,912
**140**	1	left frontal	228	1,824
**141**	1	left corona radiata	442	3,536
**142**	1	left pons	334	2,672
**143**	1	right temporal	1234	9,872
**144**	1	right thalamus	270	2,16
**145**	1	rght temporal	19782	158,256
**146**	1	left thalamus	28	0,224
**148**	1	left thalamus	19	0,152
**149**	1	left thalamus	979	7,832
**150**	2	right basal ganglia	3160	25,28
**151**	1	right frontal	729	5,832
**152**	1	left front-parietal	186	1,488
**154**	1	right basal ganglia	752	6,016
**156**	1	left cerebellar	168	1,344
**157**	1	right basal ganglia	6321	50,568
**159**	1	right subcortical and basal ganglia	5848	46,784

**161**	1	right temporo-occipital	1329	10.632
**163**	1	Left cerebellar hemisphere	6981	55.848
**165**	1	Left caudate and internal capsule	499	3.992
**166**	1	Left caudate and anterior internal capsule	316	2.528
**167**	1	Left precentral gyrus	3011	24.088
**168**	1	Left temporo-parietal	3253	26.016
**169**	1	Left capsular	737	5.896
**171**	1	Left frontal cortico-subcortical	647	5.176
**172**	1	Left capsular	346	2.768
**173**	1	Left fronto-temporal	21699	173.592
**175**	2	Right occipital	7061	56.488
**178**	2	Left nucleo capsular	2440	19.52
**181**	1	Left parietal and internal capsule	730	5.84
**182**	1	Right occipito-mesial	3333	26.664
**184**	2	Right thalamus	331	2.648
**185**	1	Left fronto-parietal	652	5.216
**186**	1	Right corona radiata	1437	11.496
**187**	2	Right parieto-occipital	4471	35.768
**188**	1	Left tempo-parietal	2486	19.888
**190**	1	Right parietal	1122	8.976
**194**	1	Left fronto-parietal	758	6.064
**195**	1	Left prefrontal gyrus	23	0.184
**196**	1	left temporo-parietal	20544	164.352
**198**	1	Left fronto-parietal	62	0.496
**199**	1	Left frontal	534	4.272
**200**	1	Left corona radiata	113	0.904
**202**	1	Left frontal	7650	61.2
**203**	1	Left fronto-opercular	1107	8.856

**205**	1	Left temporal	3835	30.68
**208**	1	Left caudate and putamen	2747	21,976
**212**	1	Left frontal and callosal body	279	2.232
**215**	2	Left cerebellar	3618	28.944
**216**	1	Right occipital	2538	20.304
**221**	1	Left fronto-parietal	10325	82.6
**223**	1	Left frontal	4188	33.504
**224**	1	Left temporo-parietal	3097	24.776
**225**	1	Left insular	3035	24.28
**226**	1	Left posterior insular	633	5.064
**227**	1	Right nucleo-capsular	8627	69.016
**233**	1	Right fronto-parietal	17483	139.864
**235**	1	Medial thalamus	2731	21.848
**236**	1	Right precentral gyrus	799	6.398
**237**	1	Right cerebellar	742	5.936
**Average**			3357	26.0

Supplementary Table 4. Harvard-Oxford Cortico-Subcortical Parcellation. We computed the mean, standard deviation and maximum values (expressed as a percentage) of parcels affected by the lesions of our sample from the Harvard-Oxford atlas.

ROI (Subcortical Mask)	Mean % damaged parcels	Std	Max
**Left Cerebral White Matter**	2.00	2.09	13.69
**Left Cerebral Cortex**	0.93	1.59	13.10
**Left Lateral Ventricle**	2.27	1.30	7.14
**Left Thalamus**	1.78	1.31	5.95
**Left Caudate**	5.50	1.88	9.52
**Left Putamen**	6.95	2.63	13.10
**Left Pallidum**	5.11	1.42	8.93
**Brainstem**	0.23	0.41	2.38
**Left Hippocampus**	1.46	1.11	4.17
**Left Amygdala**	2.76	1.40	5.95
**Left Accumbens**	1.36	1.07	3.57
**Right Cerebral White Matter**	2.73	1.97	11.90
**Right Cerebral Cortex**	0.78	1.31	10.12
**Right Lateral Ventricle**	2.84	0.99	6.55
**Right Thalamus**	2.65	1.63	7.74
**Right Caudate**	4.14	1.22	7.14
**Right Putamen**	6.24	1.91	10.71
**Right Pallidum**	5.31	1.38	7.74
**Right Hippocampus**	1.33	1.20	4.76
**Right Amygdala**	1.31	1.34	6.55
**Right Accumbens**	1.55	0.86	3.57

ROI (Cortical Mask)	Mean % damaged parcels	Std	Max
**Frontal Pole**	0.18	0.44	5.36
**Insular Cortex**	7.00	1.97	13.10
**Superior Fronmtal Gyrus**	0.31	0.44	2.98
**Middle Frontal Gyrus**	1.02	1.11	6.55
**Inferior Frontal Gyrus pars triangularis**	1.56	1.21	4.76

**Inferior Frontal Gyrus. pars opercularis**	2.62	1.72	7.14
**Precentral Gyrus**	1.15	1.39	7.74
**Temporal Pole**	1.12	1.26	8.33
**Superior Temporal Gyrus. anterior division**	2.66	2.04	8.33
**Superior Temporal Gyrus. posterior division**	3.07	2.12	8.33
**Middle Temporal Gyrus. anterior division**	1.12	1.14	5.95
**Middle Temporal Gyrus. posterior division**	1.83	1.78	7.74
**Middle Temporal Gyrus. temporooccipital part**	2.37	1.74	7.74
**Inferior Temporal Gyrus. anterior division**	0.66	0.81	3.57
**Inferior Temporal Gyrus. posterior division**	0.48	0.68	4.17
**Inferior Temporal Gyrus. temporooccipital part**	0.52	0.83	4.17
**Postcentral Gyrus**	0.70	1.02	6.55
**Superior Parietal Lobule**	0.91	0.82	5.36
**Supramarginal Gyrus. anterior division**	1.78	1.42	5.36
**Supramarginal Gyrus. posterior division**	2.43	1.64	7.74
**Angular Gyrus**	2.30	1.59	7.14
**Lateral Occipital Cortex. superior division**	1.08	1.18	5.95
**Lateral Occipital Cortex. inferior division**	1.17	1.44	6.55
**Intracalcarine Cortex**	2.25	1.68	6.55
**Frontal Medial Cortex**	0.10	0.31	2.38
**Juxtapositional Lobule cortex (formerly Supplementary motor cortex)**	0.20	0.33	2.38
**Subcallosal Cortex**	0.44	0.65	4.17
**Paracingulate Gyrus**	0.14	0.28	2.38
**Cingulate Gyrus. anterior division**	0.46	0.62	4.76
**Cingulate Gyrus. posterior division**	0.49	0.75	4.76
**Precuneous Cortex**	0.65	0.94	5.36
**Cuneal Cortex**	1.30	1.03	5.36
**Frontal Orbital Cortex**	1.54	1.51	7.14
**Parahippocampal Gyrus. anterior division**	0.82	1.14	6.55
**Parahippocampal Gyrus. posterior division**	0.86	1.07	4.76

**Lingual Gyrus**	1.12	1.36	6.55
**Temporal Fusiform Cortex. anterior division**	0.52	0.81	3.57
**Temporal Fusiform Cortex. posterior division**	0.74	0.91	4.76
**Temporal Occipital Fusiform Cortex**	0.68	0.86	4.76
**Occipital Fusiform Gyrus**	0.95	1.17	7.14
**Frontal Operculum Cortex**	4.08	1.29	7.14
**Central Opercular Cortex**	4.35	1.48	9.52
**Parietal Operculum Cortex**	3.68	1.38	8.33
**Planum Polare**	4.86	1.56	11.31
**Heschl's Gyrus (inculdes H1 and H2)**	4.91	1.52	8.93
**Planum Temporale**	4.09	1.30	9.52
**Supracalcarine Cortex**	1.69	1.51	5.36
**Occipital Pole**	0.67	0.87	5.95

Supplementary Methods. Ridge regression procedure.

RR adds a L2-normalization term to the ordinary linear regression, in order to assign small coefficients to unimportant predictors, thus preventing data overfitting, and improving generalization for new data. We used the binary matrix of voxels damage as predictor (for each subject and for each voxel, the entry of the matrix is set to 1 if the voxel is lesioned and 0 otherwise). Due to computational issues, instead of considering all 902,629 2-mm^3 voxels, we first applied a spatial PCA, and we used as regressors only the first N_p PCs, which explained at least the 95% of the variance. Besides resolving the dimensionality problem, the PCA step had also the purpose to transform the original binary matrix into a set of continuous predictors. Thus, for each of the behavioral PCs, we estimated the model weights vector $\boldsymbol{\beta}$ as:

$$\boldsymbol{\beta} = (\boldsymbol{X}^{\mathrm{T}}\boldsymbol{X} + \lambda\boldsymbol{I})^{-1}\boldsymbol{X}^{\mathrm{T}}\boldsymbol{y}\,,$$

where $\boldsymbol{X} \in \mathbb{R}^{N_s \times N_p}$ is the predictors matrix (N_s is the number of subjects and N_p is the number of selected spatial PCs), after z-scoring w.r.t. the whole matrix; $\boldsymbol{X}^{\mathrm{T}} \in \mathbb{R}^{N_p \times N_s}$ is the transpose of $\boldsymbol{X}$, $\boldsymbol{y} \in \mathbb{R}^{N_s}$ is the vector of the outcome variable to be predicted (i.e. the selected behavioral PC score, after z-scoring), $\boldsymbol{I} \in \mathbb{R}^{N_p \times N_p}$ is the identity matrix of dimension N_p, and $\lambda \in \mathbb{R}$ is the regularization parameter, optimized as follows.

For each of the three RR models, the regularization parameter λ was optimized by identifying a value within $[10^{-5},\ 10^{5}]$, with 200 logarithmic steps. For each of these 200 values of λ, each RR model was trained and tested using a leave-one-out cross validation loop (LOOCV), which uses $N_s - 1$ training data to estimate the model weights and applies them to the left-out patient to predict his behavioral score. The optimal λ (λ_{opt}) value was the one that minimized the prediction error over the training set, and the predictions obtained with λ_{opt} were considered as the model predictors $\hat{\boldsymbol{y}}$.

Model accuracy was assessed through R^2:

$$R^2 = 1 - \frac{\sum_{i=1}^{N_s}(y_i - \hat{y}_i)^2}{\sum_{i=1}^{N_s}(y_i - \bar{y})^2}, \text{ where } \bar{y} = \frac{1}{N_s}\sum_{i=1}^{N_s} y_i,$$

where y_i represent the i-th element of vector $\boldsymbol{y}$. The statistical significance was estimated through a permutation test, with $N = 10{,}000$ iterations. For each iteration, behavioral scores were randomly permuted across subjects, and the LOOCV with λ optimization was used to fit the RR model to the randomized scored. The p-value for the observed R^2 was defined as the probability of the R^2 of the

randomized dataset to be larger than the observed R^2. Only models with p-values less than 0.05 were considered statistically able to predict the behavioral scores. To obtain the optimal set of RR model weights $\boldsymbol{\beta}$, the weights obtained for each LOOCV loop at λ_{opt} were averaged across the N_s loops. The distribution of weights obtained with the permutation test was used as null distribution to select statistically significant weights. Only the β_i's that fall at the left or right ends (2.5%) of the tails of the distribution were considered significant. These selected weights were projected to the brain to display a map of the most predictive lesioned voxels. Finally, Gaussian smoothing (variance = 1) and scaling within $[-1, +1]$ was applied on the maps. Weights lower than 0.05 in absolute values were not shown.
