# Supplementary material for: A low-dimensional structure of neurological impairment in stroke
Source: Brain Commun. 2021 Jun 3;3(2):fcab119. doi: 10.1093/braincomms/fcab119 (PMC8204367; doi:10.1093/braincomms/fcab119)
Supplement: fcab119_Supplementary_Data [file Original Submission-Author's Response.pdf › Original Submission-Author's Response.pdf]

Padua, 18/03/2021

To the Editor: Prof. Karen Ritchie

Re: **A low dimensional structure of neurological impairment in stroke** by Bisogno et al.

We thank the editor Prof. Ritchie for considering our paper for publication. We answer the reviewer's comments below. We are willing to have all comments, on our paper and our response, published as supplementary data with the paper if it is accepted.
We take the opportunity to thank the reviewers for the useful comments that have certainly improved the quality of the original manuscript.

Reviewer:1

1. Overall this article is well-done and has several strengths. This includes a large sample size, a prospective design with well-defined inclusion and exclusion criteria that match the prior 2015 cohort, a strong statistical design for dealing with behavior and lesion-behavior mapping, and a cross-cohort analysis to further support the main findings. The findings are directly relevant to ongoing efforts in the field to develop better prognostic tools for stroke outcomes.
I don't see any major flaws in the study design. The methods are well suited to address the questions being asked and the conclusions are aligned with the results.
We thank the reviewer for the positive comments.

2. I have a few questions and points of clarification that may help to improve the final manuscript. In the abstract the authors report that: 'post-stroke neurological impairment is best described by a few groups of correlated deficits across different domains that relate to the dysfunction of distributed brain networks.' It might be more accurate to say that recent studies have shown that much of the variance in post-stroke outcomes can be explained by a few groups of correlated deficits. It seems premature to conclude that this is the 'best' way to characterize post-stroke neurological impairment.
The abstract has been modified following the reviewer's suggestion: "Recent studies indicate that, at the population level, post-stroke neurological impairments cluster in three sets of correlated deficits across different behavioral domains" (see lines 4-6, page 2).

3. The authors report that neurological deficits relate to widespread network impairment rather than damage to a specific area or vascular territory. This statement appears to be largely made based on prior work and not the current analysis. More details supporting this claim would be helpful. Given the significant lesion-symptom mapping results in this analysis and the similarity to prior results (Corbetta, 2015) it appears these neurological deficits are reliably related to lesion location, even if the localization is regional as opposed to specific structures or specific vascular territories. The authors don't evaluate this topic of specific vascular distributions directly as far as I can tell. Are the authors confident that certain branches of the MCA are not associated with more pronounced deficits in the three major components?
The point about lesion location in relation to vascular territories is now discussed (see lines 21-34 page 15; 1-2, page 16). Following the reviewer's suggestion, we now examine more closely the issue of vascular territory in relation to the behavioral components found in the study. Specifically, we tested whether lesions in different vascular territories (e.g. deep vs. superficial branches of MCA) cause different profiles of deficits based on the principal component behavioral scores. First, we computed the percentage of overlap of each lesion with a mask of three vascular territories (deep branches, anterior-superior branch, and posterior-inferior

branch). Second, we clustered these groups of lesions based on their location within the vascular territories and selected the optimal number of clusters through Silhouette and Davies Bouldin indexes. Cluster 1 included patients with lesions in deep, antero-superior, and postero-inferior MCA branches. Cluster 2 included patients with lesions in the deep branches of MCA. Cluster 3 included patients with lesions in the postero-inferior branch of MCA. Finally, cluster 4 corresponded to lesions in the antero-superior branch of MCA (Figure 1-Supplementary Fig. 6A in the manuscript). We used a mixed measures ANOVA, with PC (PC1-PC3) as within-subjects factor and clusters as between-subjects factor, to test the interaction effect of PC scores and anatomical clusters. The results showed a significant factor x cluster interaction effect (F=2.48, p = 0.024) without a significant main effect for PC or cluster. The interaction effect was driven by different patterns of PC scores between cluster 2 and 3 (F = 5.008, p= 0.04, Bonferroni corrected for 6 multiple comparisons). This interaction shows that PC1 scores are stronger in cluster 3 given the cortical/perisylvian distribution, while PC3 scores are higher in cluster 2 given the subcortical location (Figure 2 - Supplementary Fig. 6B in the manuscript). We performed the same analysis on the subset of patients of the Washington University dataset (n=67). We found no interaction effect or main effects between PC scores and vascular clusters (Fig.3 - Supplementary Fig.6C in the manuscript). These results suggest that MCA lesion location is not associated with more or less pronounced deficits. However, vascular territories can influence the overall pattern of behavioral deficits (i.e. interaction effect) depending on their similarity with the topographical correlates related to the axes of behavioral impairment we identify in this study. We added this analysis in our Material and Methods section (Vascular territory control Analysis- lines 1-10, page 7), in our Results section (Control for vascular distribution-lines 4-18, page 11), in Supplementary data (lines 1-12, page 34) and discussed this point in the discussion (see lines 21-34 page 15, lines 1-2 page 16).

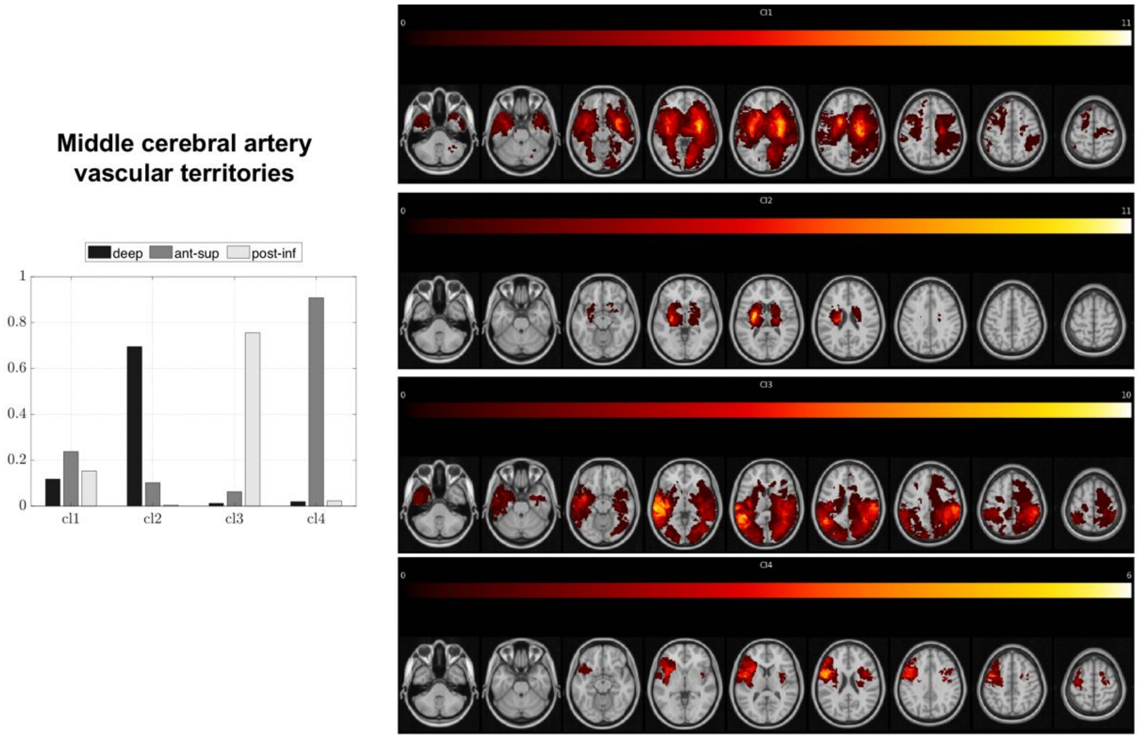

Figure 1. *Patients with lesions in different MCA vascular branches (Padova dataset).*

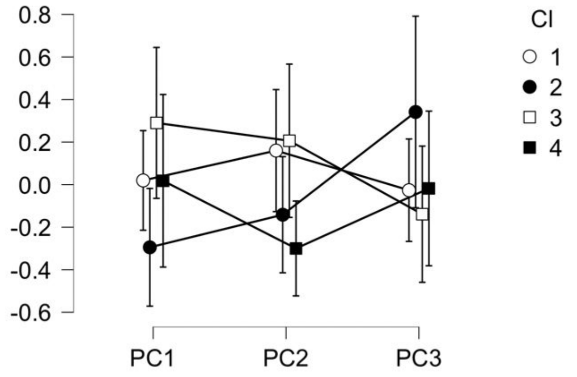

Figure 2. *Distribution of PC scores within each cluster from the Padova dataset. On the "y" axis PC scores (average $\pm$ confidence interval 95%) in each Cluster, on the "x" axis each Principal Component. Main effects between clusters for each PC assessed individually showed no significant difference.*

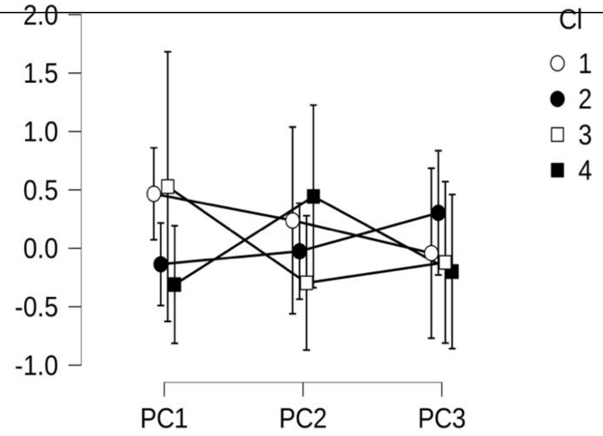

Figure 3. *Distribution of PC scores within each cluster from the Washington University dataset. On the "y" axis PC scores (average $\pm$ confidence interval 95%) in each Cluster, on the "x" axis each Principal Component. Main effects between clusters for each PC assessed individually showed no significant difference.*

5. The introductory comments appear to draw a contrast between the historical identification of stroke syndromes and the more recent elucidation of low dimensional clusters of correlated stroke symptoms. These newer PCA results, in my view, don't invalidate or diminish the existence of lesion syndromes. The two approaches don't seem diametrically opposed. Isn't it the case that these two broad strategies just describe stroke symptoms with different levels of granularity, with the low dimensional clusters explaining a large portion of the variance in outcomes, which is likely to be useful in some research and clinical applications?

This issue was extensively discussed in Corbetta et al. 2015, Neuron; ibidem 2018, Cortex. Here we include a sentence at the end of the discussion where we sum up the main points cycling back to the introductory contrast noted by the reviewer (see lines 4-6, page 16).

We think that our and others' recent work show that neurological deficits post-focal lesions are more accurately described by correlated deficit components rather than the collection of individual syndromes as in traditional neurological teaching. If pure syndromes were common, then they would show up across patients in the PCA. As the reviewer suggests, pure isolated neurological syndromes may exist in isolated cases and for small lesions (levels of granularity). This is an empirical question that we are trying to answer in separate work.

6. The authors apply a spatial PCA that captures 95% of the variance in the matrix of voxels damaged. I would like to have more details about the result of this data reduction strategy. Does this eliminate some regions with minimal lesion overlap (e.g. cerebellum, brainstem) from the multivariate model, or does it simply increase the size of smallest units entering the model?
The spatial PCA reduces the number of variables, while preserving as much information as possible in terms of variance over subjects. In other words, the first components are evaluated such as they show the largest differences among subjects, while the last ones represent a combination of variables that are more similar among subjects. For this reason, the applied spatial PCA over lesions captures the 95% of the inter-subjects' variance, eliminating those voxels that explain only 5% of variance, i.e. more similar among subjects (not lesioned in any subjects or lesioned in almost all subjects). The comparison of the original lesion PCA frequency map (Fig.4) with the difference before-after PCA (Fig.5) shows that the PCA removed lesion components common across subjects, hence not changing fundamentally the lesion topography distribution.

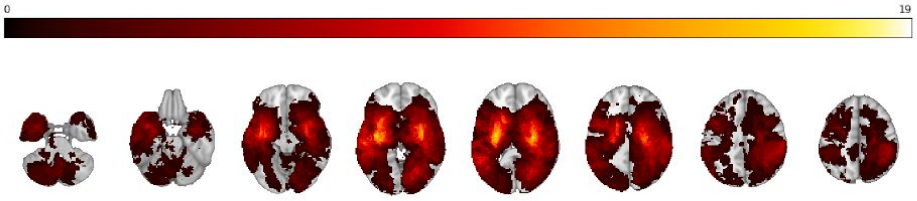

Figure 4. *Lesion frequency map after PCA (95% variance retained)*

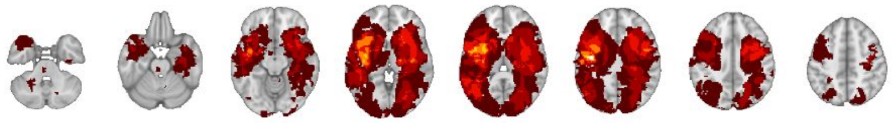

Figure 5. *Lesion frequency map difference (before – after PCA)*

7. What were the numbers of MRI versus CT scans?
130 MRI scans (72%), 50 CT scans (38%). We added this data in Procedures section line 1, page 5.

8. The predictive models perform less accurately for small lesions. Is this because these small lesions tend to occur in statistically underpowered regions outside the MCA distribution (brainstem. cerebellum), or does it reflect a more intact ability to compensate for deficits following a small lesion?

We thank the reviewer for this question that gave us the opportunity to examine in detail and elaborate on the effect of lesion size and location on the model's performance. To answer this question, we implemented three different analysis. (1) At first, to test whether small lesions negatively affected the model's performance, we evaluated the Pearson's correlation coefficients between the lesions volume and the relative estimation error of each PC separately (evaluated as $\frac{y-y}{|y|}$, where y is the real value of each behavioral PC, and y is the predicted behavioral PC). This analysis found that there was no significant correlation between lesion volume and prediction error (PC1: $\rho = 0.14$, p = 0.12; PC2: $\rho = -0.02$, p = 0.80; PC3: $\rho = -0.04$, p = 0.67). (2) As a second step, we tested whether the removal of small lesions would increase the R^2 of the RR models. To do so, without re-running the RR estimation, we evaluated the R^2 of each PC after removing one subject at a time in the order of increasing lesion volume. From this result (see Figure 6), we can see that model performance on PC1 and PC3 were not affected by small lesions, as their removal did not increase the R^2. For PC2, only a small improvement was obtained. Analyses (1) and (2) suggest that the predictive model did not perform less accurately for small lesions. We corrected accordingly in the main text (see lines 23-24, page 9; lines 19, page 10). (3) Finally, for each behavioral PC, we considered the 5% (7 subjects) and the 10% of subjects (14 subjects) with the largest relative estimation error, and we derived the frequency maps of their lesions (see Figures 7-8). These lesions occurred in commonly affected regions (deep, superficial MCA) ruling out an explanation based on relative frequency of occurrence. For confirmation, we evaluated the Pearson's correlation coefficient between the relative estimation error of each PC and the percentage of the lesion of each subject that were non-overlapped with any other lesion. Accordingly, we did not find any significant correlation (PC1: $\rho = 0.002$, p = 0.98; PC2: $\rho = -0.06$, p = 0.51; PC3: $\rho = -0.07$, p = 0.43).

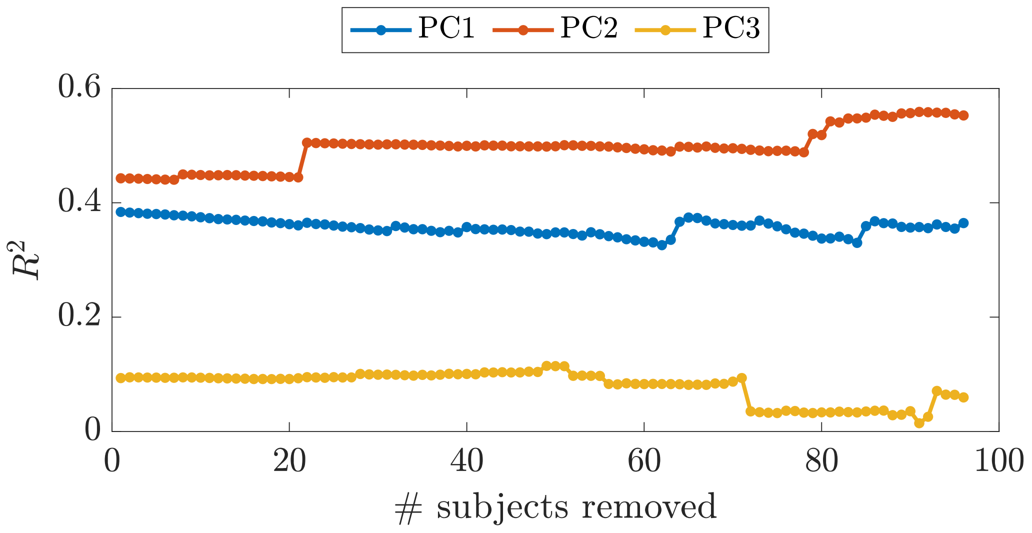

Figure 6. *Model performance after removing one subject at a time, in increasing lesion volume order.*

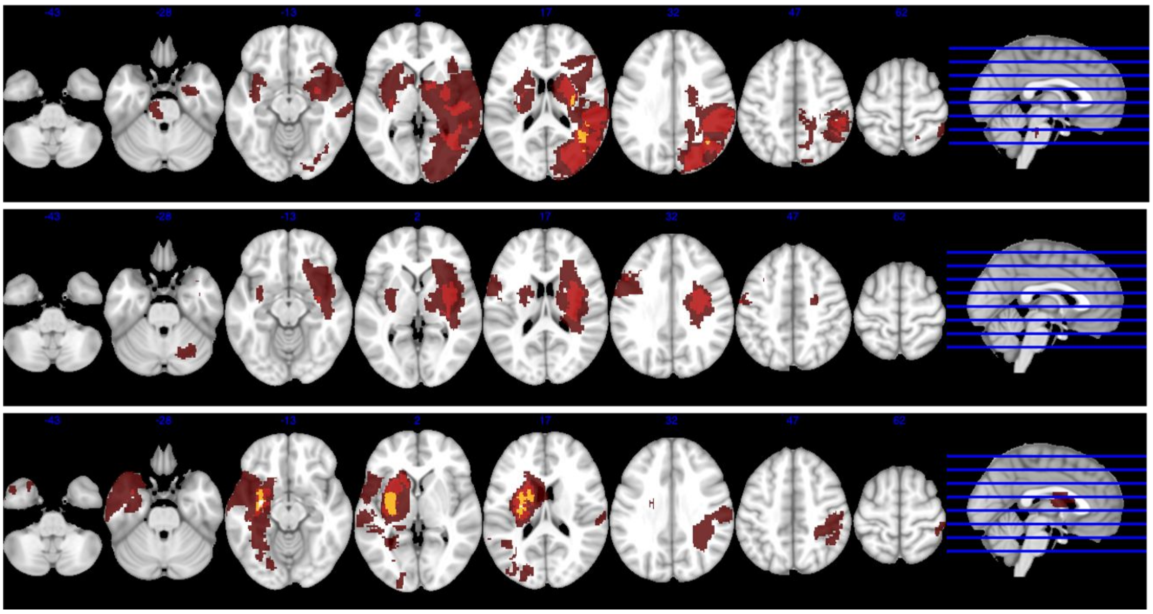

Figure 7. *Frequency maps of lesions related to the 5% (7 subjects) with largest relative estimation errors.*

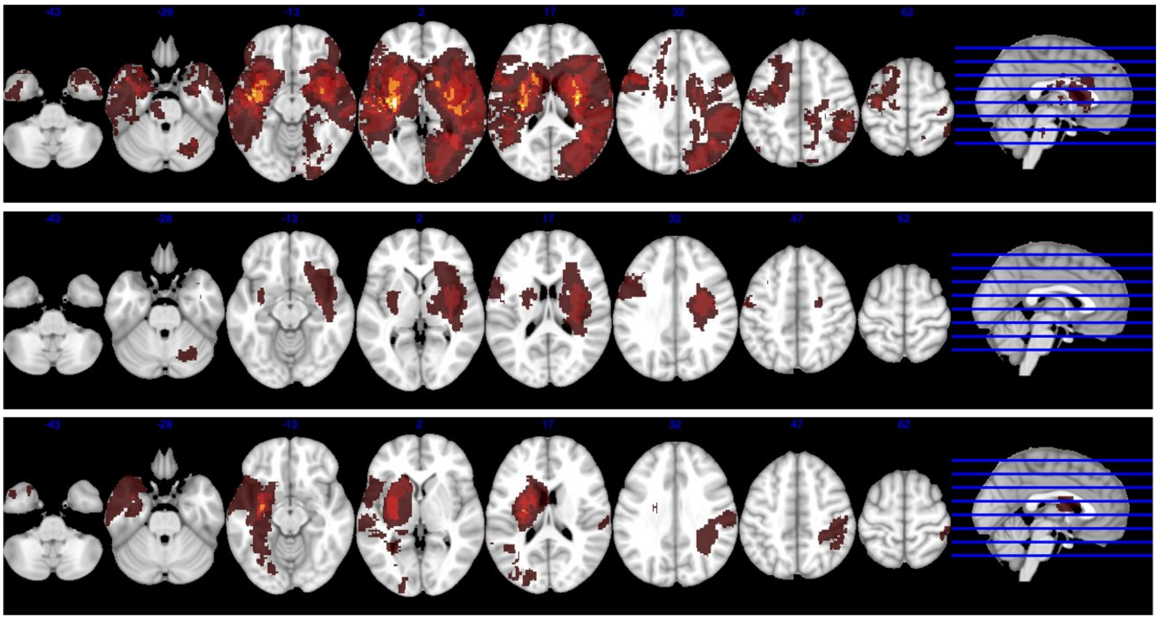

Figure 8. *Frequency maps of lesions related to the 10% (14 subjects) with largest relative estimation errors.*

Reviewer:2

In the current work, the authors aim to investigate post-stroke cognitive and neurological impairment. Specifically, the study sets out to identify how best to classify post-stroke impairment, hypothesizing that impairment will be best described by a low dimensional structure (i.e., a few groups of correlated deficits across different cognitive and physical domains), as has been shown in previous work (Corbetta et al., 2015). In addition, the authors hypothesize that the low dimensional structure of post-stroke impairment will be related to dysfunction of distributed brain networks rather than to damage of a specific area or vascular territory, per se. As predicted, the authors find that ~50% of behavioral variance across patients can be explained by three factors. The authors do not directly test the hypothesis that impairment is related to dysfunction of distributed networks but rather show that the associated lesion topography of the identified components could largely be described by lesion side (i.e.,left vs.right hemisphere damage).
This work makes a contribution to the extant literature on post-stroke neurological impairment. Strengths of the study include that it was conducted prospectively, it has a sizable sample, and it clearly builds and extends prior work in this area. The biggest weakness of the paper that should be addressed is the results of the behavior-anatomy analyses. The authors seem to perform a complex, multivariate analysis but do not spend time adequately explaining and discussing their results. Overall, this paper makes a nice contribution to the literature but there are some major organizational shortcomings that should be addressed to clarify the design and findings of the study before possible publication.
We thank the reviewer for the positive comments.

Major comments:
1. Information regarding sample demographics, inclusion, and other descriptive variables should be addressed in the "Study Sample" section of the paper. Although the authors present much of this information under "Results: Participants," it would be clearer if presented earlier. It may be useful to add a "Procedures" section following "Study Sample" in the "Materials and Methods" section. In the "Results: Participants" section, the authors could immediately present the information on pg. 8, lines 43-58. The corresponding figures/tables should be relabeled accordingly.
We reorganized the information as suggested (see lines 28-34, page 4; lines 1-26 page 5).

2. Information presented in the "Clinical Data" section and "Neuropsychological Battery" section could likely be integrated into the "Procedures" section (see above comment). Another option would be to combine the "Clinical Data" section and "Neuropsychological Battery" into a "Measures" subsection. This reorganization would streamline and clarify the procedures and methods of the study.
We agree with the reviewer. Done as suggested (see lines 28-34, page 4; lines 1-26 page 5).

3. Generally, the supplementary tables and figures are difficult to follow. As the manuscript currently stands, supplementary table 3 is presented in the text following supplementary tables 4 and 5. In addition, supplementary figure 4 is presented before the results and is difficult to interpret since the information is not yet in context to the reader.
We agree with the reviewer. We reorganized the supplementary material to clarify our data: Supplementary Fig. 1 Enrollment Flowchart, Supplementary Fig. 2 Design of the study, Supplementary Fig. 3 Ridge regression procedure, Supplementary Table 1 Demographics of clinical sample, Supplementary Table 2 Acute reperfusion therapy details, Supplementary Table 3 Neuroimaging lesion details, Supplementary Table 4 Harvard-Oxford cortico-

subcortical parcellation, Supplementary Fig. 4 Non-rotated PCA, Supplementary Fig. 5 WU PCA scores, Supplementary Fig. 6 Vascular territories and PC scores.

4. I appreciate the detail provided in the "Lesion-Behavior Analysis" section but feel it is too dense and difficult for a reader to understand. I think the authors should simplify this section, making it more easily understandable, and perhaps, if appropriate, provide the detailed equation information as a supplement. In addition, abbreviations that are not generally known (e.g., L2-normalization; w.r.t.) should be explained.
This section was simplified. The detailed procedure and equations are now in the Supplementary information (Supplementary Figure 3, page 19-20).

5. The authors should consider reorganizing presentation of the behavioral PCA results. Specifically, it might be clearer if the authors present the results shown in figure 2a in the text first and then presents the results shown in figure 2b. I do not necessarily think the authors need to list which subtest loaded onto which component in this section, as that information is in the figure. Rather, it might make sense for the authors to simply provide the summary statement for each component (e.g., PC1 was comprised of measures of language, memory, calculation, apraxia, and allocentric neglect).
We reorganized the presentation of the PCA results, explaining results in Figure 2A and providing a summary statement for each component (see lines 25-32, page 8).

6. When describing PC1 the authors do not mention LOC-questions and LOC-commands in the list of subtests that load onto the component. Was this an oversight or is there a reason for this? LOC-questions and LOC-commands are also not listed in figure 3. In addition, on pg. 10, line 24 the authors state that "the NIHSS presentation were common to PC1 and PC2" but it is unclear where they are drawing this information from.
Thank you. The LOC questions and LOC commands load on PC1 and are now listed in Figure 3. The "OCS" orientation question loaded on both PC1 and 2 (see line 7, page 9).

7. Pg. 10, lines 22-27 are a bit confusing. Can you provide more explanation of why it is notable that some tests positively correlate across two components?
Some behavioral subtests (e.g., NIHSS face palsy, or OCS Heart overall accuracy) were not computed separately for left vs. right body/field, as in the case of arm or leg scores in the NIHSS. Yet, they loaded on both left and right hemisphere specific components. Therefore, these deficits can occur for lesions in either hemisphere (see lines 8-9, page 9)

8. In the initial description of the OCS broken heart test (pg. 5, line 31) the authors describe the test as an attention task. However, later in the results (pg. 10, line 24) and again in the discussion, the authors describe it as a measure of general performance. This should be clarified.
The OCS broken heart test is a test of attention that includes several subtests each measuring a different aspect of attention. The overall accuracy rate (across the two fields) is a measure of sustained attention, which is necessary for a high overall performance. The number of misses either on the left or right visual field is a measure of egocentric neglect. Finally, missing gaps on the left or right side of the individual hearts is a measure of allocentric neglect.
We clarified this description in the Measures Section (see lines 21-26, page 5).

9. The authors should aim to clarify their description of figure 4 both in the text and in the figure caption. It is unclear if the "factor score" referred to on pg.10, line 48 is referring to the behavioral or

anatomical PCA factors. Generally, it is unclear what the authors are trying to convey with the presented scatter plots.
We see the confusion. The scatter plots display the empirically measured behavioral scores (PC score: X-axis) vs. the estimated behavioral scores (model PC scores: Y-axis) from the ridge regression model based on the lesion anatomy. Essentially, the model predicts the best fitting behavioral scores from the distribution of lesioned voxels across patients. If the lesion location is a good predictor, then the relationship between empirical and model scores are linearly related. The R2 scores define the linear fit or behavioral variance accounted for. The figure legend and text have been clarified on lines 18-21 page 9 and lines 15-18 page 17.

10. In the "Validation: WU Cohort" section pg.11, lines 45-59 the authors should use consistent language to the language used to report the results for their sample (i.e., rather than saying "high PC1 scores" say "lower performance on …").
We changed the text accordingly (see lines 20-21; 25;27-28, page 10).

11. The authors conclude that "In general, the topography of damage related to the main axes of behavioral impairment was consistent between both samples of stroke patients" (pg. 12, lines 8-9). However, the effect size of PC3 is quite small. This should perhaps be acknowledged.
The effect size of PC3 was small, we acknowledged this on lines 11-12 page 14.

12. The authors skim over the results of the relationship between the behavioral components and stroke topography. They summarize the findings (pg. 16, lines 36) to what essentially boils down to right vs. left hemispheric locations. A more nuanced discussion of these results, and how they map on to the Corbetta, 2015 results is called for.
We expanded the section in the discussion regarding the cited findings (see lines 21-34 page 15; lines 1-2 page 16). We added a control analysis for vascular distributions (see lines 1-10 page 7; lines 4-18 page 11).

Minor comments:
1. Throughout the manuscript the authors should revise their in-text citations so that the period of the sentence comes after the citation bracket (e.g., pg. 1, line 26: "." should come after (Lyden et al., 2004; Zandieh et al., 2012)).
Done.

2. Pg. 1, line 16: citation for CM Fisher should be (Fisher, 196).
Done.

3. Pg. 5, lines 21-36: The cognitive domains and the names of the subtests on the OCS should not be capitalized.
We agree. Done (lines 13-26, pages 5)

4. Supplementary table 1 does not provide any new information and seems unnecessary.
We removed Supplementary Table 1.

5. The authors should add a table note to supplementary table 4 to explain the coding for the "stroke etiology" variable.
Done (page 23).

6. It is confusing that the authors reference supplementary table 4 on pg. 9, line 12. Perhaps better to just say “(see supplementary tables 4 and 5)” on pg. 9, line 19.
Ok.

7. The authors should add a figure title to supplementary figure 3. In addition, a note should be added explaining the total variance and reporting the p-values of the ridge regressions maps. The way the p-values are presented currently are not clear. In addition, the authors need to explain the different elements of the figure in more detail (either in the text or in the figure note). As written, it is unclear exactly what each element of the figure is showing.
Thank you for the suggestions. We modified Supplementary Figure 3 (now Supplementary Figure 4) to make the results clearer (see lines 1-7 page 32).

8. In figure 2b the authors should list the NIH and OCS subtests on both the right and left sides. It is difficult to see what subtests actually load on to PC1 with the figure’s current layout.
Ok.

9. On figures with brain slices (e.g., Figure 5) the authors should indicate which side is left and which side is right. **Done.**

Editorial

-Please supply a graphical abstract. A graphical abstract is a visual representation of the central finding or methodology of the paper. It will be displayed in the table of contents. Authors should strive to make the graphical abstract informative, interesting, visually appealing, and straightforward (no caption needed, no abbreviations except gene names). Please upload as a separate high resolution image file (tiff preferred). The technical requirements for the graphical abstracts are:
--size of the submitted image: 1200 pixels square at 300dpi
--font of the text: Arial 12-16 point (smaller fonts will not be legible online). **Uploaded.**

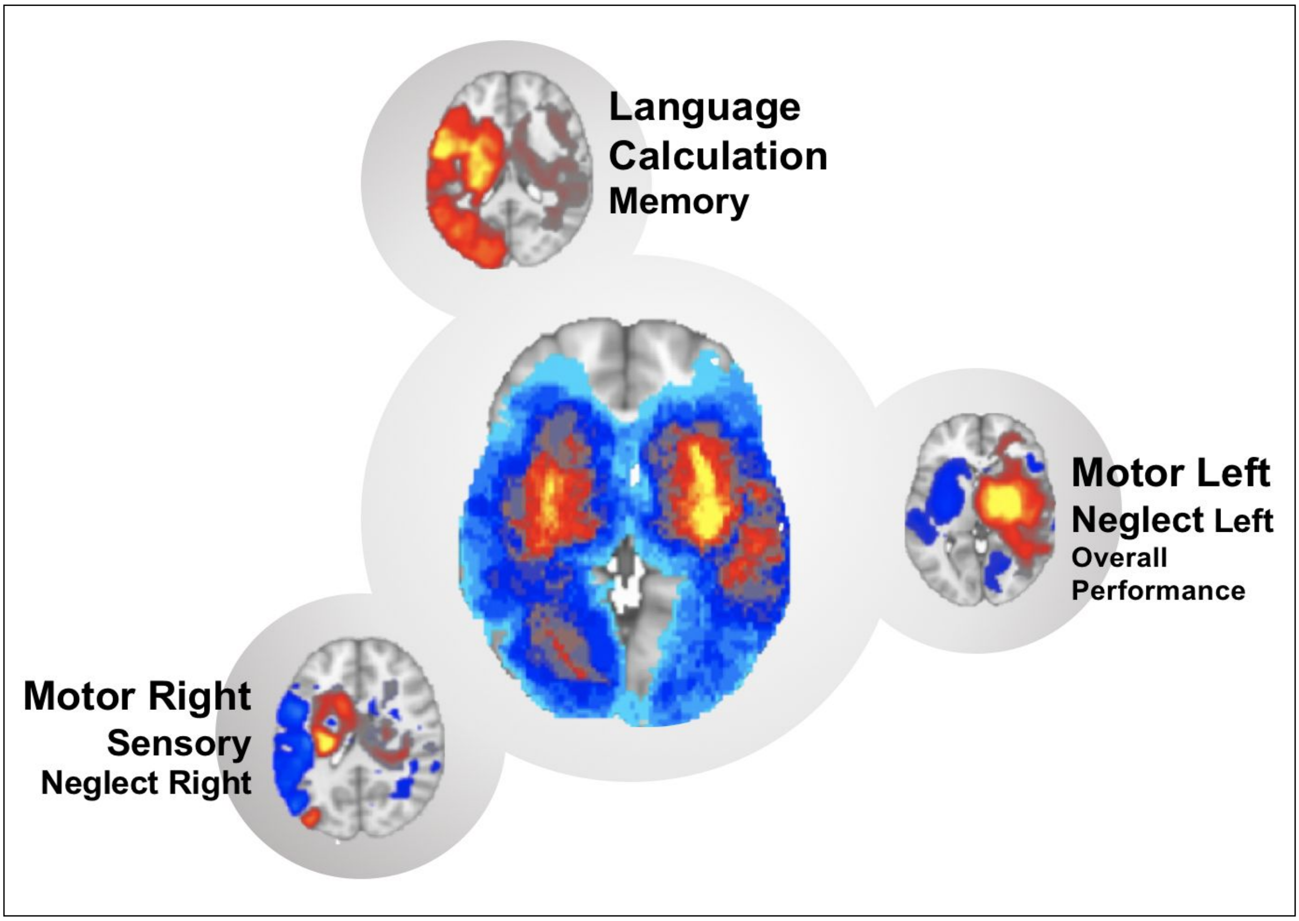

-Please add an Abbreviated Summary (up to 50 words) of your paper that captures the main purpose and conclusions of the work. The summary should state the main results written in the third person ('[1st author surname] et al. report that …') and conclusion. This summary will be used in the contents list online and should not contain any abbreviations other than those on the accepted abbreviations list and accepted gene/protein names, making it accessible to the non-specialist. Please upload it as a Word document. Allowed abbreviations: AIDS; ANOVA; AMPA; ATP; A,T,C,G; CNS; CSF; CT; DNA; ECG; EEG; EMG; GABA; HIV; MRI; NMDA; PET; PCR; RNA.
Neurological deficits following stroke are traditionally described as syndromes related to damage of a specific area or vascular territory. Bisogno et al. show that post-stroke neurological impairment can be described by robust, reproducible components of correlated deficits across functional domains with a bedside, clinically suitable, neurobehavioral assessment.

-Fig4: Please, replace red colour with magenta to make the figure color blind friendly. **Corrected.**

CENTRO DI ATENEO
PADOVA NEUROSCIENCE CENTER

Direttore Prof. Maurizio Corbetta

PADOVA neuroscience CENTER

1222·2022 800 ANNI

UNIVERSITÀ DEGLI STUDI DI PADOVA

We take the opportunity to send our best regards,

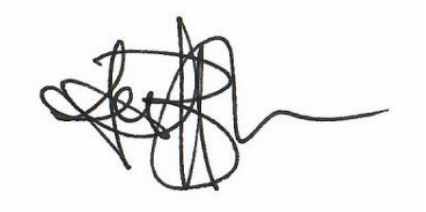

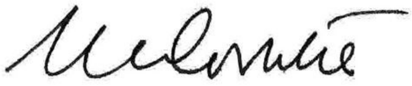

Dr. Antonio Luigi Bisogno
Neurology Resident
Department of Neuroscience
University of Padova, Italy

Prof. Maurizio Corbetta
Chair of Neurology
Director Padova Neuroscience Center
University of Padova, Italy
