# Supplementary material for: A low-dimensional structure of neurological impairment in stroke
Source: Brain Commun. 2021 Jun 3;3(2):fcab119. doi: 10.1093/braincomms/fcab119 (PMC8204367; doi:10.1093/braincomms/fcab119)
Supplement: fcab119_Supplementary_Data [file Revision 1-Decision Letter.pdf › Revision 1-Decision Letter.pdf]

Revision 1-Decision Letter

19-Apr-2021

Dear Dr. Corbetta,

It is a pleasure to accept your manuscript entitled "A low dimensional structure of neurological impairment in stroke" for publication in Brain Communications. Any comments made by the reviewer(s) are included below.

The next step is for your paper to go through “First Look”. We will contact you again shortly if any final files or formatting changes are required for publication or if we have any queries.

If no changes are needed, your submission will be transferred to our Production Department and you will hear from the Production Editor in due course.

Video abstracts - Brain Communications encourages authors to provide a video abstract to accompany their article. These are hosted on the Brain/Brain Communications YouTube channel. If you are interested in producing a 5-min video abstract to promote your article, then please contact the Editorial Office (brcoms.editorialoffice@oup.com) for further details and guidelines.

Thank you very much for your contribution. On behalf of the Editors of Brain Communications, we look forward to your future contributions to the Journal.

Yours sincerely,

Professor Karen Ritchie
Associate Editor
Brain Communications

Reviewer(s)' Comments to Author:

Reviewer: 1 (Aaron Boes)

Comments to the Author
The authors were able to thoughtfully address each of my comments and suggestions.
