# Supplementary material for: A low-dimensional structure of neurological impairment in stroke
Source: Brain Commun. 2021 Jun 3;3(2):fcab119. doi: 10.1093/braincomms/fcab119 (PMC8204367; doi:10.1093/braincomms/fcab119)
Supplement: fcab119_Supplementary_Data [file Original Submission – Decision Letter.pdf › Original Submission – Decision Letter.pdf]

Original Submission – Decision Letter

18-Dec-2020

Dear Dr. Corbetta,

Thank you for your recent submission BRAINCOM-2020-342 entitled "A low dimensional structure of neurological impairment in stroke" to Brain Communications.

Your manuscript has been reviewed, and while the reviewers and editorial team appreciate many aspects of the paper, they raise some concerns that you will need to address in order for the paper to be considered for publication.

If you can address these concerns, I invite you to submit a revised manuscript and a response to each of the reviewers' comments. The reviewer(s)' comments are included at the bottom of this message.

In addition to the reviewers' comments, please note our editorial comments added below to comply with our requirements for presentation of scientific data at Brain Communications.

Please format your revised manuscript according to our Instructions to Authors https://academic.oup.com/braincomms/pages/General_Instructions – a summary of key points is attached to help with this.

To revise your manuscript, click the link below or log into https://mc.manuscriptcentral.com/braincom and enter your Author Centre, where you will find your manuscript title listed under "Manuscripts with Decisions." Under "Actions," click on "Create a Revision." Your manuscript number has been appended to denote a revision.

*** PLEASE NOTE: This is a two-step process. After clicking on the link, you will be directed to a webpage to confirm. ***

https://mc.manuscriptcentral.com/braincom?URL_MASK=517d7636f0cb4f029a7417cda6d28734

Please upload a revised manuscript with changes tracked with track changes or in coloured text.

When submitting your revised manuscript, you will be asked to respond to the reviewer(s) comments. Please be as specific as possible in your response to all of the comments.

The editorial team at Brain Communications aims to increase transparency in scientific publishing and to demonstrate the value that peer review brings to the scientific process. Please therefore consider whether you are willing to have reviewer(s)' comments on your paper and your response published as supplementary data with the paper if it is accepted. This is entirely optional and will only be done if both the authors and reviewers agree. While submitting your revision there will be an opportunity to review or update the answer you gave previously, if necessary.

IMPORTANT: Your original files are available to you when you upload your revised manuscript. Please delete any redundant files before completing the submission.

Because we are trying to facilitate timely publication of manuscripts submitted to Brain Communications, we would appreciate your revised manuscript as soon as possible. If it is not possible for you to resubmit within 3 months, the option to submit a revision will expire, and we would then consider your revised paper as a new submission. However, if you need a little more time, please get in touch and we can extend this deadline for you.

Once again, thank you for submitting your manuscript to Brain Communications and I look forward to receiving your revision.

Yours sincerely,

Prof. Karen Ritchie
Editor, Brain Communications

Reviewer(s)' Comments to Author:

Reviewer: 1 (Aaron Boes)

Comments to the Author

In this article by Bisogno and colleagues perform a prospective lesion mapping experiment in 237 first-time stroke patients to evaluate the structure of behavioral deficits using principal component analysis and lesion symptom maps of the resulting behavioral clusters. In doing so, the authors demonstrate three principal components that explain about 50% of the variance in behavioral outcomes: PC1 includes language, calculation, praxis, right-sided neglect, and memory; PC2 includes left motor, visual, and spatial neglect, and PC3 included right motor impairment. Importantly this same basic pattern of behavioral structure and lesion localization is present in a large independent cohort evaluated by the senior author previously (Corbetta, 2015).

Overall this article is well-done and has several strengths. This includes a large sample size, a prospective design with well-defined inclusion and exclusion criteria that match the prior 2015 cohort, a strong statistical design for dealing with behavior and lesion-behavior mapping, and a cross-cohort analysis to further support the main findings. The findings are directly relevant to ongoing efforts in the field to develop better prognostic tools for stroke outcomes.

I don’t see any major flaws in the study design. The methods are well suited to address the questions being asked and the conclusions are aligned with the results. I have a few questions and points of clarification that may help to improve the final manuscript.

In the abstract the authors report that: ‘post-stroke neurological impairment is best described by a few groups of correlated deficits across different domains that relate to the dysfunction of distributed brain networks.’ It might be more accurate to say that recent studies have shown that much of the variance in post-stroke outcomes can be explained by a few groups of correlated deficits. It seems premature to conclude that this is the ‘best’ way to characterize post-stroke neurological impairment.

The authors report that neurological deficits relate to widespread network impairment rather than damage to a specific area or vascular territory. This statement appears to be largely made based on prior work and not the current analysis. More details supporting this claim would be helpful. Given the significant lesion-symptom mapping results in this analysis and the similarity to prior results (Corbetta, 2015) it appears these neurological deficits are reliably related to lesion location, even if the localization is regional as opposed to specific structures or specific vascular territories. The authors don’t evaluate this topic of specific vascular distributions directly as far as I can tell. Are the authors confident that certain branches of the MCA are not associated with more pronounced deficits in the three major components?

The introductory comments appear to draw a contrast between the historical identification of stroke syndromes and the more recent elucidation of low dimensional clusters of correlated stroke symptoms. These newer PCA results, in my view, don’t invalidate or diminish the existence of lesion syndromes. The two approaches don’t seem diametrically opposed. Isn’t it the case that these two broad strategies just describe stroke symptoms with different levels of granularity, with the low dimensional clusters explaining a large portion of the variance in outcomes, which is likely to be useful in some research and clinical applications?

The authors apply a spatial PCA that captures 95% of the variance in the matrix of voxels damaged. I would like to have more details about the result of this data reduction strategy. Does this eliminate some regions with minimal lesion overlap (e.g. cerebellum, brainstem) from the multivariate model, or does it simply increase the size of smallest units entering the model?

What were the numbers of MRI versus CT scans?

The predictive models perform less accurately for small lesions. Is this because these small lesions tend to occur in statistically underpowered regions outside the MCA distribution (brainstem. cerebellum), or does it reflect a more intact ability to compensate for deficits following a small lesion?

Reviewer: 2

Comments to the Author
In the current work, the authors aim to investigate post-stroke cognitive and neurological impairment. Specifically, the study sets out to identify how best to classify post-stroke impairment, hypothesizing that impairment will be best described by a low dimensional structure (i.e., a few groups of correlated deficits across different cognitive and physical domains), as has been shown in previous work (Corbetta et al., 2015). In addition, the authors hypothesize that the low dimensional structure of post-stroke impairment will be related to dysfunction of distributed brain networks rather than to damage of a specific area or vascular territory, per se. As predicted, the authors find that ~50% of behavioral variance across patients can be explained by three factors. The authors do not directly test the hypothesis that impairment is related to dysfunction of distributed networks but rather show that the

associated lesion topography of the identified components could largely be described by lesion side (i.e., left vs. right hemisphere damage).
This work makes a contribution to the extant literature on post-stroke neurological impairment. Strengths of the study include that it was conducted prospectively, it has a sizable sample, and it clearly builds and extends prior work in this area. The biggest weakness of the paper that should be addressed is the results of the behavior-anatomy analyses. The authors seem to perform a complex, multivariate analysis but do not spend time adequately explaining and discussing their results. Overall, this paper makes a nice contribution to the literature but there are some major organizational shortcomings that should be addressed to clarify the design and findings of the study before possible publication.

Major comments:
1. Information regarding sample demographics, inclusion, and other descriptive variables should be addressed in the "Study Sample" section of the paper. Although the authors present much of this information under "Results: Participants," it would be clearer if presented earlier. It may be useful to add a "Procedures" section following "Study Sample" in the "Materials and Methods" section. In the "Results: Participants" section, the authors could immediately present the information on pg. 8, lines 43-58. The corresponding figures/tables should be relabeled accordingly.
2. Information presented in the "Clinical Data" section and "Neuropsychological Battery" section could likely be integrated into the "Procedures" section (see above comment). Another option would be to combine the "Clinical Data" section and "Neuropsychological Battery" into a "Measures" subsection. This reorganization would streamline and clarify the procedures and methods of the study.
3. Generally, the supplementary tables and figures are difficult to follow. As the manuscript currently stands, supplementary table 3 is presented in the text following supplementary tables 4 and 5. The authors should take care to order the supplementary tables in the order in which they are presented in the text. In addition, supplementary figure 4 is presented before the results and is difficult to interpret since the information is not yet in context to the reader.
4. I appreciate the detail provided in the "Lesion-Behavior Analysis" section but feel it is too dense and difficult for a reader to understand. I think the authors should simplify this section, making it more easily understandable, and perhaps, if appropriate, provide the detailed equation information as a supplement. In addition, abbreviations that are not generally known (e.g., L2-normalization; w.r.t.) should be explained.
5. The authors should consider reorganizing presentation of the behavioral PCA results. Specifically, it might be clearer if the authors present the results shown in figure 2a in the text first and then presents the results shown in figure 2b. I do not necessarily think the authors need to list which subtest loaded onto which component in this section, as that information is in the figure. Rather, it might make sense for the authors to simply provide the summary statement for each component (e.g., PC1 was comprised of measures of language, memory, calculation, apraxia, and allocentric neglect).
6. When describing PC1 the authors do not mention LOC-questions and LOC-commands in the list of subtests that load onto the component. Was this an oversight or is there a reason for this? LOC-questions and LOC-commands are also not listed in figure 3. In addition, on pg. 10, line 24 the authors state that "the NIHSS presentation were common to PC1 and PC2" but it is unclear where they are drawing this information from.
7. Pg. 10, lines 22-27 are a bit confusing. Can you provide more explanation of why it is notable that some tests positively correlate across two components?
8. In the initial description of the OCS broken heart test (pg. 5, line 31) the authors describe the test as an attention task. However, later in the results (pg. 10, line 24) and again in the discussion, the authors describe it as a measure of general performance. This should be clarified.
9. The authors should aim to clarify their description of figure 4 both in the text and in the figure caption. It is unclear if the "factor score" referred to on pg.10, line 48 is referring to the behavioral or anatomical PCA factors. Generally, it is unclear what the authors are trying to convey with the presented scatter plots.
10. In the "Validation: WU Cohort" section pg.11, lines 45-59 the authors should use consistent language to the language used to report the results for their sample (i.e., rather than saying "high PC1 scores" say "lower performance on …").
11. The authors conclude that "In general, the topography of damage related to the main axes of behavioral impairment was consistent between both samples of stroke patients" (pg. 12, lines 8-9). However, the effect size of PC3 is quite small. This should perhaps be acknowledged.
12. The authors skim over the results of the relationship between the behavioral components and stroke topography. They summarize the findings (pg. 16, lines 36) to what essentially boils down to right vs. left hemispheric locations. A more nuanced discussion of these results, and how they map on to the Corbetta, 2015 results is called for.

Minor comments:
1. Throughout the manuscript the authors should revise their in-text citations so that the period of the sentence comes after the citation bracket (e.g., pg. 1, line 26: "." should come after (Lyden et al., 2004; Zandieh et al., 2012)).
2. Pg. 1, line 16: citation for CM Fisher should be (Fisher, 196).

3. Pg. 5, lines 21-36: The cognitive domains and the names of the subtests on the OCS should not be capitalized.
4. Supplementary table 1 does not provide any new information and seems unnecessary.
5. The authors should add a table note to supplementary table 4 to explain the coding for the "stroke etiology" variable.
6. It is confusing that the authors reference supplementary table 4 on pg. 9, line 12. Perhaps better to just say "(see supplementary tables 4 and 5)" on pg. 9, line 19.
7. The authors should add a figure title to supplementary figure 3. In addition, a note should be added explaining the total variance and reporting the p-values of the ridge regressions maps. The way the p-values are presented currently are not clear. In addition, the authors need to explain the different elements of the figure in more detail (either in the text or in the figure note). As written, it is unclear exactly what each element of the figure is showing.
8. In figure 2b the authors should list the NIH and OCS subtests on both the right and left sides. It is difficult to see what subtests actually load on to PC1 with the figure's current layout.
9. On figures with brain slices (e.g., Figure 5) the authors should indicate which side is left and which side is right.

Editorial Office comments:

-Please supply a graphical abstract. A graphical abstract is a visual representation of the central finding or methodology of the paper. It will be displayed in the table of contents. Authors should strive to make the graphical abstract informative, interesting, visually appealing, and straightforward (no caption needed, no abbreviations except gene names). Please upload as a separate high resolution image file (tiff preferred). The technical requirements for the graphical abstracts are:
--size of the submitted image: 1200 pixels square at 300dpi
--font of the text: Arial 12-16 point (smaller fonts will not be legible online)

-Please add an Abbreviated Summary (up to 50 words) of your paper that captures the main purpose and conclusions of the work. The summary should state the main results written in the third person ('[1st author surname] et al. report that …') and conclusion. This summary will be used in the contents list online and should not contain any abbreviations other than those on the accepted abbreviations list and accepted gene/protein names, making it accessible to the non-specialist. Please upload it as a Word document. Allowed abbreviations: AIDS; ANOVA; AMPA; ATP; A,T,C,G; CNS; CSF; CT; DNA; ECG; EEG; EMG; GABA; HIV; MRI; NMDA; PET; PCR; RNA.

-Fig4: Please, replace red colour with magenta to make the figure color blind friendly.
